# Novel Mycoviruses Discovered in the Mycovirome of a Necrotrophic Fungus

**DOI:** 10.1128/mBio.03705-20

**Published:** 2021-05-11

**Authors:** Ana Ruiz-Padilla, Julio Rodríguez-Romero, Irene Gómez-Cid, Davide Pacifico, María A. Ayllón

**Affiliations:** a Centro de Biotecnología y Genómica de Plantas, Universidad Politécnica de Madrid/Instituto Nacional de Investigación y Tecnología Agraria y Alimentaria (INIA), Pozuelo de Alarcón, Madrid, Spain; b Departamento de Biotecnología-Biología Vegetal, Escuela Técnica Superior de Ingeniería Agronómica, Alimentaria y de Biosistemas, Universidad Politécnica de Madrid, Madrid, Spain; c Institute of Bioscience and Bioresources, National Research Council of Italy, Palermo, Italy; National Institutes of Health

**Keywords:** binarnavirus, ssDNA mycovirus, trisegmented ssRNA– virus, virus metagenomics, virome, mycovirome, fungi, plant pathogens, *Botrytis cinerea*, grapevine

## Abstract

*Botrytis cinerea* is one of the most important plant-pathogenic fungus. Products based on microorganisms can be used in biocontrol strategies alternative to chemical control, and mycoviruses have been explored as putative biological agents in such approaches. Here, we have explored the mycovirome of *B. cinerea* isolates from grapevine of Italy and Spain to increase the knowledge about mycoviral diversity and evolution, and to search for new widely distributed mycoviruses that could be active ingredients in biological products to control this hazardous fungus. A total of 248 *B. cinerea* field isolates were used for our metatranscriptomic study. Ninety-two mycoviruses were identified: 62 new mycoviral species constituting putative novel viral genera and families. Of these mycoviruses, 57 had a positive-sense single-stranded RNA (ssRNA) genome, 19 contained a double-stranded RNA (dsRNA) genome, 15 had a negative-sense ssRNA genome, and 1 contained a single-stranded DNA (ssDNA) genome. In general, ssRNA mycoviruses were widely distributed in all sampled regions, the ssDNA mycovirus was more frequently found in Spain, and dsRNA mycoviruses were scattered in some pools of both countries. Some of the identified mycoviruses belong to clades that have never been found associated with *Botrytis* species: *Botrytis*-infecting narnaviruses; alpha-like, umbra-like, and tymo-like ssRNA+ mycoviruses; trisegmented ssRNA− mycovirus; bisegmented and tetrasegmented dsRNA mycoviruses; and finally, an ssDNA mycovirus. Among the results obtained in this massive mycovirus screening, the discovery of novel bisegmented viruses, phylogenetically related to narnaviruses, is remarkable.

## INTRODUCTION

Metagenomics has been used to detect viruses already identified or to discover novel viruses in different environments or hosts, including fungi, showing the high variability of viruses present in the virosphere and increasing the knowledge regarding their horizontal transfer between hosts (1–9). Fungi are hosts of mycoviruses with different types of genomes, mainly of double-stranded RNA (dsRNA) and positive-sense single-stranded RNA (ssRNA+) (10). Moreover, negative-sense single-stranded RNA (ssRNA–) viruses have been recently found infecting different genera of fungi (4, 11, 12). However, in the last decade only two mycoviruses with single-stranded DNA (ssDNA) genomes have been identified as infecting the plant-pathogenic fungi Sclerotinia sclerotiorum and Fusarium graminearum (13, 14). There are examples of mycoviruses related to families of viruses that infect other hosts. Among ssRNA+ mycoviruses, since the discovery of the first ourmia-like mycoviruses (3, 5, 15) related to plant ourmiaviruses (16), several similar ourmia-like mycoviruses have been discovered (17–19); also, some of the identified ssRNA– mycoviruses are related to plant or animal viruses (4, 11, 20). However, a clear example of host change is the finding of the plant cucumber mosaic virus inside the plant-pathogenic fungus Rhizoctonia solani (6). These and other examples revealed horizontal transfer associated with virus evolution. Indeed, viral metagenomics studies are increasing the knowledge about mycoviral evolution and contribute to the identification of new viruses infecting fungi in different hosts. However, compared to other hosts, only a very few studies have explored the mycovirome through metatranscriptomic characterizations (5, 8, 9, 21–28).

*Botrytis cinerea* Pers.:Fr. (teleomorph *Botryotinia fuckeliana* [de Bary] Whetzel) is considered the second most significant fungal plant pathogen and is an excellent model for the study of the infectious process for necrotrophic fungi (29). This fungus causes gray mold or gray rot in more than 200 crops worldwide, and it has no apparent host specificity (30). It causes substantial economic losses in important crops (grapevine, strawberry, tomato, eggplant, cucumber, zucchini, bean, pepper, etc.) both in plants in the field and in fruits in postharvest. Unfortunately, *B. cinerea* control is difficult due to the different forms of infection, diverse hosts acting as inoculum sources, and its ability to survive as conidia, sterile mycelia, or resistant sclerotia for long periods of time (31). Different strategies have been used for fungal control, including cultural practices, biological control agents, host resistance, and fungicides. The application of fungicides is extensively used, and in many crops the large amount that should be applied leads to a rapid development of fungal resistance to fungicides and has a negative impact in the environment (32, 33). In addition, global climatic change is prompting to find new strategies for fungal control that may appear as a consequence of environmental conditions. In such new circumstances, alternatives to the chemical control must be mandatory in order to apply more environmentally friendly products. To date, several botrycide products, based on microorganisms as active ingredients, have been developed for *B. cinerea* biocontrol (34, 35). The discovery of new viruses infecting fungal hosts could offer a new possibility as tools for biological control, since some of those already characterized decrease fungal virulence. One clear example is the successful use of hypovirulent isolates of *Cryphonectria parasitica* infected with mycoviruses (Cryphonectria parasitica hypovirus 1 [CHV1]) for the biological control of chestnut blight (36).

Interestingly, RNA mycoviruses are widespread in *Botrytis* species, and some of them can attenuate virulence on their fungal hosts. To date, mycoviruses have been researched in *B. cinerea* collections from various regions of the world, using different methodologies such as dsRNA extraction and viral metagenomics, showing in some cases a quite complex mycovirome (23, 37, 38). Most mycoviruses infecting *B. cinerea* have dsRNA or ssRNA+ genomes (39), and some mycoviruses with an ssRNA–genome have been also found infecting *Botrytis* isolates from different hosts (4, 12), but no ssDNA mycoviruses have yet been identified to infect this fungus. In addition, several mycoviruses have been already associated with hypovirulence in *B. cinerea* isolates from different countries and hosts, indicating the possibility of a biocontrol strategy of the fungus using mycoviruses. For instance, Botrytis cinerea mitovirus 1 (40), Botrytis cinerea hypovirus 1 (41), Botrytis cinerea mymonavirus 1 (12), Botrytis cinerea CCg378 mycovirus (42), Botrytis cinerea RNA virus 1 (43), and Botrytis cinerea partitivirus 2 (44, 45) have been shown to be associated with a reduced virulence of *B. cinerea*.

To date, an extensive study of *Botrytis* species mycovirome has never been accomplished. Grapevine is one of the main hosts of *B. cinerea*, and Italy and Spain are two of the most important wine-producing countries in the world. The purpose of the present study was to explore the mycovirome of *B. cinerea* isolates from infected vineyards in different Italian and Spanish regions in order to discover widely disseminated novel mycoviruses. In addition to finding novel viruses, a large collection of *B. cinerea* mycoviruses has been created to evaluate its future potential use in biological control approaches. Here, 248 *B. cinerea* isolates were used, most of them infected by mycoviruses with different types of genomes (dsRNA, ssRNA+, ssRNA–, and ssDNA mycoviruses); some of them have already been described to cause hypovirulence in *Botrytis* spp., *S. sclerotiorum*, or other fungal genera. More than half of the discovered mycoviruses in our collection were present in both Italy and Spain and may be potential agents to use in biocontrol strategies of the fungus worldwide. Interestingly, unique mycoviruses infecting *B. cinerea* have been characterized here as novel ssRNA– (monosegmented and a trisegmented mycoviruses), dsRNA (quadrivirus and bipartite dsRNA mycoviruses), and ssRNA+ (umbra-like, alpha-like, and tymo-like mycoviruses, narnaviruses, and binarnaviruses) mycoviruses and as an ssDNA mycovirus. The findings presented here represent an important contribution to the knowledge of *B. cinerea* mycoviruses.

## RESULTS

### Identification of mycoviral sequences in *B. cinerea* isolates.

A total of 384 samples of the fungus *B. cinerea* infecting grapevine were collected from several vineyards across Italy and Spain and isolated in *in vitro* cultures. Among them, 248 samples, 150 from Spain and 98 from Italy, were selected for further analyses. Initially, all fungal samples were analyzed by qPCR using as the template DNA and specific primers designed for the detection of the species “*cinerea*” of the genus *Botrytis*, and once all of them were confirmed as *B. cinerea* isolates (data not shown), the samples were distributed in 17 Spanish (*B. cinerea* Spain, BCS1 to BCS17) and 12 Italian (*B. cinerea* Italy, BCI1 to BCI12) pools. For the 29 pools, a total of 2,696M reads were obtained, with close to 93M reads on average per pool. After trimming and decontamination, cleaned reads of each pool were assembled by a “*de novo*” mRNA transcript assembly software. Most contigs mapped to the genome of the host *B. cinerea*, with some sequences mapping to other *Botrytis* species (data not shown). Contigs from each pool of samples were analyzed separately using BLASTx against a nonredundant protein database to identify specific mycoviromes associated with each one, resulting in 29 lists of mycoviral sequences (not shown), one per pool. Only contigs with a length of >1,000 bp were considered for the analysis, but the remaining sequences under this fixed size were revised to ensure that only redundant information was eliminated. In total, 1,269 mycoviral sequences passed the filter size, 670 sequences from BCS pools and 599 sequences from BCI pools. These contigs were filtered for redundancy at a 90% nucleotide identity over 90% of the length, and representative mycoviral sequences were selected from each pool, which were the longest assembled sequence of a group of mycoviruses that have more than 90% of identity at the nucleotide level when comparing with all sequences inside each pool. This selection reduced the number of identified mycoviral sequences from 1,269 to 158 in a single list for all *B. cinerea* pools, that in summary corresponded to 92 viruses as described below. The number of sequences was reduced from 158 to 109 unique mycoviral sequences, 79 from Spanish pools and 30 from Italian pools, by selection of complete coding sequences with the longest nucleotide lengths. Of the 109 unique mycoviral sequences, 19 correspond to mycoviruses already described in the databases: 13 of them were annotated as variants of described mycoviruses, and the remaining 6 sequences were not deposited again in the database, and their original accession numbers were maintained. Finally, this 109 mycoviral sequences corresponded to 92 mycoviruses, mono- or multisegmented; 11 of these were considered variants of three new identified mycoviruses, since the identity at the amino acid level was >95%, and the genomic organization was identical. These variants were named with the given name of the representative mycovirus followed by the name of the pool. The summary list of the new mycoviruses detected is included in Table 1. All of the raw sequencing reads were stored in the Sequence Read Archive (SRA) database: BioProject accession no. PRJNA632510, BioSample accession numbers from SAMN14911182 to SAMN14911210, and SRA runs from SRX8335942 to SRX8335970.

**TABLE 1:** Mycoviruses identified in *B. cinerea* samples^*a*^

Virus taxon	Accession no.	Genome segment	Genome polarity^*b*^	Conserved domain	Name	Isolate identifier	Contig identifier	Length (bp)	Length (aa)	mol wt (kDa)	E value	First hit (nr NCBI)	BLASTx results	Identity (%)
dsRNA viruses	MN617031	RNA1	dsRNA	RdRp	Botrytis cinerea mycovirus 3 (BcMyV3)	BCS6	BCS6_TRINITY_DN7828_c0_g1_i1	2,024	607	69.28	0.0	KC549809	C. parasitica bipartite mycovirus 1	60.00
	MN617032	RNA2		HP		BCS4	BCS4_TRINITY_DN4111_c0_g1_i1	1,780	307	33.15	4,00E-78	KC549809	C. parasitica bipartite mycovirus 1	46.47
*Botybirnavirus*	MN627274	RNA1	dsRNA	RdRp	Botrytis cinerea botybirnavirus 2 (BcBV2)	BCI11	BCS17_TRINITY_DN61_c0_g2_i1	6,038	1,831	205	0.0	MH684534	B. dothidea botybirnavirus 1	53.16
	MN627275	RNA2		HP		BCS17	BCS17_TRINITY_DN61_c0_g1_i1	5,936	1,801	200.3	0.0	MH321500	B. cinerea botybirnavirus 1	32.53
*Botybirnavirus*	MN954879	RNA1	dsRNA	RdRp	Botrytis porri botybirnavirus 1 (BpBV1)	BCS16	BCS16_TRINITY_DN64_c0_g2_i1	6,201	1,902	213.2	0.0	JF716350	B. porri botybirnavirus 1	97.32
	MN954880	RNA2		HP		BCS16	BCS16_TRINITY_DN64_c0_g1_i1	5,802	1,788	197.47	0.0	JF716351		97.48
dsRNA viruses	MN617757	RNA1	dsRNA	RdRp	Botrytis cinerea mycovirus 5 (BcMyV5)	BCI6	BCI6_Contig7	2,184	675	76.31	0.0	KX380787	F. graminearum dsRNA mycovirus 5	83.44
	MN617755	RNA2	dsRNA	HP	Botrytis cinerea mycovirus 5 (BcMyV5)	BCI7	BCI7_TRINITY_DN6668_c0_g1_i2	1,522	316	34.75	4E–154	KX380788		73.84
	MN617756	RNA2	dsRNA	HP	Botrytis cinerea mycovirus 5 (BcMyV5)	BCI6	BCI6_TRINITY_DN3037_c0_g1_i2	1,433	344	38.11	1E–148	KX380788		66.87
*Partitiviridae*	MN954881	RNA1	dsRNA	RdRp	Botryotinia fuckeliana partitivirus 1 (BfPV1)	BCS3	BCS3_TRINITY_DN4616_c0_g1_i1	1,780	540	62.68	0.0	AM491609	B. fuckeliana partitivirus 1	99.63
	MN954882	RNA2		CP		BCI12	BCI12_TRINITY_DN10399_c0_g1_i1	1,597	436	47.11	0.0	AM491610		95.64
*Quadrivirus*	MN954886	RNA1	dsRNA	HP	Botrytis cinerea mycovirus 4 (BcMyV4)	BCS14	BCS14_TRINITY_DN1846_c0_g1_i1	4,983	1,592	177.32	0.0	MH347279	B. cinerea RNA Virus 2	90.76
	MN954885	RNA3		RdRp		BCS14	BCS14_Contig45	4,267	1,364	152.35	0.0	MH347280		95.31
	MN617034	RNA4		HP		BCS14	BCS14_TRINITY_DN1605_c0_g1_i1	3,944	1,128	85.78	0.0	MH347281		96.79
	MN617035	RNA2		Structural protein		BCS13	BCS13_TRINITY_DN3888_c0_g1_i1	4,401	1,407	154.6	9.00E–07	AB620062	R. necatrix quadrivirus 1	23.63
*Victorivirus*	MH347278	RNA	dsRNA	CP incomplete	Botrytis cinerea victorivirus 1 (BcVV1)	BCS15	BCS15_TRINITY_DN531_c0_g3_i1	2,539	785	85.73	0.0	MH347278	B. cinerea victorivirus 1	90.81
*Victorivirus*	MN617038	RNA	dsRNA	RdRp	Botrytis cinerea victorivirus 2 (BcVV2)	BCS11	BCS11_TRINITY_DN2674_c0_g1_i1	5,186	807	85.78	0.0	AM491608	B. fuckeliana totivirus 1	85.08
				CP					838	92.51	0.0	MH347278	B. cinerea victorivirus 1	91.43
*Victorivirus*	MN617037	RNA	dsRNA	RdRp	Botrytis cinerea victorivirus 2-BCS9	BCS9	BCS9_TRINITY_DN1144_c0_g1_i1	5,184	838	92.79	0.0	AM491608	B. fuckeliana totivirus 1	85.44
				CP					807	85.82	0.0			93.59
*Victorivirus*	MN839444	RNA	dsRNA	RdRp	Botrytis cinerea victorivirus 2-BCS14	BCS14	BCS14_TRINITY_DN8324_c0_g1_i1	5,173	838	92.39	0.0	AM491608	B. fuckeliana totivirus 1	85.92
				CP					807	85.79	0.0			93.86
*Victorivirus*	MN839445	RNA	dsRNA	RdRp	Botrytis cinerea victorivirus 2-BCS12	BCS12	BCS12_TRINITY_DN1502_c0_g1_i1	5,201	838	92.53	0.0	AM491608	B. fuckeliana totivirus 1	86.16
				CP					807	85.78	0.0			93.32
*Victorivirus*	MN839446	RNA	dsRNA	RdRp	Botrytis cinerea victorivirus 3-BCS17	BCS17	BCS17_TRINITY_DN10534_c0_g1_i1	5,173	838	92.35	0.0	AM491608	B. fuckeliana totivirus 1	86.16
				CP					807	85.79	0.0			94.50
*Victorivirus*	MN839447	RNA	dsRNA	RdRp	Botrytis cinerea victorivirus 2-BCS8	BCS8	BCS8_TRINITY_DN1217_c0_g1_i1	5,177	838	92.76	0.0	AM491608	B. fuckeliana totivirus 1	85.08
				CP					807	85.52	0.0			93.33
*Victorivirus*	MN839448	RNA	dsRNA	RdRp	Botrytis cinerea victorivirus 2-BCI8	BCS8	BCS8_TRINITY_DN1217_c0_g2_i1	4,360	838	92.7	0.0	AM491608	B. fuckeliana totivirus 1	85.68
				CP					538	57.24	0.0			92.92
*Victorivirus*	MN839449	RNA	dsRNA	RdRp	Botrytis cinerea victorivirus 3 (BcVV3)	BCS9	BCS9_TRINITY_DN1144_c0_g1_i2	5,205	838	92.34	0.0	AM491608	B. fuckeliana totivirus 1	86.40
				CP					807	85.73	0.0	MH347278	B. cinerea victorivirus 1	91.20
*Victorivirus*	MN839450	RNA	dsRNA	RdRp	Botrytis cinerea victorivirus 3-BCS13	BCS13	BCS13_TRINITY_DN4062_c0_g1_i1	5,178	838	92.28	0.0	AM491608	B. fuckeliana totivirus 1	85.68
				CP					807	85.67	0.0	MH347278	B. cinerea victorivirus 1	91.82
*Victorivirus*	MN839451	RNA	dsRNA	RdRp	Botrytis cinerea victorivirus 3-BCS16	BCS16	BCS16_TRINITY_DN5_c0_g1_i2	5,171	838	92.42	0.0	AM491608	B. fuckeliana totivirus 1	85.68
				CP					807	85.69	0.0	MH347278	B. cinerea victorivirus 1	91.45
dsRNA virus	LN827952	RNA	dsRNA	RdRp	Sclerotinia sclerotiorum dsRNA mycovirus L (SsNsV‐L)	BCS12	BCS12_TRINITY_DN3578_c0_g1_i1	8,914	1,338	146.33	0.0	LN827952	S. sclerotiorum dsRNA mycovirus L	96.94
				HP					1,306	144.52	0.0			92.87
*Gammapartitivirus*	MN954883	RNA2	dsRNA	CP	Botrytis cinerea partitivirus 3 (BcPV3)	BCS4	BCS4_TRINITY_DN5031_c0_g1_i1	1,537	433	45.88	0.0	MF444213	S. sclerotiorum partitivirus 2	92.84
	MN954884	RNA1		RdRp		BCS4	BCS4_TRINITY_DN10017_c0_g1_i1	1,762	539	62.67	0.0	MF444214	S. sclerotiorum partitivirus 3	99.64
*Alphavirus*	MN625250	RNA	ssRNA(+)	Polyprotein	Botrytis cinerea alpha-like virus 1 (BcAV1)	BCI2	BCI2_Contig12	8,008	1,975	219.85	1E–09	MH766488	S. rolfsii alphavirus-like virus 1	35
				HP1					185	20.74			No significant similarity found	
				HP2					230	25.52			No significant similarity found	
*Flexiviridae*	MN625248	RNA	ssRNA(+)	RdRp	Botrytis cinerea flexivirus 1 (BcFlV1)	BCI5	BCI5_contig11	13,985	4,619	439.58	0.0	MK584823	L. chartarum flexivirus 1	33.94
*Deltaflexivirus*	MN625249	RNA	ssRNA(+)	RdRp	Botrytis cinerea deltaflexivirus 1 (BcDFV1)	BCS16	BCS16_TRINITY_DN4173_c0_g1_i1	4,869	1,124	124.95	0.0	KT598226	Soybean leaf-associated mycoflexivirus 1	41.32
				HP					193	20.5	4.00E–08	KT598226		28.41
*Deltaflexivirus*	MN954874	RNA	ssRNA(+)	RdRp	Sclerotinia sclerotiorum deltaflexivirus 2 (SsDFV2)	BCS1	BCS1_TRINITY_DN9681_c0_g1_i1	6,628	2,086	232.66	0.0	MH299810	S. sclerotiorum deltaflexivirus 2	92.71
*Umbravirus*	MN625251	RNA	ssRNA(+)	RdRp	Botrytis cinerea umbra-like virus 1 (BcUV1)	BCS1	BCS1_TRINITY_DN12780_c0_g1_i1	3,865	519	58.3	0.0	KC601995	S. sclerotiorum umbra-like virus 1	50.77
				HP					338	36.7	8E−26			
*Umbravirus*	MT230951	RNA	ssRNA(+)	RdRp	Sclerotinia sclerotiorum umbra-like virus 2 (SsUV2)	BCS2	BCS2_TRINITY_DN3109_c0_g1_i1	4,752	344	38.2	0.0	MF444273	S. sclerotiorum umbra like virus 2	95.78
				RaP					528	59.83				
*Umbravirus*	MT230952	RNA	ssRNA(+)	RdRp	Sclerotinia sclerotiorum umbra-like virus 3 (SsUV3)	BCS17	BCS17_TRINITY_DN25_c0_g1_i4	3,981	417	46.42	0.0	MF444274	S. sclerotiorum umbra like virus 3	98.17
				RaP					513	58.19	0.0			96.40
*Endornaviridae*	MN617758	RNA	ssRNA(+)	Polyprotein	Botrytis cinerea endornavirus 2 (BcEV2)	BCI1	BCI1_TRINITY_DN2153_c0_g1_i2	13,581	4,501	504.55	0.0	MG255170	S. minor endornavirus 1	87.84
*Endornaviridae*	MN839443	RNA	ssRNA(+)	Polyprotein	Botrytis cinerea endornavirus 3 (BcEV3)	BCI11	BCI11_TRINITY_DN9384_c0_g1_i1	13,582	4,501	504.6	0.0	MG255170	S. minor endornavirus 1	88.20
*Fusarivirirus*	MN617762	RNA	ssRNA(+)	RdRp	Botrytis cinerea fusarivirus 3 (BcFV3)	BCS15	BCS15_TRINITY_DN2871_c0_g2_i1	8,354	1,657	189.39	0.0	MK558256	R. solani fusarivirus 2	34.27
				HP					704	78.37	0.0			45.87
*Fusarivirirus*	MN617763	RNA	ssRNA(+)	RdRp	Botrytis cinerea fusarivirus 4 (BcFV4)	BCI12	BCI12_TRINITY_DN9205_c0_g1_i1	8,349	1,657	187.72	0.0	MK558256	R. solani fusarivirus 2	36.86
				HP					692	77.59	0.0			44.76
*Fusarivirirus*	MN617764	RNA	ssRNA(+)	RdRp	Botrytis cinerea fusarivirus 5 (BcFV5)	BCS3	BCS3_TRINITY_DN2128_c0_g1_i1	6,313	1,542	173.42	0.0	MK279504	R. firma fusarivirus 1	51.02
				HP					494	55.55	1.00E–13			26.14
*Fusarivirirus*	MN617765	RNA	ssRNA(+)	RdRp	Botrytis cinerea fusarivirus 6 (BcFV6)	BCS8	BCS8_TRINITY_DN9299_c0_g1_i1	6,301	1,542	173.52	0.0	MK279504	R. firma fusarivirus 1	51.58
				HP					491	55.22	2.00E–16			23.66
*Fusarivirirus*	MN617766	RNA	ssRNA(+)	RdRp	Botrytis cinerea fusarivirus 7 (BcFV7)	BCS13	BCS13_Contig13	7,881	1,675	193.08	0.0	KP842791	S. sclerotiorum fusarivirus 1	68.25
				HP					601	69.03	0.0			55.15
*Hypovirus*	MN617169	RNA	ssRNA(+)	Polyprotein	Botrytis cinerea hypovirus 2 (BcHV2)	BCS3	BCS3_TRINITY_DN9124_c0_g1_i1	13,722	4,199	479.4	0.0	KP900893	M. phaseolina hypovirus 1	42.44
*Hypovirus*	MN617170	RNA	ssRNA(+)	Polyprotein	Botrytis cinerea hypovirus 3 (BcHV3)	BCI1	BCI1_Contig3	10,863	3,042	347.55	0.0	MF444220	S. sclerotiorum hypovirus 1-A	94.18
*Hypovirus*	MN617171	RNA	ssRNA(+)	ORF2	Botrytis cinerea hypovirus 4 (BcHV4)	BCS17	BCS17_TRINITY_DN134_c0_g2_i1	17,631	3,345	380.34	0.0	MK558259	R. solani hypovirus 1	37.00
				ORF1					1,023	113.62	0.0	KJ561218	S. sclerotiorum hypovirus 2	58.30
*Hypovirus*	MT157414	RNA	ssRNA(+)	RdRp	Botrytis cinerea hypovirus 5 (BcHV5)	BCI10	BCI10_TRINITY_DN5057_c0_g1_i1	15,353	4,856	547.55	0.0	MH347276	S. sclerotiorum hypovirus 2	93.33
*Hypovirus*	MH347277	RNA	ssRNA(+)	Polyprotein	Botrytis cinerea hypovirus 1 (BcHV1)	BCS11	BCS11_Contig10	10,483	2,965	336.61	0.0	MH347277	B. cinerea hypovirus 1	98.25
*Hypovirus*	MG554634	RNA	ssRNA(+)	HP	Botrytis cinerea hypovirus 1 satellite-like RNA	BCS12	BCS12_Contig3	4,366	670	74.04	0.0	MG554634	B. cinerea hypovirus 1 satellite like RNA	97.91
*Hypovirus*	MF444221	RNA	ssRNA(+)	HP	Sclerotinia sclerotiorum hypovirus 1 A (SsHV1A)	BCI8	BCI8_Contig1	4,578	647	71.64	0.0	MF444221	S. sclerotiorum hypovirus 1 A	94.91
*Narnavirus*	MN619795	RNA 1	ssRNA(+)	RdRp	Botrytis cinerea binarnavirus 1 (BcBNV1)	BCI12	BCI12_TRINITY_DN4441_c0_g2_i1	2,572	825	92.66	0.0	MK584836	A. tenuissima binarnavirus 1	47.16
	MT711186	RNA2		HP			BCI12_TRINITY_DN4441_c0_g1_i1	2,288	716	79.99			B. cinerea narnavirus 2 RNA2	49.82
*Narnavirus*	MN619796	RNA 1	ssRNA(+)	RdRp	Botrytis cinerea binarnavirus 2 (BcBNV2)	BCS14	BCS14_TRINITY_DN413_c0_g1_i1	2,554	813	90.85	0.0	MK584836	A. tenuissima narnavirus 1	54.98
	MT119676	RNA 2		HP			BCS14_TRINITY_DN413_c0_g2_i1	2,289	731	82.58	0.0001	MF176348	Wilkie narna-like virus 2	27.56
*Narnavirus*	MN619797	RNA 1	ssRNA(+)	RdRp	Botrytis cinerea binarnavirus 3 (BcBNV3)	BCS8	BCS8_TRINITY_DN5048_c1_g1_i1	2,553	823	92.44	0.0	MK584836	A. tenuissima narnavirus 1	46.81
	MT711185	RNA 2		HP			BCS8_TRINITY_DN10205_c0_g1	2,282	712	80.03			B. cinerea binarnavirus 2 RNA2	50.29
*Narnavirus*	MN619798	RNA	ssRNA(+)	RdRp	Botrytis cinerea narnavirus 4 (BcNV4)	BCS1	BCS1_TRINITY_DN5229_c0_g1_i1	2,471	739	84.59	2E−22	LC150604	F. poae narnavirus 1	26.03
*Narnavirus*	MN619799	RNA 1	ssRNA(+)	RdRp	Botrytis cinerea binarnavirus 5 (BcBNV5)	BCS8	BCS8_TRINITY_DN967_c0_g1_i1	2,411	754	85.58	0.0	MK584835	C. tenuissimum narnavirus 1	63.01
	MT711187	RNA 2		HP			BCS8_TRINITY_DN9735_c0_g1_i1	2,253	707	80.18			B. cinerea binarnavirus 2 RNA2	47.28
*Botoulivirus*	MT119674	RNA	ssRNA(+)	RdRp	Botrytis ourmia-like virus (BOLV)	BCS15	BCS15_TRINITY_DN11430_c0_g1_i1	2,885	722	82.04	0.0	LN827956	Botrytis ourmia-like virus	96.26
*Botourmiaviridae*	MN605467	RNA	ssRNA(+)	RdRp	Botrytis cinerea ourmia-like virus 1 (BcOLV1)	BCS12	BCS12_TRINITY_DN83_c0_g1_i10	5,185	954	107.84	1.00E−109	MK584843	P. minimum ourmia-like virus 1	41.36
*Botourmiaviridae*	MN605468	RNA	ssRNA(+)	RdRp	Botrytis cinerea ourmia-like virus 2 (BcOLV2)	BCS12	BCS12_Contig6	3,561	909	102.89	1.00E−120	MK584843	P. minimum ourmia-like virus 1	45.07
*Botourmiaviridae*	MN605469	RNA	ssRNA(+)	RdRp	Botrytis cinerea ourmia-like virus 3 (BcOLV3)	BCS8	BCS8_TRINITY_DN11_c0_g1_i2	3,286	946	107.05	3E–120	MN532673	P. minimum ourmia-like virus 1	43.85
*Botourmiaviridae*	MN605470	RNA	ssRNA(+)	RdRp	Botrytis cinerea ourmia-like virus 4 (BcOLV4)	BCI10	BCI10_TRINITY_DN4356_c0_g1_i1	2,861	735	83.18	0.0	LN827955	Botrytis ourmia-like virus	67.27
*Botourmiaviridae*	MN605471	RNA	ssRNA(+)	RdRp	Botrytis cinerea ourmia-like virus 5 (BcOLV5)	BCI5	BCI5_TRINITY_DN19_c0_g1_i1	3,376	738	83.83	9,00E-42	MK584840	C. uredinicola ourmiavirus 1	33.15
*Botourmiaviridae*	MN605472	RNA	ssRNA(+)	RdRp	Botrytis cinerea ourmia-like virus 6 (BcOLV6)	BCS1	BCS1_TRINITY_DN27_c0_g1_i1	2,697	692	78.81	0.0	MK584845	A. sclerotigenum ourmia-like virus 1	51.83
*Botourmiaviridae*	MN605473	RNA	ssRNA(+)	RdRp	Botrytis cinerea ourmia-like virus 7 (BcOLV7)	BCS14	BCS14_TRINITY_DN363_c0_g1_i1	2,100	515	59.09	3.00E–162	MK584845	A. sclerotigenum ourmia-like virus 1	51.20
*Botourmiaviridae*	MN605474	RNA	ssRNA(+)	RdRp	Botrytis cinerea ourmia-like virus 8 (BcOLV8)	BCS3	BCS3_TRINITY_DN27_c0_g2_i1	2,706	692	78.7	0.0	MK584845	A. sclerotigenum ourmia-like virus 1	51.63
*Botourmiaviridae*	MN605475	RNA	ssRNA(+)	RdRp	Botrytis cinerea ourmia-like virus 9 (BcOLV9)	BCI10	BCI10_TRINITY_DN8574_c0_g1_i1	2,941	804	90.92	0.0	MK584839	P. sumatrense ourmia-like virus 1	49.92
*Botourmiaviridae*	MN605476	RNA	ssRNA(+)	RdRp	Botrytis cinerea ourmia-like virus 10 (BcOLV10)	BCS1	BCS1_TRINITY_DN3465_c0_g1_i1	2,430	615	70.15	2.00E–126	LC413502	P. oryzae ourmia-like virus 2	62.20
*Botourmiaviridae*	MN605477	RNA	ssRNA(+)	RdRp	Botrytis cinerea ourmia-like virus 11 (BcOLV11)	BCI7	BCI7_TRINITY_DN2190_c0_g1_i1	2,850	671	76.18	0.0	KP900929	S. sclerotiorum ourmia-like virus 2	53.92
*Botourmiaviridae*	MN605478	RNA	ssRNA(+)	RdRp	Botrytis cinerea ourmia-like virus 12 (BcOLV12)	BCI10	BCI10_TRINITY_DN3618_c0_g1_i1	2,898	688	78.81	2E–159	KP900929	S. sclerotiorum ourmia-like virus 2	44.53
*Botourmiaviridae*	MN605479	RNA	ssRNA(+)	RdRp	Botrytis cinerea ourmia-like virus 13 (BcOLV13)	BCS2	BCS2_Contig15	2,877	710	81.33	1E–132	KP900929	S. sclerotiorum ourmia-like virus 2	43.09
*Botourmiaviridae*	MN605480	RNA	ssRNA(+)	RdRp	Botrytis cinerea ourmia-like virus 14 (BcOLV14)	BCS15	BCS15_TRINITY_DN11704_c0_g1_i1	2,484	635	73.25	2.00E–128	MK584842	E. nigrum ourmia-like virus 1	40.29
*Botourmiaviridae*	MN605481	RNA	ssRNA(+)	RdRp	Botrytis cinerea ourmia-like virus 15 (BcOLV15)	BCS2	BCS2_TRINITY_DN470_c0_g2_i2	2,428	645	73.9	1.00E–128	MK584842	E. nigrum ourmia-like virus 1	39.94
*Botourmiaviridae*	MN605482	RNA	ssRNA(+)	RdRp	Botrytis cinerea ourmia-like virus 16 (BcOLV16)	BCS12	BCS12_TRINITY_DN3_c0_g1_i4	2,493	650	73.52	2.00E–126	KP900929	S. sclerotiorum ourmia-like virus 2	39.87
*Botourmiaviridae*	MN605483	RNA	ssRNA(+)	RdRp	Botrytis cinerea ourmia-like virus 17 (BcOLV17)	BCI2	BCI2_TRINITY_DN5291_c0_g1_i1	2,837	663	74.78	2.00E–132	MK584842	E. nigrum ourmia-like virus 1	40.69
*Botourmiaviridae*	MT119675	RNA	ssRNA(+)	RdRp	P. oryzae ourmia-like virus 2 (PoOLV2)	BCI6	BCI6_TRINITY_DN4570_c0_g1_i1	1,320	366	41.17	0.0	LC413502	P. oryzae ourmia like virus 2	93.99
*Mitovirus*	MT119677	RNA	ssRNA(+)	RdRp	Botrytis cinerea mitovirus 1 (BcMV1)	BCS1	BCS1_TRINITY_DN4879_c0_g1_i1	2,788	738	83.74	0.00	LN827940	B. cinerea mitovirus 1	97.02
*Mitovirus*	MN617165	RNA	ssRNA(+)	RdRp	Botrytis cinerea mitovirus 2 (BcMV2)	BCS8	BCS8_Contig13	2,502	710	82.38	0.0	LN827945	B. cinerea mitovirus 2	96.76
*Mitovirus*	MN617166	RNA	ssRNA(+)	RdRp	Botrytis cinerea mitovirus 3 (BcMV3)	BCS16	BCS16_Contig12_len = 2977	2,977	786	89.27	0.0	LN827942	B. cinerea mitovirus 3	94.53
*Mitovirus*	MN954875	RNA	ssRNA(+)	RdRp	Botrytis cinerea mitovirus 4 (BcMV4)	BCS16	BCS16_Contig23	2,709	731	85.61	0.0	LN827947	B. cinerea mitovirus 4	93.71
*Mitovirus*	MN617167	RNA	ssRNA(+)	RdRp	Botrytis cinerea mitovirus 5 (BcMV5)	BCS17	BCS17_TRINITY_DN132_c0_g1_i8	2,721	731	85.51	0.00	LN827947	B. cinerea mitovirus 4	88.24
*Mitovirus*	MN625252	RNA	ssRNA(+)	RdRp	Botrytis cinerea mitovirus 6 (BcMV6)	BCI1	BCI1_TRINITY_DN10328_c0_g1_i1	2,519	710	80.87	0.00	MN035976	Mitovirus sp.	48.19
*Mitovirus*	MN617168	RNA	ssRNA(+)	RdRp	Botrytis cinerea mitovirus 7 (BcMV7)	BCI5	BCI5_TRINITY_DN3722_c0_g1_i1	2,705	731	85.46	0.00	KT365895	Sclerotinia nivalis mitovirus 1	86.32
*Mitovirus*	MN625253	RNA	ssRNA(+)	RdRp	Botrytis cinerea mitovirus 8 (BcMV8)	BCS17	BCS17_TRINITY_DN76_c0_g1_i1	2,362	713	83.07	0.00	MN033004	Mitovirus sp.	50.34
*Mitovirus*	MN954876	RNA	ssRNA(+)	RdRp	Sclerotinia sclerotiorum mitovirus 3 (SsMV3)	BCI2	BCI2_TRINITY_DN28_c0_g1_i4	2,974	607	68.52	0.0	LN827949	S. sclerotiorum mitovirus 3	96.66
*Mitovirus*	MN954877	RNA	ssRNA(+)	RdRp	Sclerotinia sclerotiorum mitovirus 4 (SsMV4)	BCS16	BCS16_Contig24	2,737	731	85.67	0.0	JX401538	S. sclerotiorum mitovirus 4	96.22
*Mitovirus*	MT089704	RNA	ssRNA(+)	RdRp	Botrytis cinerea mitovirus 9 (BcMV9)	BCS4	BCS4_TRINITY_DN9446_c0_g1_i1	2,720	719	82.74	0.0	LN827948	Grapevine-associated narnavirus 1	96.38
*Mycoflexivirus*	LN827953	RNA	ssRNA(+)	RdRp	*Botrytis* virus F (BVF)	BCI10	BCI10_Contig9	7,004	2,002	224.31	0.0	LN827953	Botrytis virus F	98.10
				CP					332	8.3	1.00E–157	LN827949		97.25
*Tymoviridae*	MN954873	RNA	ssRNA(+)	RdRp	Botrytis cinerea mycotymovirus 1 (BcMTV1)	BCS15	BCS15_TRINITY_DN310_c0_g1_i1	7,293	2,347	261.25	0.0	MF444267	S. sclerotiorum mycotymovirus 1	94.90
*Phenuiviridae*	MN617081	RNA1	ssRNA(–)	RdRp	Botrytis cinerea bocivirus 1 (BcBV1)	BCS15	BCS15_Contig41	6,729	2,211	253.98	0.0	KY781184	Watermelon crinkle leaf-associated virus 1	39.58
	MN617080	RNA2		ORF2			BCS15_TRINITY_DN307_c0_g1_i1	1,646	470	52.79	1E–148	MG256515	Laurel Lake virus	51.30
	MN617079	RNA3		CP			BCS15_TRINITY_DN360_c0_g1_i1	1,284	356	39.21	7E−55	MK689373	Citrus virus A	35.26
ssRNA negative-strand virus	MN617149	RNA	ssRNA(–)	RdRp	Botrytis cinerea negative-stranded RNA virus 2 (BcNSRV2)	BCS16	BCS16_TRINITY_DN9775_c0_g3_i1	6,510	2,135	245.02	0.0	MK584854	Coniothyrium diplodiella negative-stranded RNA virus 1	52.77
*Mymonaviridae*	MN617150	RNA	ssRNA(–)	RdRp (gp5)	Botrytis cinerea negative-stranded RNA virus 3 (BcNSRV3)	BCS11	BCS11_Contig19	9,875	1,934	221.11	0.0	KC601997	S. sclerotiorum negative-stranded RNA virus 3	56.07
				gp2					410	45.65	4.00E–111			46.02
				HP (=gp6)					220	25.35	4.00E−05	KJ186782	S. sclerotiorum negative-stranded RNA virus 1	30.83
				gp1					285	31.71	1.00E–13	KC601997	S. sclerotiorum negative-stranded RNA virus 3	25.80
*Mymonaviridae*	MN617151	RNA	ssRNA(–)	RdRp	Botrytis cinerea negative-stranded RNA virus 4 (BcNSRV4)	BCS12	BCS12_TRINITY_DN161_c0_g1_i1	9,211	1,949	223.88	0.0	MF444280	S. sclerotiorum negative-stranded RNA virus 3-A	50.05
				HP					180	20.42	1.00E−05	KJ186782	S. sclerotiorum negative-stranded RNA virus 1	31.58
				gp2					385	43.28	7.00E−108	KC601997	S. sclerotiorum negative-stranded RNA virus 3	43.86
				HP2					245	27.23			No significant similarity found	
*Mymonaviridae*	MN617152	RNA	ssRNA(–)	RdRp	Botrytis cinerea negative-stranded RNA virus 5 (BcNSRV5)	BCS15	BCS15_TRINITY_DN100_c0_g1_i1	9,817	1,953	221.91	0.0	MF444283	S. sclerotiorum negative-stranded RNA virus 5	81.13
				HP1				284	284	31.73			No significant similarity found	
				HP2				394	394	43.71			No significant similarity found	
				HP3				334	334	37.51			No significant similarity found	
*Bunyavirales*	MN617082	RNA	ssRNA(–)	RdRp	Botrytis cinerea negative-stranded RNA virus 6 (BcNSRV6)	BCI11	BCI11_TRINITY_DN2250_c0_g1_i1	7,071	2,258	266.3	0.0	MK507779	R. solani bunya/phlebo-like virus 1	37.69
*Mymonaviridae*	MN954878	RNA	ssRNA(–)	RdRp	Botrytis cinerea negative-stranded RNA virus 7 (BcNSRV7)	BCI2	BCI2_Contig7	10,352	1,953	223.07	0.0	MF444283	S. sclerotiorum negative-stranded RNA virus 5	99.14
				HP1					300	33.92			No significant similarity found	
				HP2					394	43.82			No significant similarity found	
				HP3					318	36.19			No significant similarity found	
*Mymonaviridae*	MT157412	RNA	ssRNA(–)	RdRp	Botrytis cinerea negative-stranded RNA virus 7 isolate BCI7	BCI7	BCI7_Contig4	10,153	1,953	222.43		MF444283	S. sclerotiorum negative-stranded RNA virus 5	99.14
				HP1					300	33.78	0.0		No significant similarity found	
				HP2					394	43.78			No significant similarity found	
				HP3					321	36.66			No significant similarity found	
*Mymonaviridae*	MT157413	RNA	ssRNA(–)	RdRp	Botrytis cinerea negative-stranded RNA virus 7 isolate BCS13	BCS13	BCS13_TRINITY_DN1_c0_g1_i1	10,002	1,953	222.23	0.0	MF444283	S. sclerotiorum negative-stranded RNA virus 5	99.15
				HP1					300	33.69			No significant similarity found	
				HP2					394	43.82			No significant similarity found	
				HP3					321	36.51			No significant similarity found	
ssRNA negative-strand virus	MT157408	RNA	ssRNA(–)	RdRp	Botrytis cinerea negative-stranded RNA virus 8 (BcNSRV8)	BCS16	BCS16_TRINITY_DN284_c0_g3_i1	10,220	3,385	395.55	0.0	LN827956	B. cinerea negative-stranded RNA virus 1	96.82
ssRNA negative-strand virus	MT157409	RNA	ssRNA(–)	RdRp	Botrytis cinerea negative-stranded RNA virus 9 (BcNSRV9)	BCS8	BCS8_TRINITY_DN5169_c0_g1_i1	10,287	3,365	394.23	0.0	LN827956	B. cinerea negative-stranded RNA virus 1	68.87
ssRNA negative-strand virus	MT157410	RNA	ssRNA(–)	RdRp	Botrytis cinerea negative-stranded RNA virus 10 (BcNSRV10)	BCS9	BCS9_TRINITY_DN4026_c0_g1_i1	10,312	3,408	399.04	0.0	LN827956	B. cinerea negative-stranded RNA virus 1	68.98
ssRNA negative-strand virus	MT157411	RNA	ssRNA(–)	RdRp	Botrytis cinerea negative-stranded RNA virus 11 (BcNSRV11)	BCS17	BCS17_TRINITY_DN1732_c0_g3_i1	10,168	3,339	389.93	0.0	LN827956	B. cinerea negative-stranded RNA virus 1	98.34
ssRNA negative-strand virus	MN617078	RNA	ssRNA(–)	RdRp	Botrytis cinerea orthobunya-like virus 1 (BcOBV1)	BCI9	BCI9_TRINITY_DN4209_c0_g1_i1	8,058	2,646	313.64	2E−12	JN968590	C. porteira virus	23.98
ssRNA negative-strand virus	MT157407	RNA	ssRNA(–)	RdRp	Botrytis cinerea orthobunya-like virus 1 isolate BCI5	BCI5	BCI5_TRINITY_DN26_c0_g1_i1	8,027	2,646	313.34	2.00E−12	JN968590	C. porteira orthobunyavirus	24.25
*Genomoviridae*	MN625247	DNA	ssDNA	Rep	Botrytis cinerea ssDNA virus 1 (BcssDV1)	BCS11	BCS11_TRINITY_DN160_c0_g1_i2	1,694	321	36.62	8.00E–68	KY230625	Bemisia-associated genomovirus NfO	42.86
				sRep					380	43.32	5.00E−72	KY230625	Bemisia-associated genomovirus NfO	42.86

a ORF, open reading frame; RdRp, RNA-dependent RNA polymerase; HP, hypothetical protein; CP, coat protein; RaP, replication-associated protein; Rep, replication protein; sRep, spliced replication-associated protein.

b “ssRNA(+)” and “ssRNA(+)” indicate positive- and negative-strand single-stranded RNAs, respectively.

### Geographical distribution of *B. cinerea* mycoviruses.

The distribution and prevalence of *B. cinerea* mycoviruses in the different Italian and Spanish regions (see Fig. S1 in the supplemental material) were examined considering the existence of more samples and pools from Spain than from Italy. Mycovirus classification was performed by manual inspection and allowed to elucidate different taxonomical groups. Among the 92 mycoviruses found, 19 were classified as double-stranded RNA (dsRNA) mycoviruses, 15 as negative-sense single-stranded RNA (ssRNA–) mycoviruses, 57 as positive-sense single-stranded RNA (ssRNA+) mycoviruses, and 1 as a single-stranded DNA (ssDNA) mycovirus. For these analyses, only the sequence of the segment containing the RNA-dependent RNA polymerase (RdRp) was used, with the exception of the sequence of Botrytis cinerea hypovirus 1 satellite like RNA (associated with its auxiliary mycovirus, Botrytis cinerea hypovirus 1) that was also included (see Fig. S2). Distribution of the distinct types of mycoviruses in the different regions inside each country was studied (see Fig. S2A) considering that there were well-represented regions (the Spanish regions of La Rioja and Ribera del Duero and the Italian region of Piemonte), moderately represented regions (Jerez and Penedés in Spain and Veneto and Lombardia in Italy), and poorly represented regions (the Italian regions of Basilicata and Sicilia) (see Fig. S1). The ssDNA mycovirus was present in the Italian region of Lombardia and in three of the four Spanish regions, with the exception of the southern region of Jerez. Mycoviruses with the ssRNA+ genome, the most abundant in this study, were equally distributed in both countries, with higher prevalences in the northern Spanish region of La Rioja and lower prevalences in the southern Italian region of Sicilia. Mycoviruses with the dsRNA or ssRNA– genome were more frequently found in Spain than in Italy. dsRNA mycoviruses in Italy were more prevalent in Veneto and not present in Lombardia; however, in Spain they were present in all regions but with less prevalence in the southern region of Jerez. Mycoviruses with the ssRNA– genome were not present in the southern regions of Spain and Italy, Jerez and Sicilia, respectively, and were equally distributed in the remaining Italian regions, and more prevalent in La Rioja and Penedés than in Ribera del Duero. Although Basilicata and Sicilia were the most separated regions in Italy and less represented, with three and four samples (see Fig. S1), respectively, both had isolates of *B. cinerea* infected with all types of mycoviruses, with the exception of the ssDNA mycovirus.

10.1128/mBio.03705-20.1 FIG S1 Sampling sites and regions of *B. cinerea* in Spain (A) and Italy (B). Each sample location is indicated on the map. Pools were sequenced by high-throughput sequencing with the number of samples included in each, with its location inside the different regions and the total number of samples. Spanish pools (*n* = 150); Italian pools (*n* = 98). Download FIG S1, PPTX file, 0.3 MB. Copyright © 2021 Ruiz-Padilla et al. 2021 Ruiz-Padilla et al. https://creativecommons.org/licenses/by/4.0/ This content is distributed under the terms of the Creative Commons Attribution 4.0 International license.

10.1128/mBio.03705-20.2 FIG S2 Distribution of mycoviruses in Italy and Spain. (A) Distribution in the different regions inside each country. (B) Distribution in the different pools inside each region. (C) Mycoviruses common or exclusive to each country. Download FIG S2, TIF file, 2.5 MB. Copyright © 2021 Ruiz-Padilla et al. 2021 Ruiz-Padilla et al. https://creativecommons.org/licenses/by/4.0/ This content is distributed under the terms of the Creative Commons Attribution 4.0 International license.

The presence of the different types of mycoviruses in the individual pools (represented by more than 500 reads; see Fig. S2B), most of each were represented by samples of the same site inside each region (see Fig. S1), was also explored. ssDNA mycovirus is not represented in the graphic but was present in both pools from Lombardia, in one pool from Ribera de Duero, in three pools from Penedés, and in four pools from La Rioja (see Table S1 in the supplemental material). In Sicilia and Jerez, all pools contained both types of mycoviruses, i.e., dsRNA and ssRNA+ mycoviruses. In the remaining regions that contained the three types of mycoviruses, all pools had at least two types of mycoviruses, with the most abundant ssRNA+ mycoviruses being present in all pools (see Fig. S2B). In this graphic, the number of mycoviruses per pool is also shown (considering only mycoviruses represented by more than 500 reads; see Table S1). There were pools with the same number of samples, for instance, 10, containing only 6 (dsRNA and ssRNA+) mycoviruses, such as BCI6, and pools containing 42 (dsRNA, ssDNA, ssRNA+, and ssRNA–) mycoviruses, such as BCS15, or pools, such as BSC13, with a mix of 4 *B. cinerea* samples and 39 mycoviruses. In addition, the distribution of all mycoviruses in both countries was analyzed. Of the 92 mycoviruses, 5 (including ssRNA+ and ssRNA– mycoviruses) were exclusively present in Italy, 32 were found only in Spain, and 55 were common between both countries (see Fig. S2C).

10.1128/mBio.03705-20.9 TABLE S1 Mycoviruses found in this study and its distribution in the different pools are indicated by the number of reads mapping to each sequence. The number of reads from 100 to 500 is labeled in light blue, and the number of reads >500 is labeled in dark blue. Download Table S1, XLSX file, 0.03 MB. Copyright © 2021 Ruiz-Padilla et al. 2021 Ruiz-Padilla et al. https://creativecommons.org/licenses/by/4.0/ This content is distributed under the terms of the Creative Commons Attribution 4.0 International license.

### Characterization of novel mycoviruses.

**FIG 1:**
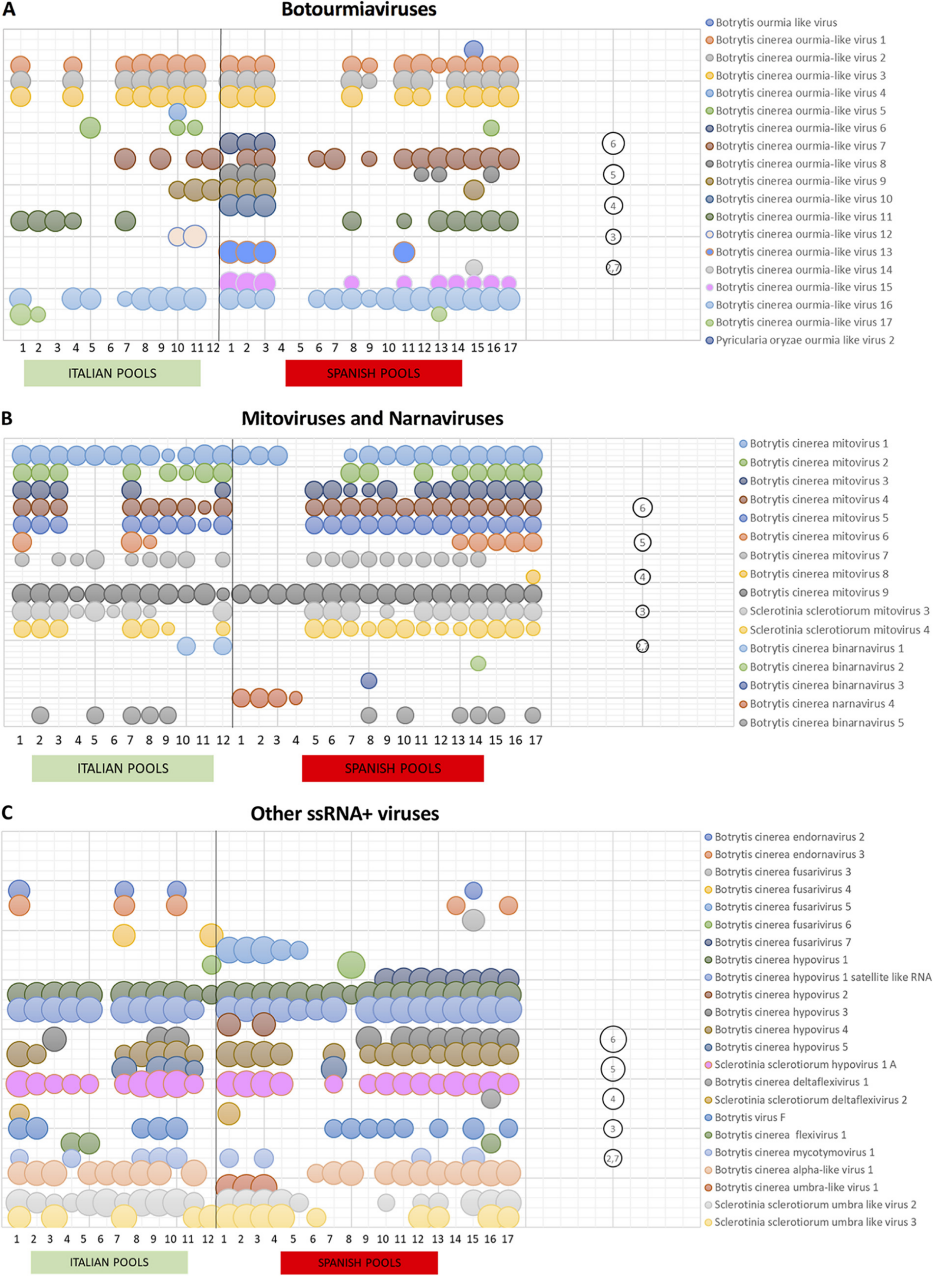
Distribution and abundance of *Botrytis cinerea* ssRNA+ mycoviruses within regions. Bubble charts based on the number of reads, with log_10_ transformation, per each mycovirus inside the pools are shown. A bubble of “2,7” represents 500 reads. (A) Botourmiaviruses. (B) Mitoviruses and narnaviruses. (C) Other ssRNA+ mycoviruses.

**FIG 2:**
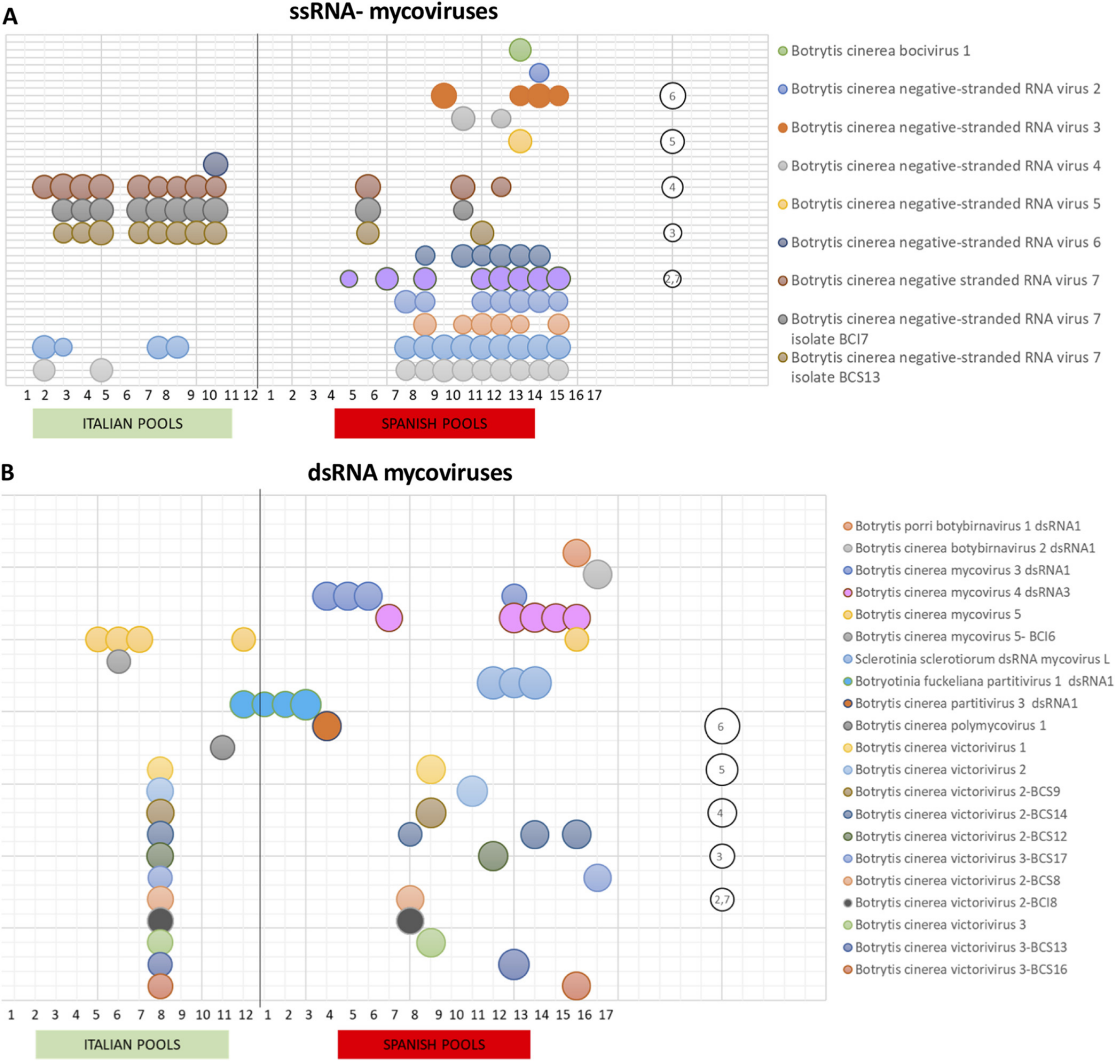
Distribution and abundance of *Botrytis cinerea* ssRNA– and dsRNA mycoviruses within regions. Bubble charts based on the number of reads, with log_10_ transformation, per each mycovirus inside the pools are shown. A bubble of “2,7” represents 500 reads. (A) ssRNA– mycoviruses. (B) dsRNA mycoviruses.

Here, we have divided the description of novel mycoviruses based on the type of genome: ssRNA–, ssRNA+, dsRNA, and ssDNA. The distribution of all mycoviruses, based on the number of reads mapping to each sequence, is shown in Table S1. The presence of each mycovirus per pool, considering only number of reads above 500, is indicated in Fig. 1 and 2 in five different graphics based on their genome types. The accession numbers of each mycovirus reported in the present study are indicated and highlighted in the phylogenetic trees and in Table 1.

### Positive single-stranded RNA mycoviruses.

A total of 57 mycoviruses with ssRNA+ genomes were identified here. Of these, 35 were classified as mitoviruses (*n* = 11), narnaviruses (*n* = 5), and ourmia-like viruses (*n* = 19). The other 22 ssRNA+ mycoviruses were classified as tymovirales (*n* = 1), umbraviruses (*n* = 3), fusariviruses (*n* = 5), hypoviruses (*n* = 6), endornaviruses (*n* = 2), deltaflexiviruses (*n* = 2), mycoflexiviruses (*n* = 1), flexiviruses (*n* = 1), and alphaviruses (*n* = 1).

### (i) Botourmiavirus, mitovirus, and narnavirus.

The majority of the ssRNA+ mycoviruses found in our study belong to the phylum *Lenarviricota* that now includes the families *Leviviridae*, *Narnaviridae*, *Mitoviridae*, and *Botourmiaviridae* (46). Fungal botourmiaviruses are nonencapsidated monosegmented ssRNA+ viruses encoding an RdRp (47). New botourmiaviruses characterized in this study were named Botrytis cinerea ourmia-like viruses (BcOLVs) 1 to 17. In addition to these viruses, we identified two variants of the already-described Botrytis ourmia-like virus (BOLV) (3) and Pyricularia oryzae ourmia like virus 2 (PoOLV2) (17) (Table 1). Both mycoviruses were annotated as variants of the *B. cinerea* isolates from grapevine. BOLV-BCS15 was annotated as a variant of BOLV (MT119674, with an almost complete sequence of 2,885 nucleotides [nt] of 2,903 nt and with identities of 95 and 96% at the nucleotide and amino acid [aa] levels, respectively), and PoOLV2-BCI16 was annotated as a variant of PoOLV2 (MT119675), since the sequence found in this study was partial (1,320 nt of 1,671 nt, with identities of 91 and 94% at nucleotide and amino acid levels, respectively). The 17 new botourmiaviruses have variable lengths between 2,100 and 5,185 nt and, independent of the size, they all encode a single protein of 515 to 954 aa containing amino acids conserved inside the domains of the viral RdRps of ssRNA+ viruses, including the highly conserved core domain GDD (motif VI) (see Fig. S3A and C), which indicates that these proteins are putative mycoviral RdRps. The highest identity at the amino acid level (90.59%) was found between BcOLV6 and BcOLV8 (see Fig. S3B; the identity at nucleotide level was 89.24%). The remaining botourmiaviruses showed an identity at the amino acid level of <84.55% (see Fig. S3B). The closest mycoviruses of the identified *B. cinerea* botourmiaviruses (% identity of the RdRp sequence) are shown in Table 1. The number of reads of botourmiaviruses is high in most of the pools where they are present (see Table S1), even though not all of them are equally distributed. BcOLV1, -2, -3, and -16 were very abundant in Italian and Spanish pools; however, BOLV, BcOLV4, and BcOLV14 were present in a single pool with low numbers of reads, with the remaining mycoviruses having different representations in at least more than two pools (Fig. 1A).

10.1128/mBio.03705-20.3 FIG S3 Sequences properties of botourmiaviruses and mitoviruses. The percent identity matrix was generated by using Clustal Omega 2.1. The level of identity from higher to lower is labeled in a color range from dark green to dark red, respectively. (A) Schematic representation of BcOLV RNA genomes showing location of ORFs. (B) Percent identity matrix of BcOLV1 to 17. (C) Amino acid sequence alignment of RdRps of Botrytis cinerea ourmia-like virus (BcOLV) 1 to 17 and Botrytis ourmia-like virus 1 (BOLV1). (D) Schematic representation of BcMV RNA genomes showing location of ORFs. (E) Amino acid sequence alignment of RdRps of Botrytis cinerea mitovirus (BcMV) 1 to 9 and Sclerotinia sclerotiorum mitovirus 3 (SsMV3) and SsMV4. (F) Percent identity matrix of mitovirus. Download FIG S3, TIF file, 0.4 MB. Copyright © 2021 Ruiz-Padilla et al. 2021 Ruiz-Padilla et al. https://creativecommons.org/licenses/by/4.0/ This content is distributed under the terms of the Creative Commons Attribution 4.0 International license.

The genera *Mitovirus* and *Narnavirus* belong to the families *Mitoviridae* and *Narnaviridae*, respectively, that contain viruses with a single molecule of nonencapsidated RNA of 2.3 to 5 kb, which encodes a single RdRp (46, 48). In general, mitoviruses were well represented in many pools, both in Spain and in Italy, whereas narnaviruses were scattered in some pools (Fig. 1B). Eleven mitoviruses were found: four were new mitoviruses associated with *B. cinerea* isolates (Botrytis cinerea mitoviruses 5 to 8 [BcMV5 to BcMV8]), whereas the other four were considered variants of the previously described BcMV1 to -4 (23), and another two were annotated as variants of two mitoviruses infecting the fungus *S. sclerotiorum* (SsMV3 and -4) (23, 49). Finally, the last one was reported previously as Grapevine-associated narnavirus 1 (23), and we renamed it here as Botrytis cinerea mitovirus 9. These mycoviruses have lengths between 2,362 and 2,977 nt, and all of them code for a single protein with length ranging between 710 and 786 aa (see Fig. S3D), with the exception of SsMV3, which encoded a smaller protein of 607 aa (Table 1). The alignment of the protein sequences showed that all of them have amino acid sequence domains conserved inside the RdRps of mitoviruses (A to F) (see Fig. S3E), suggesting that these are the proteins involved in mycoviral replication. The closest related *B. cinerea* mitoviruses were SsMV4, BcMV4, BcMV5, and BcMV7, all of which encoded an RdRp of 731 aa with an identity ranging between 82.22 and 90.15% (see Fig. S3F).

**FIG 3:**
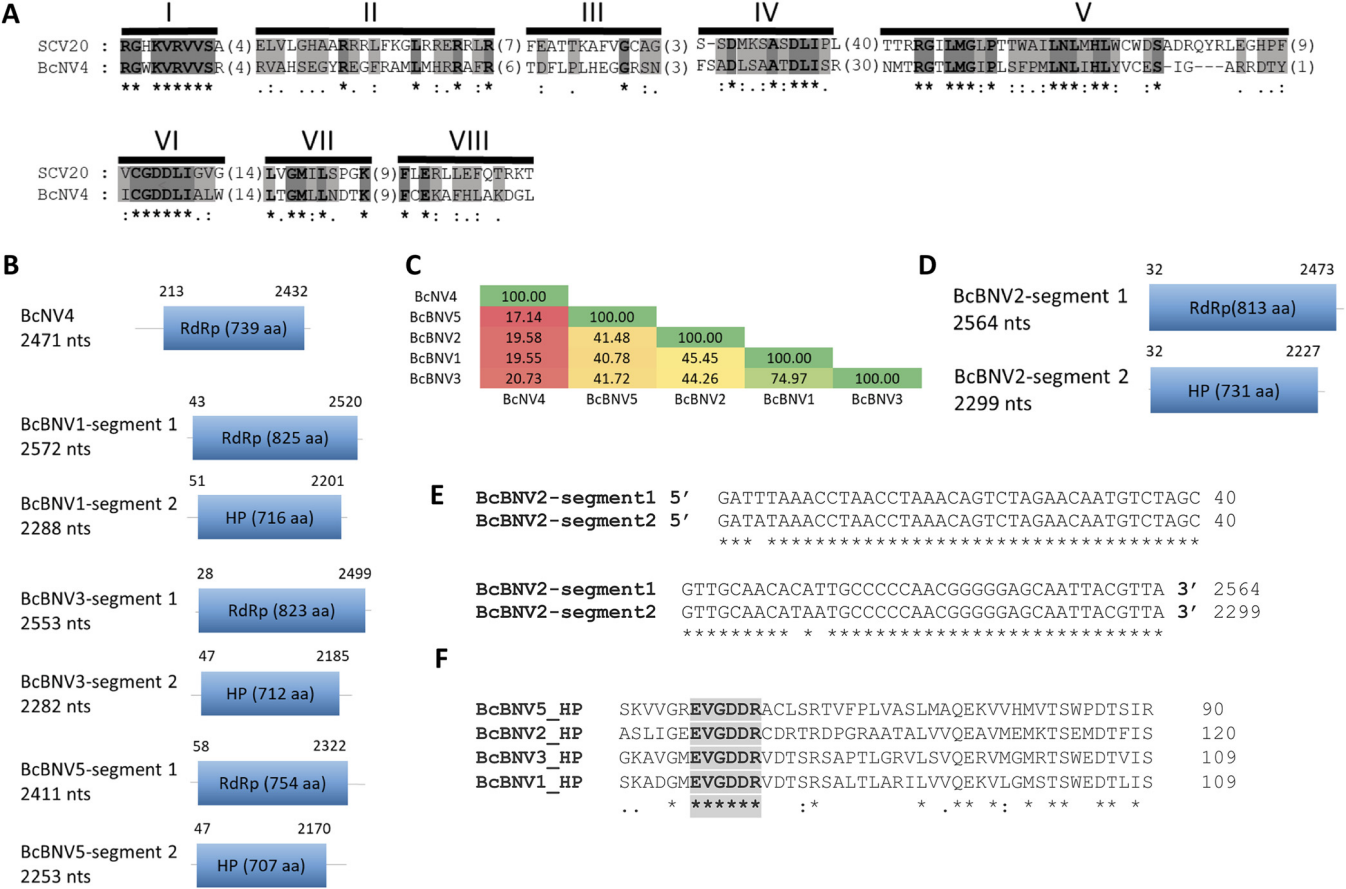
Sequence properties of *Botrytis cinerea* narna-like viruses. (A) Amino acid sequence alignment of narnavirus RdRp showing conserved motifs I to VIII of Botrytis cinerea narnavirus 4 (BcNV4) and Saccharomyces 20S RNA narnavirus (SCV20; AF039063). The conserved motifs of the RdRps are shaded with light and dark gray colors. Asterisks indicate identical amino acid residues, and colons indicate similar residues. Amino acids in parentheses show the positions of amino acid residues that are not listed. (B) Schematic representation of Botrytis cinerea binarnavirus 1 (BcBNV1), -3, -4, and -5 RNA genomes showing location of ORFs. (C) Percent identity matrix generated by Clustal Omega 2.1. Identities from higher to lower are labeled from dark green to dark red, respectively. (D) Schematic representation of BcBNV2 RNA genome showing the locations of ORFs. (E) Alignment of the conserved sequences of the 5′ and 3′ ends of BcBNV2 segments 1 and 2. (F) Alignment of the hypothetical protein sequences showing conserved stretches included a GDD triplet (EVGDDR).

Until now, there have been no reported narnaviruses infecting *B. cinerea*. Putative narna-like viral sequences were found in the analyzed samples with lengths between 2,289 nt and 2,572 nt, coding for a single protein 731 to 825 aa in size (Fig. 3B and D). The new mycoviruses were named as Botrytis cinerea narnavirus 4 (BcNV4) and Botrytis cinerea binarnavirus 1 (BcBNV1), -2, -3, and -5. The alignment of the sequence of BcNV4 protein and the sequence of Saccharomyces cerevisiae narnavirus 20S RNA RdRp (50) showed the typical conserved motifs I to VIII of the RdRp, including the core domain GDD (Fig. 3A). The putative RdRps of BcBNV1, -2, -3, and -5 are complete proteins, but the triplet GDD, which is a component of the RdRp catalytic site present in the conserved motif VI of the palm domain, is not conserved. Nevertheless, all of them show high levels of conservation in different stretches of the protein (data not shown) and have identities with the RdRp of other narnaviruses infecting other genera of fungi (Table 1). Our suggestion is that this protein (HP) could be the RdRp of these *B. cinerea* binarnaviruses. The highest identity was found between the putative RdRp of BcBNV1 and BcBNV3 (74.97%; Fig. 3C) with a very low identity values between the RdRp of BcNV4 and the rest of the binarnaviruses (17.14 to 20.73%; Fig. 3C). One of the narna-like virus sequences coded for a hypothetical protein (HP) with the first 230 nt showing certain level of identity with the putative RdRp of Wilkie narna-like virus 2 (27.56%; Table 1) (51). Surprisingly, we found that 40 nt at the 5′ and 3′ ends of this narna-like nucleotide sequence were identical to both ends in BcBNV2 (Fig. 3E). In addition, both viral sequences were present only in the Spanish pool BCS14, represented by a similar number of reads, 5,470 reads for BcBNV2 and 6,921 reads for the narna-like viral sequence (see Table S1), suggesting that both sequences could be part of a bisegmented virus, BcBNV2 segment 1 (open reading frame [ORF] RdRp) and segment 2 (ORF HP). The bisegmented nature of the BcBNV2 genome was confirmed by no amplification with combined primers of segments 1 and 2 (data not shown) and by determination of the 5′ and 3′ ends of both segments (Fig. 3E). Since BcBNV1, -2, -3, and -5 showed similar characteristics, we searched for the second segment in the pools containing BcBNV1, -3, and -5. The corresponding segments coding for a hypothetical protein were found, all with conserved sequences at the 5′ and 3′ ends with its respective segment 1 (data not shown). The identity among them was higher for the RdRp, with the lowest identity (38%) between BcBNV1 HP and BcBNV5 HP and the highest identity (71%) between BcBNV1 HP and BcBNV3 HP. The alignment of these HPs showed high levels of conservation in different stretches and, surprisingly, one of the conserved stretches included a GDD triplet (EVGDDR) (Fig. 3F). For all mentioned above, we concluded that BcBNV1, -2, -3, and -5 are bisegmented mycoviruses.

**FIG 4:**
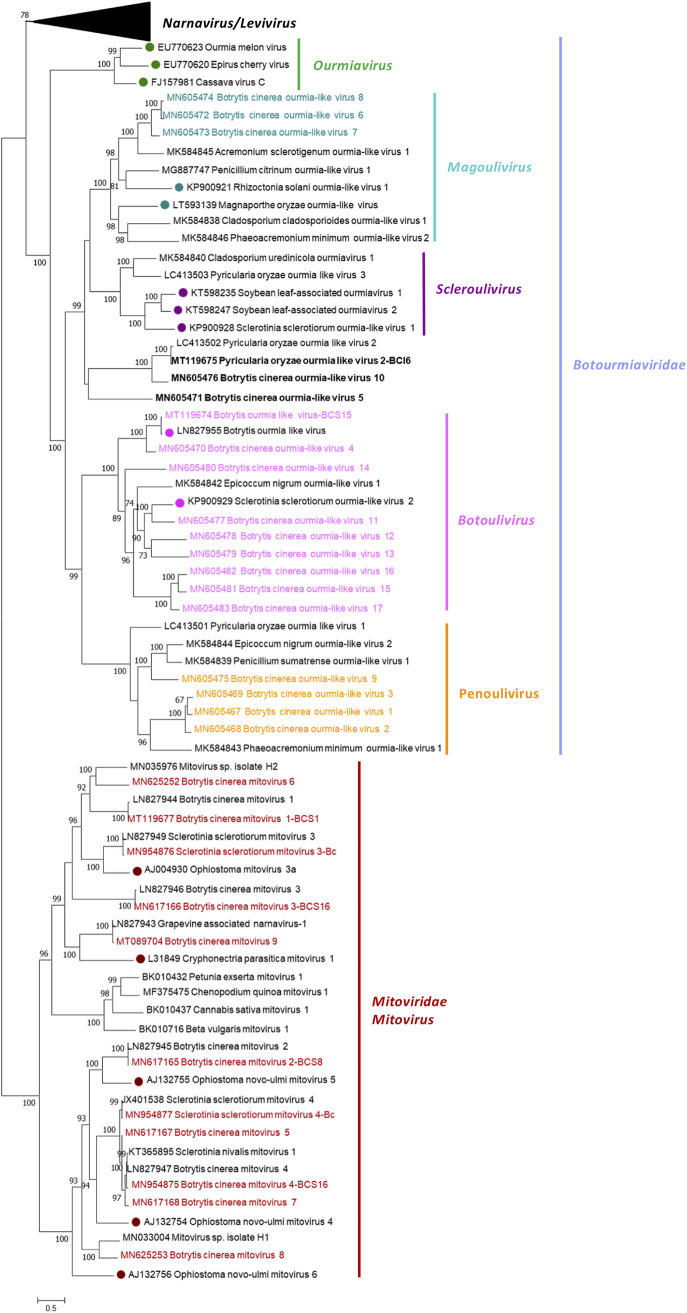
Mitovirus and botourmiavirus phylogenetic tree. A phylogenetic tree was computed by using the IQ-TREE stochastic algorithm to infer phylogenetic trees by maximum likelihood (model of substitution: VT+F+I+G4). A consensus tree was constructed from 1,000 bootstrap trees (log likelihood of consensus tree, –126059.306862). The branch of the narnaviruses and leviviruses is collapsed. All bootstrap values (%) of >65 are represented at each node of the tree. Branch lengths are proportional to the number of amino acid substitutions and are measured by a scale bar.

**FIG 5:**
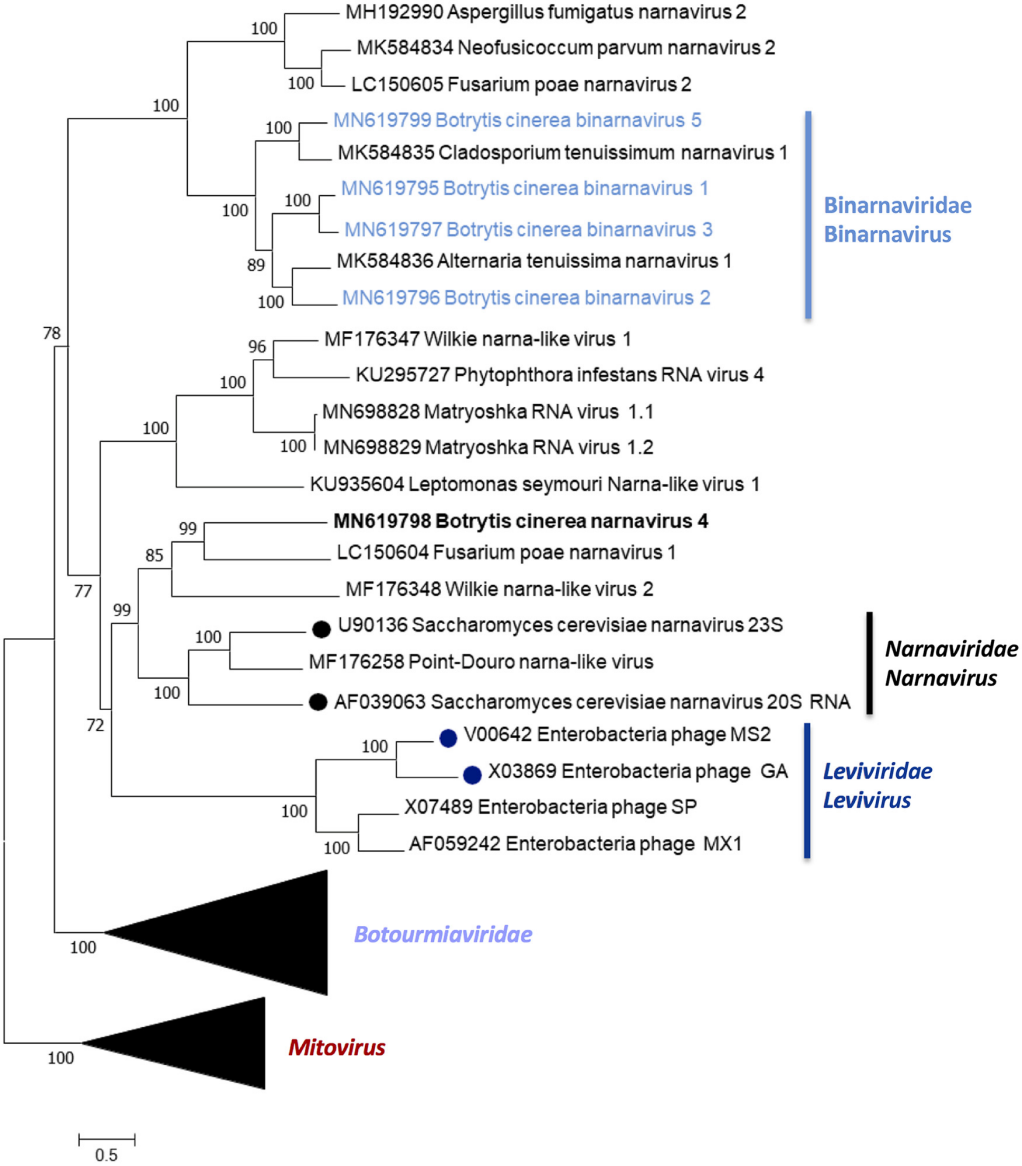
Narnavirus and levivirus phylogenetic tree. A phylogenetic tree was computed by using the IQ-TREE stochastic algorithm to infer phylogenetic trees by maximum likelihood (model of substitution: VT+F+I+G4). A consensus tree was constructed from 1,000 bootstrap trees (log likelihood of consensus tree, −126059.306862). The branch of the mitoviruses and botourmiaviruses is collapsed. All bootstrap values (%) of >65 are represented at each node of the tree. Branch lengths are proportional to the number of amino acid substitutions and are measured by a scale bar.

The phylogenetic relationships of the above-described mycoviruses are shown in Fig. 4 (*Mitoviridae* and *Botourmiaviridae*) and Fig. 5 (*Narnaviridae* and *Leviviridae*). Full-length amino acid sequences of RdRps of the new described mycoviruses and their relatives, with members of the genera *Mitovirus*, *Narnavirus*, *Levivirus*, *Botoulivirus*, *Scleroulivirus*, *Magoulivirus*, and *Ourmiavirus* (family *Botourmiaviridae*), were aligned to construct a phylogenetic tree to infer the relationships among all of them. The phylogenetic analysis showed three main clades—one including all mitoviruses, a second one including narnaviruses and leviviruses, and a third one that includes members of the family *Botourmiaviridae*. Members of the *Botourmiaviridae* family infecting fungi are separated into three different genera. Nine of the detected BcOLVs were associated with members of the genus *Botoulivirus*, and two identified BcOLVs were closely related to members of the genera *Scleroulivirus* and *Magoulivirus*. However, BcOLV1, -2, -3, and -9 are grouped in a separated strongly supported group (100% bootstrap support), closely related to botouliviruses, for which we support the proposal of Nerva and coworkers (21) of a new genus of botourmiaviruses named *Penoulivirus*. *B. cinerea* mitoviruses BcMV5, BcMV7, and BcMV8, and the variants BcMV2-BCS8, BcMV4-BCS16, and SsMV4-Bc are included in different groups closely related to the recognized members of the *Mitovirus* genus as Ophiostoma novo-ulmi mitoviruses 4, 5, and 6. BcMV6 and -9, and the variants BcMV1-BCS1, BcMV3-BCS16, and SsMV3-Bc are placed in different groups that are phylogenetically closer to plant mitoviruses and associated with the recognized members of the genus *Mitovirus*, Ophiostoma mitovirus 3a, and Cryphonectria parasitica mitovirus 1. Figure 5 shows that BcNV4 was grouped with the recognized Saccharomyces cerevisiae 20S and 23S RNA narnaviruses, suggesting that this may be considered a new member of the genus *Narnavirus*. However, *B. cinerea* binarnaviruses 1, 2, 3, and 5 were grouped in a strongly supported clade (100% bootstrap support), together with other mycoviruses, and separated from the group including other bisegmented narnavirus (Matryoshka RNA virus and Leptomonas seymouri narna-like virus) (52, 53) and from the group of the true narnaviruses. We propose the creation of a new genus named *Binarnavirus* (bisegmented naked RNA virus), inside a new family named *Binarnaviridae* to include the new group of bisegmented mycoviruses identified in the present study.

### (ii) Endornavirus.

The family *Endornaviridae* consists of two virus genera, *Alphaendornavirus* and *Betaendornavirus*, that include capsidless viruses with ssRNA+ genomes that range from 9.7 to 17.6 kb with a single ORF (54). Two endornaviruses were found to infect *B. cinerea* samples, Botrytis cinerea endornavirus 2 (BcEV2) and BcEV3, with genomes of 13,581 and 13,582 nt, respectively (Table 1; see also Fig. S4A), and identities of 84.50 and 92.90% at the nucleotide and amino acid levels between them. Both have a poly(C) at the 3′ end, indicating that they are complete at this end, and encode a protein of 4,501 aa with the RdRp domain located in the C-terminal region, with the typical motifs A to G highly conserved in comparison with the reference genome BcEV1 (see Fig. S4B) (55). This protein also contains a viral methyl transferase (MTR) domain, a DExH box (DEXDc domain), a viral helicase superfamily 1 (Hel) domain, and the typical cysteine-rich region between aa 1265 and 1336 (see Fig. S4A). The phylogenetic analysis indicated that BcEV2 and BcEV3 grouped together with Sclerotinia minor endornavirus 1 (56) in a strongly supported group (100% bootstrap value) inside the genus *Betaendornavirus* in the family *Betaendornaviridae*; BcEV1 was also included inside this genus but in a different group (see Fig. S4C). Both endornaviruses were present in the same three Italian pools. However, in Spain they were found in different pools with a low number of reads (Fig. 1C; see also Table S1).

10.1128/mBio.03705-20.4 FIG S4 Sequence properties of Botrytis cinerea endornavirus (BcEV) 2 and 3. (A) Schematic representation of BcEV RNA genomes showing the locations of ORFs and the alignment of the cysteine-rich region. (B) Amino acid sequence alignment of RdRps of endornaviruses (A to G) are shaded with light and dark gray colors. Asterisks indicate identical amino acid residues, and colons indicate similar residues that are not listed. Botrytis cinerea betaendornavirus 1 (BcEV-1; AOZ66245) was used as reference genome in the alignment. (C) Phylogenetic tree computed by MEGA-X stochastic algorithm to infer phylogenetic trees by maximum likelihood. Model of substitution, WAG+F + I+G5. Consensus tree is constructed from 1,000 bootstrap trees. Log likelihood of consensus tree, −155425.321. At nodes the percentage bootstrap values. Download FIG S4, TIF file, 1.6 MB. Copyright © 2021 Ruiz-Padilla et al. 2021 Ruiz-Padilla et al. https://creativecommons.org/licenses/by/4.0/ This content is distributed under the terms of the Creative Commons Attribution 4.0 International license.

### (iii) Hypovirus and Fusarivirus.

The family *Hypoviridae* includes one genus of capsidless viruses, *Hypovirus*, with ssRNA+ genomes ranging from 9.1 to 12.7 kb with one or two ORFs (57). Four new hypoviruses, Botrytis cinerea hypovirus 2 (BcHV2) to BcHV5, were found to infect *B. cinerea* samples, together with Sclerotinia sclerotiorum hypovirus 1 A (SsHV1A) (58), and BcHV1 and Botrytis cinerea hypovirus 1 satellite-like RNA (41) (Table 1). The alignment of the RdRp regions of BcHV1 to -5 and SsHV1 revealed some conservation in motifs I to VIII, with SDD or GDD in the core domain (see Fig. S5A). The new hypoviruses have genome lengths between 10,863 and 17,631 nt with low identities between them (16.89 to 25.87%), and BcHV2 and BcHV5 have a complete sequence at the 3′ end since both have the characteristic poly(A) tail (see Fig. S5B and E). BcHV2, -3, and -5 encode a single protein with a sizes of 4,199, 3,042, and 4,856 aa, respectively, and a conserved viral Hel domain, whereas BcHV3 contains the conserved domains of UDP glycosyltransferase and Hel (see Fig. S5B). A papain-like protease domain, involved in hypovirus polyprotein processing (57), is also present in BcHV3 and BcHV5 proteins, whereas BcHV2 protein contains a 2A-like protease domain (DIEQNPGP, aa 1076 to 1083). BcHV4 is the only hypovirus that encodes two proteins of 1,023 and 3,345 aa, with the viral Hel domain conserved in the large protein, which has 37% identity with Rhizoctonia solani hypovirus 1 (27), and no conserved domains in the short one that has 58% identity with SsHV2 (59) (Table 1). BcHV3 and -4 were widely distributed in Italian and Spanish samples and were well represented by a high number of reads, whereas BcHV2 was only found in Spain, and BcHV5 was more frequent in Italy (Fig. 1C; see also Table S1). BcHV1 and SsHV1A were also broadly distributed and well represented in almost all pools from both countries, and BcHV1 satellite-like RNA was associated with its auxiliary virus in all pools except in two of them, one in Italy and another in Spain (Fig. 1C; see also Table S1).

10.1128/mBio.03705-20.5 FIG S5 Sequence properties of hypoviruses and fusariviruses. (A) Amino acid sequence alignment of RdRp (I to VIII) of Botrytis cinerea hypovirus (BcHV) 2 to 5 are shaded with light and dark gray colors. Asterisks indicate identical amino acid residues and colons show similar residues that are not listed. BcHV1 and Sclerotinia sclerotiorum hypovirus 1 (SsHV-1; AEL99352) was used as a reference genome in the alignment. (B) Schematic representation of BcHV2 to BcHV5 RNA genomes showing location of ORFs. (C) Amino acid sequence alignment of RdRps (A to G) of Botrytis cinerea fusarivirus (BcFV) 3 to 7. Sclerotinia sclerotiorum fusarivirus 1 (SsFV-1; AKJ26309), Rhizoctonia solani fusarivirus 2 (RsFV-2, MK558256.1) and Botrytis cinerea fusarivirus 1 (BcFV-1; MG554633.1) were used as reference genomes in the alignment. (D) Schematic representation of BcFV3 to BcFV7 RNA genomes showing the locations of ORFs. (E) Percent identity matrix of BcHV1 to BcHV5 and BcFV3 to BcFV7 generated by Clustal Omega 2.1. Identities from higher to lower are labeled from dark green to dark red, respectively. (F) Phylogenetic tree of fusarivirus and hypovirus computed by MEGA-X stochastic algorithm to infer phylogenetic trees by maximum likelihood. Model of substitution, WAG+F + I+G5. A consensus tree was constructed from 1,000 bootstrap trees. Log likelihood of consensus tree, –109668.538. At the nodes are the percent bootstrap values. Download FIG S5, TIF file, 2.6 MB. Copyright © 2021 Ruiz-Padilla et al. 2021 Ruiz-Padilla et al. https://creativecommons.org/licenses/by/4.0/ This content is distributed under the terms of the Creative Commons Attribution 4.0 International license.

We identified in our samples five novel fusariviruses, Botrytis cinerea fusarivirus 3 (BcFV3) to BcFV7. The genome length is in a range of 6.3 to 8.3 kb, coding for a hypothetical protein (ORF2), with a size between 491 and 704 aa, and identities of 15 to 73% between them, and the replicase (ORF1), containing RdRp and Helicase domains, and a size ranging from 1,542 to 1,675 aa (see Fig. S5D). RdRp motifs are highly conserved, with the GDD in the core domain, since it has been shown in the alignment with Botrytis cinerea fusarivirus 1 (BcFV1) (41) and Sclerotinia sclerotiorum fusarivirus 1 (SsFV1) (60) (see Fig. S5C). The identity between these new fusariviruses at the amino acid level of the replicase varied from 27.73% between BcFV4 and BcFV6 to 85.15% between BcFV5 and BcFV6 (see Fig. S5E); the sequence of both was complete at the 3′ end, since they ended in a poly(A) tail like other fusariviruses. These viruses were present in some pools, and the number of reads varied depending on the fusarivirus and the sample (Fig. 1C; see also Table S1).

The results of an analysis of the phylogenetic relationships between hypoviruses and fusariviruses are shown in Fig. S5F. All mycoviruses were classified inside their genera, and fusariviruses grouped into three different groups was strongly supported (100% bootstrap support). BcFV3 and -4 were placed together in a group with the other fusariviruses found in *B. cinerea*, BcFV1. Hypoviruses were also distributed in three different groups, all supported by a 99 to 100% bootstrap value, with BcHV4 and -5 together in one group, BcHV1-BCS11 with BcHV3 in another group, and BcHV2 in a third group.

### (iv) Other ssRNA+ viruses.

**FIG 6:**
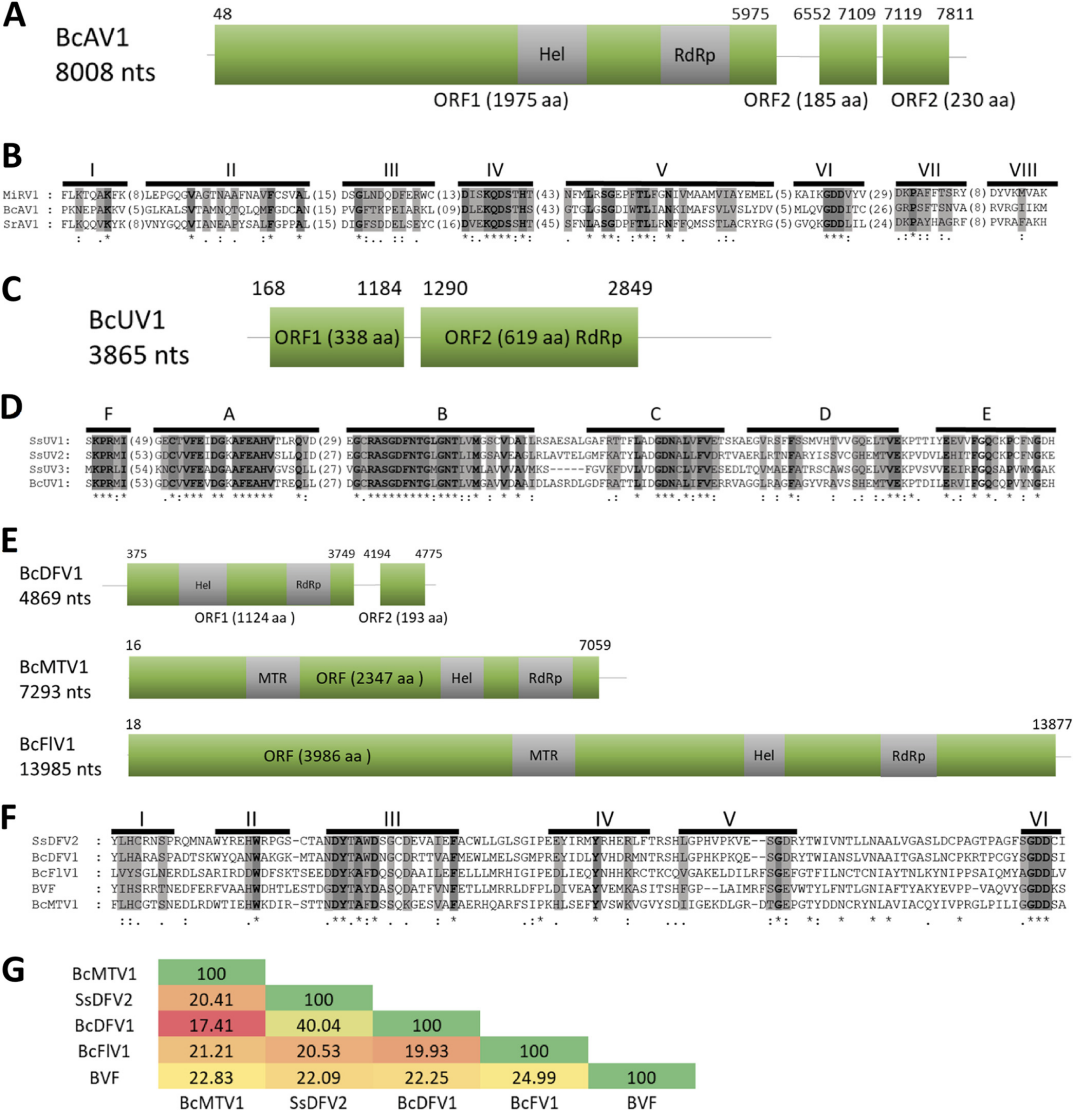
Sequence properties of *Botrytis cinerea* ssRNA+ mycoviruses. The conserved motifs of the RdRps are shaded with light and dark gray colors. Asterisks indicate identical amino acid residues, and colons indicate similar residues. Amino acids in parentheses show the positions of amino acid residues that are not listed. (A) Schematic representation of Botrytis cinerea alpha-like virus 1 (BcAV1) RNA genome showing location of ORFs. (B) Amino acid sequence alignment of alphavirus RdRps (I to VIII), with the reference genomes Morchella importuna RNA virus 1 (MiRV1, MK279480.1) and Sclerotium rolfsii alphavirus-like virus 1 (SrAV1, MH766488.1). (C) Schematic representation of Botrytis cinerea umbra-like virus 1 (BcUV1) RNA genomes showing the locations of ORFs. (D) Amino acid sequence alignment of umbravirus RdRps showing conserved motifs F to E, with the reference genome Sclerotinia sclerotiorum umbra-like virus 1 (SsUV1, KC601995). (E) Schematic representation of Botrytis cinerea deltaflexivirus 1 (BcDFV1), Botrytis cinerea mycotymovirus 1 (BcMTV1), and Botrytis cinerea flexivirus 1 (BcFlV1) RNA genomes showing the locations of ORFs. (F) Amino acid sequence alignment of mycoviruses tymo-like RdRp showing conserved motifs I to VIII, with the reference genomes of Sclerotinia sclerotiorum deltaflexivirus 2 (SsDFV2, MH299810) and Botrytis virus F (BVF, LN827953). (G) Percent identity matrix generated by Clustal Omega 2.1. Identities from higher to lower are labeled from dark green to dark red, respectively.

The genus *Alphavirus* is monopartite, with a genome of 9.7 to 12 kb ssRNA+ (61). One novel alphavirus-like sequence, Botrytis cinerea alpha-like virus 1 (BcAV1), was identified for the first time infecting *B. cinerea* in several Spanish and Italian pools with different concentrations based in the number of reads in each pool (Fig. 1C; see also Table S1). BcAV1 has a genome of 8.0 kb coding for a protein with the domain of a viral RNA helicase (superfamily 1) and an RdRp, as well as two other hypothetical proteins of 185 and 230 aa, with no identity to any other proteins in the databases (Fig. 6A). The eight (I to VIII) conserved motifs of the RdRp are shown in the alignment with the same region of Sclerotium rolfsii alphavirus-like virus 1 (SrAV1) (25) and Morchella importuna RNA virus 1 (MiRV1) (8) (Fig. 6B). This is the first reported alpha-like mycovirus associated with *B. cinerea*.

Umbra-like mycoviruses were also found in several pools from both countries, Sclerotinia sclerotiorum umbra-like virus 2 (SsUV2) and SsUV3 (58), which were annotated as variants of the *B. cinerea* isolates from grapevine, and Botrytis cinerea umbra-like virus 1 (BcUV1). SsUV2 and -3 were frequently found in several pools from both countries. The novel mycovirus BcUV1 is the first umbra-like virus reported to be associated with *B. cinerea* and was found with a high number of reads only in the three pools from southern Spain (Fig. 1C; see also Table S1), suggesting that it probably is restricted to this area. BcUV1 represents a new umbra-like mycovirus based on the low identities at the amino acid level compared to SsUV2 and SsUV3 (46.52 and 35.10%, respectively); however, the highest identity was found with SsUV1 (5) (50.77%), which was not present in any pool in this work (Table 1). BcUV1 has a ssRNA+ genome of 3,865 nt with two ORFs; the first one encodes a protein of 338 aa, and the second one encodes the putative RdRp protein of 619 aa (Fig. 6C). The RdRp contains the conserved motifs A to F, since it is shown in the alignment with SsUV1, -2, and -3; however, in the RdRp sequences the core domain has a GDN triplet, a motif that is often found in mononegaviruses and polymycoviruses but not in ssRNA+ viruses (Fig. 6D).

Several viral sequences related with viruses of the order *Tymovirales* were found in our samples (Table 1). Among these was the already-described Sclerotinia sclerotiorum deltaflexivirus 2 (SsDFV2) (26), which was annotated as a *B. cinerea* variant, and Botrytis virus F (BVF) (23). In addition, three novel mycoviruses named Botrytis cinerea deltaflexivirus 1 (BcDFV1), Botrytis cinerea flexivirus 1 (BcFlV1), and Botrytis cinerea mycotymovirus 1 (BcMTV1) (Table 1) were identified, with an identity at the amino acid level ranging from 17.41% between BcDFV1 and BcMTV1 to 40.04% between BcDFV1 and SsDFV2 (Fig. 6G). All of these code for putative RdRp proteins with the GDD triplet in the core domain (Fig. 6E). The length of BcDFV1 is 4,869 nt and contains two ORFs encoding a protein of 1,124 aa with the putative domains of a viral helicase and an RdRp, and a hypothetical small protein of 193 aa (Fig. 6E). The genome size of BcFlV1 is 13,985 nt and encodes a long protein of 3,986 aa with a viral MTR, a viral Hel superfamily 1, and the RdRp domains. Finally, BcMTV1 has a genome of 7,293 nt and one single ORF coding for 2,347 aa with the same domains as BcFV1 (Fig. 6E). Flexiviruses and deltaflexiviruses were present in one to three pools in both countries; however, BcMTV1 and BVF were distributed for several Spanish and Italian regions (Fig. 1C; see also Table S1).

**FIG 7:**
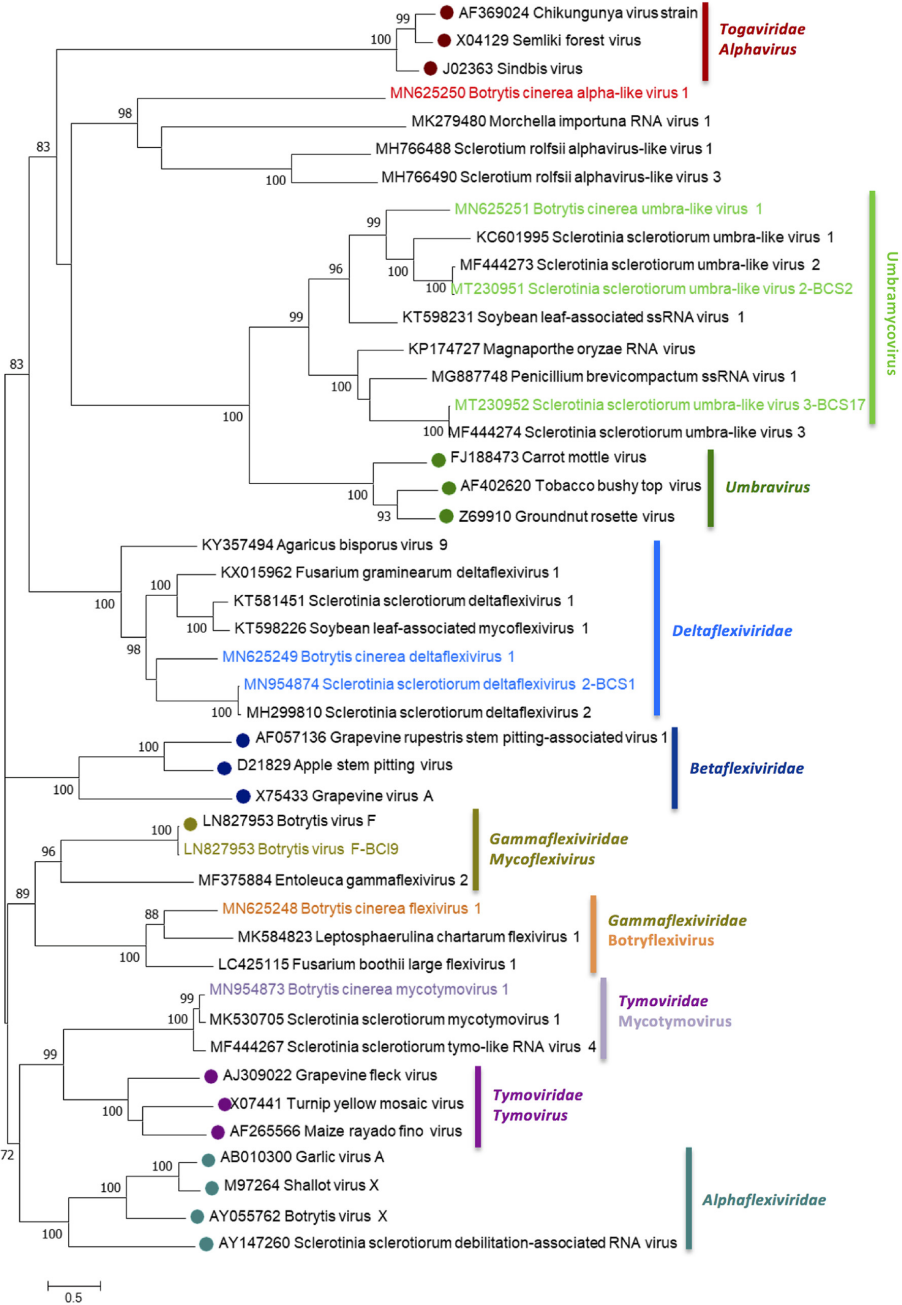
*Botrytis cinerea* ssRNA+ mycoviruses phylogenetic tree. Maximum- likelihood phylogenetic tree of the amino acid sequences of RdRps computed by MEGA-X stochastic algorithm (model of substitution: WAG+F+I+G5). A consensus tree was constructed from 1,000 bootstrap trees (log likelihood of consensus tree, –125555.453). All bootstrap values (%) of >65 are represented at each node of the tree. Branch lengths are proportional to the number of amino acid substitutions and are measured by a scale bar.

The phylogenetic relationships between all of the described ssRNA+ mycoviruses are shown in Fig. 7. BcAV1 is included in a strongly supported group (98% bootstrap support) with other alpha-like mycoviruses and separated from the group of accepted alphaviruses inside the family *Togaviridae*; this mycoviral clade should probably be considered a new genus. This group is related to another one composed of members of the genus *Umbravirus*, and a group that contains all umbra-like mycoviruses detected in our study, including the new described BcUV1. We consider that these umbra-like viruses should be included in a new genus named *Umbramycovirus*. BcDFV1 and SsDFV2-BCS1 are included in the group of the *Deltaflexiviridae* family; thus, both should be considered members of this family. BcFlV1 is placed in a group with other mycoviruses closely related to BVF, the representative member of the genus *Mycoflexivirus* inside the family *Gammaflexiviridae*. These mycoviruses should be considered members of the genus *Mycoflexivirus* or be included in a new genus of viruses, inside the family *Gammaflexiviridae*, named *Botryflexivirus*. BcMTV1 is in a clade with members of the genus *Tymovirus*, of the family *Tymoviridae*, but in a different group (100% bootstrap support) with other mycoviruses, clearly separated from the plant tymoviruses. Consequently, these mycoviruses could be considered members of a new genus named *Mycotymovirus* inside the family *Tymoviridae*.

### Negative single-stranded RNA mycoviruses.

Fifteen ssRNA– viral sequences identified in our samples can be ascribed to orders *Mononegavirales* and *Bunyavirales*, inside the phylum *Negarnaviricota*, based on their RdRp amino acid sequences (46). Twelve were new mycoviruses named Botrytis cinerea negative-stranded RNA virus 2 (BcNSRV2) to BcNSRV11, Botrytis cinerea orthobunya-like virus 1 (BcOBV1), and Botrytis cinerea bocivirus 1 (BcBV1), and three of them were variants of BcNSRV7 and BcOBV1 (Table 1).

### (i) *Mononegavirales*-related mycoviruses.

BcNSRV3, -4, -5, and -7 have genomes with lengths ranging from 9.2 to 10.3 kb and four nonoverlapping ORFs. The longest ORF2 encodes a protein of 1,934 aa to 1,953 aa, which contains the mononegaviral RdRp domain, and also the mononegaviral mRNA-capping region V (MNC) essential for mRNA cap formation, indicating that probably all of these viruses are capped at the 5′ end (see Fig. S6A). The alignment with the reference genomes of Botrytis cinerea mymonavirus 1 (BcMymV-1) (12) and Sclerotinia sclerotiorum negative-stranded RNA virus 1 (SsNSRV-1) (11) showed high conservation of RdRp motifs “a” and “A to D” (see Fig. S6B). BcNSRV5 and -7 ORF1, -3, and -4 code for hypothetical proteins (HPs) with identities of 28.04, 58.27, and 60.38% between HP1, HP2, and HP3, respectively, but with no significant sequence similarity with other proteins in the database. BcNSRV3 and -4 ORF1 codes for the protein gp6 and HP, respectively, with 27.65% identity between them and around 30% identity with SsNSRV1 gp6. ORF3, from BcNSRV3 and -4, encodes protein gp2, with 38.38% of identity between them, and >40% identity with Sclerotinia sclerotiorum negative-stranded RNA virus 3 gp2 and SsNSRV1 nucleoprotein, suggesting that this protein is probably involved in encapsidation. BcNSRV3 ORF4 codes for gp1 that showed 26% identity with the gp1 protein of SsNSRV3, whereas BcNSRV4 ORF4 codes for a hypothetical protein HP2 showing 22.27% identity with BcNSRV3 gp1. The identities at the amino acid level of the RdRps among the four new mycoviruses varied from 24.47% between BcNSRV4 and -5 to 76.80% between BcNSRV5 and -7 (see Fig. S6C). The identity between BcNSRV7 and its mycoviral variants was >85% at the nucleotide level in the full genomic sequence, and it was >98% at amino acid level of the longer protein and 88% for the remaining proteins. A variable repeated sequence of 17 nt, 3′-CCUAAGUUUU(A/C)UUAAAU-5′, was found in the intergenic regions of all of the described mycoviruses (data not shown). These highly conserved gene junction sequences are present in the noncoding intergenic regions of members of the *Mymonaviridae* family (62). The four mycoviruses were present in a few Spanish pools, with coinfection of BcNSRV3 and -5 or of BcNSRV4 and -7 in one or two pools; however, BcNSRV7 was widely distributed in Italian pools (Fig. 2A; see also Table S1).

10.1128/mBio.03705-20.6 FIG S6 Sequence properties of Botrytis cinerea negative-stranded RNA virus 3 (BcNSRV3), -4, -5, and -7 and bunya-like viruses. The percent identity matrix was generated by using Clustal Omega 2.1. Identities from higher to lower are labeled from dark green to dark red, respectively. (A) Schematic representation of ssRNA– genomes showing the locations of ORFs. (B) Amino acid sequence alignment of RdRp of mononegavirales (premotif A and motifs A to D) shaded with light and dark gray colors. Asterisks indicate identical amino acid residues, and colons indicate similar residues that are not listed. Botrytis cinerea mymonavirus 1 (BcMymV-1, MH648611) and Sclerotinia sclerotiorum negative-stranded RNA virus 1 (SsNSRV-1; AHW76811) were used as reference genomes in the alignment. (C) Percent identity matrix of BcNSRV3, -4, -5, and -7. (D) Schematic representation of ssRNA– genomes of Botrytis cinerea negative-stranded RNA virus 2 (BcNSRV2), -6, -8, -9, -10, and -11 and Botrytis cinerea orthobunya-like virus 1 (BcOBV1) showing the locations of ORFs. (E) Percent identity matrix of bunya-like mycoviruses. Download FIG S6, TIF file, 1.6 MB. Copyright © 2021 Ruiz-Padilla et al. 2021 Ruiz-Padilla et al. https://creativecommons.org/licenses/by/4.0/ This content is distributed under the terms of the Creative Commons Attribution 4.0 International license.

### (ii) *Bunyavirales*-related mycoviruses.

For BcNSRV2, -6, -8, -9, -10, and -11 and BcOBV1, only a single RNA segment was found, with a size varying between 6.5 and 10.3 kb and with an ORF coding for a putative bunya-like RdRp varying in size from 2,135 aa in BcNSRV2 to 3,408 aa in BcNSRV10 (see Fig. S6D). All of these viruses showed similarity to the already-described BcNSRV1 (4), with the exception of BcNSRV2, which was more similar to Coniothyrium diplodiella negative-stranded RNA virus 1 (21), and of BcOBV1, which showed identity to Cachoeira porteira orthobunyavirus (63) (see Fig. S6E). The identities among RdRp sequences of BcNSRV8, -9, -10, and -11 were >64%; however, the identities among the other mycoviruses were much lower (see Fig. S6E).

**FIG 8:**
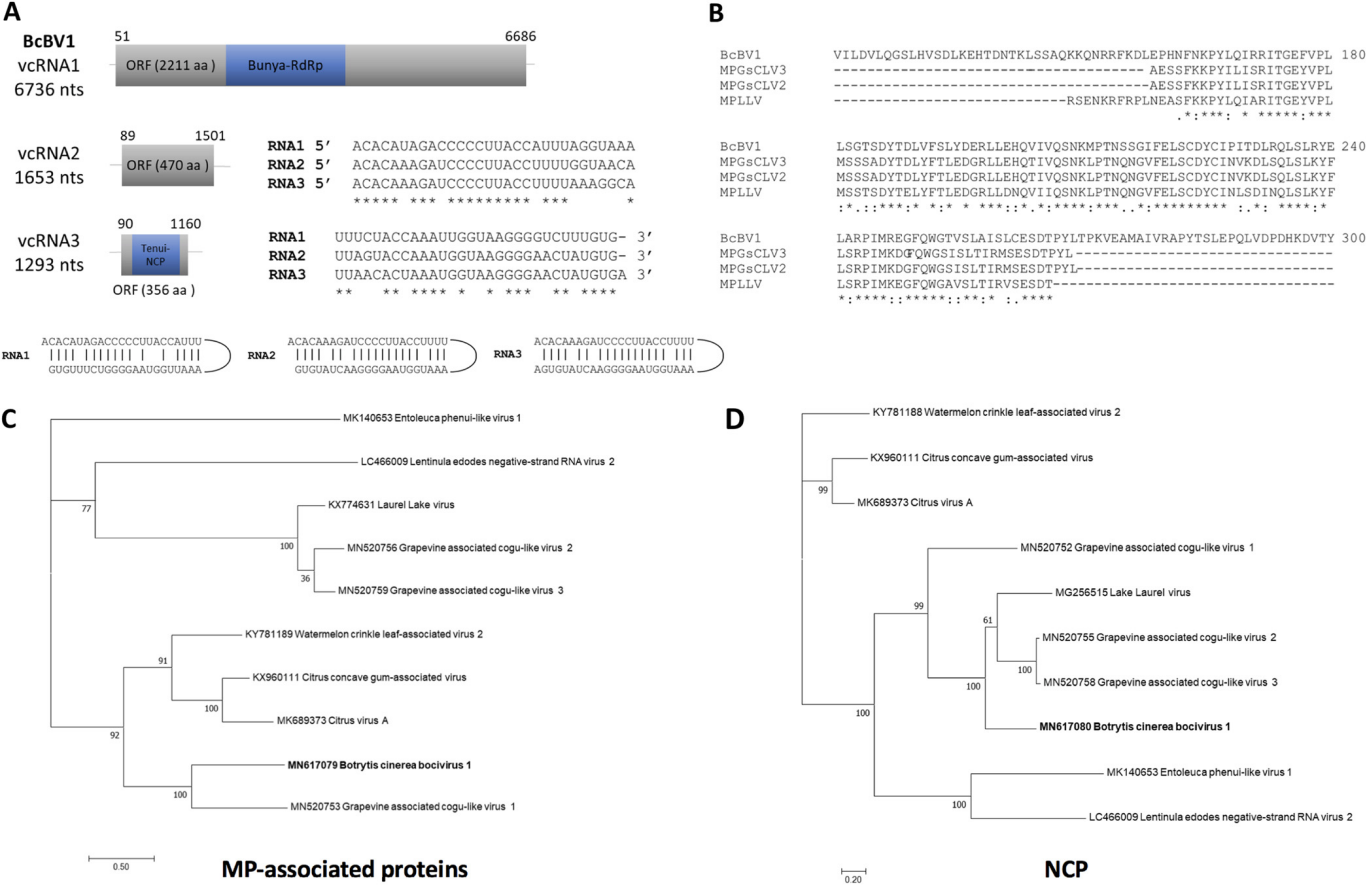
Sequence properties of Botrytis cinerea bocivirus 1. (A) Schematic representation of Botrytis cinerea bocivirus 1 (BcBV1) vcRNA (viral RNA complementary strand) segments showing the locations of ORFs, alignment of the conserved sequences of the 5′ and 3′ ends of BcBV1 RNA1, -2, and -3, and the panhandle structures formed by the 5′ and 3′ termini of each RNA segment. (B) Alignment of the conserved region of movement protein (MP)-like proteins of Grapevine-associated cogu-like virus 2 (GsCLV2; MN520754) and GsCLV3 (MN520757), Laurel Lake virus (LLV; KX774630), and BcBV1 RNA2. (C) Phylogenetic tree of MP-associated proteins computed by IQ-TREE stochastic algorithm to infer phylogenetic trees by maximum likelihood (model of substitution: VT+G4). A consensus tree was constructed from 1,000 bootstrap trees (log likelihood of consensus tree, −9944.852649). All bootstrap values (%) of >65 are represented at each node of the tree. Branch lengths are proportional to the number of amino acid substitutions and are measured by a scale bar. (D) Phylogenetic tree of nucleocapsid proteins (NCP) computed by using an IQ-TREE stochastic algorithm to infer phylogenetic trees by maximum likelihood (model of substitution: LG+G4). A consensus tree was constructed from 1,000 bootstrap trees (log likelihood of consensus tree, −6678.055394).

BcBV1 is the first putative trisegmented ssRNA– described that is associated with *B. cinerea* (Fig. 8A). Three segments, each with a single ORF, were found associated with the mycovirus. RNA1 is 6.7 kb, with an ORF coding for a protein of 2,211 aa with a 39.58% identity to Watermelon crinkle leaf-associated virus 1 (64) and the domain of a viral Bunya-RdRp superfamily, indicating that this protein could be involved in BcBV1 replication; RNA2 is 1.6 kb with an ORF that encodes a hypothetical protein of 470 aa with a 51.30% identity to Laurel Lake virus (LLV) ORF1 (65) and a 30% identity to Watermelon crinkle leaf-associated virus 1 movement protein, and RNA3 is 1.2 kb with an ORF coding for a protein of 356 aa with a 35.26% identity to Citrus virus A (66), with the domain of the tenuivirus nucleocapsid protein (NCP), which could be involved in BcBV1 encapsidation. These mycoviral segments showed identity to the recent discovered Grapevine-associated cogu-like viruses (67). The alignment of the three proteins showed that the RdRp had 44% identity with the RdRp of Grapevine-associated cogu-like virus 1 (GaCLV1), the putative NCP showed 42% identity to the putative nucleocapsid of GaCLV1, and the hypothetical protein had 57% identity to the putative movement proteins of Grapevine-associated cogu-like virus 2 (GaCLV2) and GaCLV3. A motif search found the core domain of 30K viral movement proteins (pfam17644, 30K_MP_core) in the putative movement proteins of GaCLV2 and -3 and in ORF1 LLV. The alignment of the core domains of these proteins with the hypothetical protein of BcBYV1 showed a high conservation in the corresponding region (Fig. 8B). Different failed attempts to amplify the RNA2 sequence with the RNA3 sequence, with primers in different combinations, mimicking a possible ambisense segment as with other confirmed coguviruses (66, 68), suggested that RNA2 and RNA3 are indeed separated segments. The trisegmented nature of the BcBV1 genome was also confirmed by determination of the 5′ and 3′ ends of the three genomic segments (Fig. 8A). BcBV1 RNAs shared almost identical nucleotide sequences in their 5′ and 3′ termini, as expected; like other similar segmented ssRNA– viruses, both termini are complementary to each other, forming a panhandle structure (66) (Fig. 8A).

The RdRp sequences of BcNSRV8, -9, -10, and -11 were aligned with the RdRp sequence of BcNSRV1 (4) to show the six conserved motifs: premotif A and and motifs A to E, which represent conserved regions of the RdRps of the family *Bunyaviridae* (data not shown). Motif C (SDD) was also conserved in BcNSRV2 and -6, BcOBV1, and BcBV1 RdRps, with the three basic residues (K, R, and R/K) inside the premotif A, which are also conserved in bunyavirus RdRps (69; data not shown). BcNSRV6 was well represented in one Italian pool, and BcNSRV2 and BcBV1 were present in a single distinct Spanish pool, with the BcBV1 RNA1 and -3 represented by almost three times as many reads as RNA2 (Fig. 2A; see also Table S1). BcNSRV8, -9, -10, and -11 were found in several Spanish pools with high number of reads; however, BcOBV1 and its mycoviral variant were the only ones widely distributed between Italy and Spain and not always in the same pools (Fig. 2A; see also Table S1).

### (iii) Phylogenetic relationships of negative-stranded mycoviruses.

The phylogenetic relationships of the proteins encoded by BcBV1 RNA2 and -3 are shown in Fig. 8C and D, respectively. The hypothetical protein of BcBV1 RNA2 is placed in a strongly supported group with putative movement proteins of GaCLV1, -2, and -3 and ORF1 of LLV (Fig. 8C) in the same clade with Entoleuca phenui-like virus protein. The putative BcBV1 NCP is grouped in a clade with the NCP of GaCLV1 and the classified coguviruses (Fig. 8D), reinforcing the hypothesis that it could be involved in mycoviral encapsidation.

**FIG 9:**
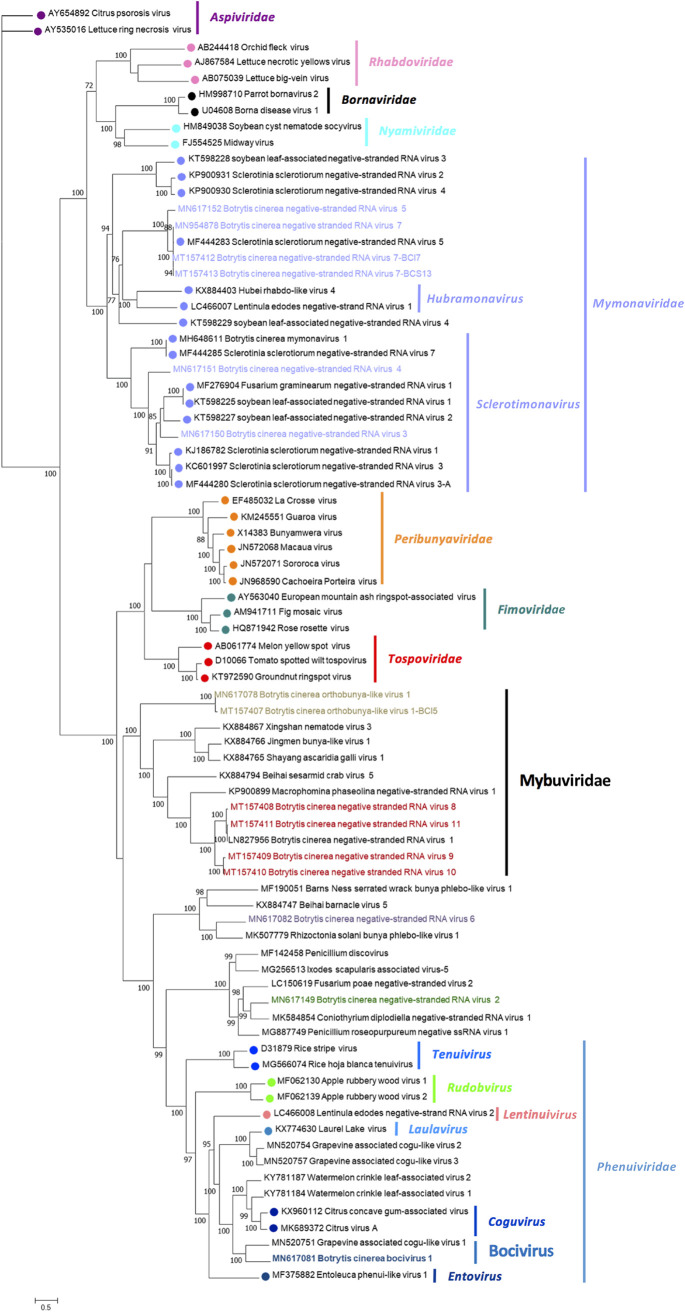
ssRNA– virus phylogenetic tree. A phylogenetic tree was computed by using the IQ-TREE stochastic algorithm to infer phylogenetic trees by maximum likelihood (model of substitution: VT+F+I+G4). A consensus tree was constructed from 1,000 bootstrap trees (log likelihood of consensus tree, –316277.482879). All bootstrap values (%) of >65 are represented at each node of the tree. Branch lengths are proportional to the number of amino acid substitutions and are measured by a scale bar.

The phylogenetic relationships between the discovered mycoviruses and other ssRNA– viruses are shown in Fig. 9. BcNSRV3, -4, -5, and -7 were included in the order *Mononegavirales*. BcNSRV5 grouped with BcNSRV7, its two mycoviral variants, and Sclerotinia sclerotiorum negative-stranded RNA virus 5 (58) in a strongly supported clade (100% bootstrap value), one closely related to the new created genus *Hubramonavirus* inside the family *Mymonaviridae* (62, 70), where they could be included. These *B. cinerea* mycoviruses were clearly separated from the well-supported group containing BcNSRV3 and -4, BcMyV1, and other members of the genus *Sclerotimonavirus*, inside the family *Mymonaviridae*, represented by SsNSRV1. BcNSRV3 and -4 might be considered new members of the genus *Sclerotimonavirus*. The remaining ssRNA– mycoviruses grouped with members of the order *Bunyavirales*. BcNSRV8, -9, -10, and -11 are included in a group with BcNSRV1 inside a clade (100% bootstrap value) that includes viruses infecting fungi and other hosts; we propose the creation of a new family named *Mybuviridae*, probably including several genera. BcOBV1 and its variant are in a separate but well-supported group, which could be considered also inside this new proposed family. BcNSRV2 and -6 and BcBV1 were included in a clade with other members of the family *Phenuiviridae*, but in different groups. BcNSRV2 and -6 are in two different groups, highly supported, inside the family *Phenuiviridae*, but probably establishing two new different genera. BcBV1 grouped with GaCLV1 and members of the genus *Coguvirus*, but most probably constituting a new genus inside the family *Phenuiviridae*, for which we propose the name *Bocivirus*.

### Double-stranded RNA mycoviruses.

A total of 19 mycoviruses with dsRNA genomes were identified and classified as botybirnaviruses (*n* = 2), quadriviruses (*n* = 1), victoriviruses (*n* = 11), partitiviruses (*n* = 2), and unclassified dsRNA viruses (*n* = 3) (Table 1).

Botybirnaviruses have a linear segmented genome composed of two RNAs (71). *B. cinerea* samples were infected with two botybirnaviruses, the already-characterized Botrytis porri botybirnavirus 1 (BpRV1) (72), which was annotated as a *B. cinerea* variant, and the newly identified Botrytis cinerea botybirnavirus 2 (BcBV2). Both mycoviruses were present in one Spanish pool but not in the same one (Fig. 2B; see also Table S1). The BcBV2 genome comprises two dsRNA segments 6 kb in length, dsRNA1 encodes a protein of 1,831 aa with the conserved motifs (I to VIII) of the RdRps, including the highly conserved triplet GDD in motif VI, and with identity to the cap-pol fusion protein of Botryosphaeria dothidea botybirnavirus 1 (73); dsRNA2 encodes a hypothetical protein of 1,801 aa, with identity to the hypothetical protein of Botrytis cinerea botybirnavirus 1 (71), and with an F-box domain (E value = 5.50E–04), according to CDD/SPARCLE results (74), present in the N-terminal half of the protein (see Fig. S7A and B). An alignment of the nucleotide sequences of both dsRNAs shows high similarity in the first 470 nt of the 5′ noncoding region and in the last 100 nt of the 3′ noncoding region (data not shown), as has been shown for other botybirnaviruses (72).

10.1128/mBio.03705-20.7 FIG S7 Sequence properties of dsRNA viruses. (A) Amino acid sequence alignment of RdRps showing motifs I to VIII of Botrytis cinerea botybirnavirus 2 (BcBV2) and Botrytis porri botybirnavirus 1 (BpRV1; MN954879). Botrytis cinerea botybirnavirus 1 (BcBV-1; MH321499) and Botryosphaeria dothidea botybirnavirus 1 (BdBV-1; MH684534) were used as reference genomes. Asterisks indicate identical amino acid residues and colons show similar residues that are not listed. (B) Schematic representation of BcBV2 genome showing location of ORFs. (C) Amino acid sequence alignment of RdRp of Botrytis cinerea victorivirus (BcVV) 2 and 3. Helminthosporium victoriae virus 190S (HV190s; AAB94791) and Botrytis cinerea victorivirus 1 (BcVV-1; MH347278) were used as reference genomes for the motif alignments. (D) Percent identity matrix generated by Clustal Omega 2.1 of BcVV2 and BcVV3 and variants. The level of identity from higher to lower is labeled from dark green to dark red, respectively. (E) Schematic representation of BcVV2 and BcVV3 genomes showing location of ORFs. (F) Amino acid sequence alignment of RdRp of Botrytis cinerea mycovirus 3 (BcMyV3), Botrytis cinerea mycovirus 5 (BcMyV5), and Botrytis cinerea partitivirus 3 (BcPV3). Penicillium stoloniferum virus S (PSVS; AAN86834), Fusarium graminearum dsRNA mycovirus 5 (FgMV5; KX380787), and Cryphonectria parasitica bipartite mycovirus 1 (CpbMV1, KC549809) were used as reference genomes for the motif alignments. (G) Schematic representation of BcMyV3, BcMyV5, and BcPV3 dsRNA genomes showing the locations of ORFs. Download FIG S7, TIF file, 2.6 MB. Copyright © 2021 Ruiz-Padilla et al. 2021 Ruiz-Padilla et al. https://creativecommons.org/licenses/by/4.0/ This content is distributed under the terms of the Creative Commons Attribution 4.0 International license.

*Totiviridae* family includes the genus *Victorivirus* (75). Victoriviruses have a genome of ∼6 kb with two large ORFs. An incomplete sequence of Botrytis cinerea victorivirus 1 was detected in our *B. cinerea* samples. In addition, two new victoriviruses were identified and named Botrytis cinerea victorivirus 2 (BcVV2) and BcVV3 with genomic lengths of 5.2 kb with two overlapping ORFs: the first one encodes the putative coat protein of 807 aa, and the second one encodes the putative RdRp of 838 aa, with all motifs (I to VIII) highly conserved in comparison to Helminthosporium victoriae virus 190S (75), as a representative member of the genus *Victorivirus*, and other victoriviruses infecting *B. cinerea* and *Botryotinia fuckeliana* (see Fig. S7C and E). The stop codon of the CP ORF overlaps the start codon of the RdRp ORF in the tetranucleotide sequence “AUGA,” as also shown for other victoriviruses (75). Eight mycoviral variants of BcVV2 and BcVV3 were identified in our study, with an identity in the RdRp sequences of >94% compared to its reference viruses (see Fig. S7D). All victoriviruses were present in pool BCI8, and many of them were detected in at least one Spanish pool with a different representation based on the number of reads (Fig. 2B; see also Table S1).

**FIG 10:**
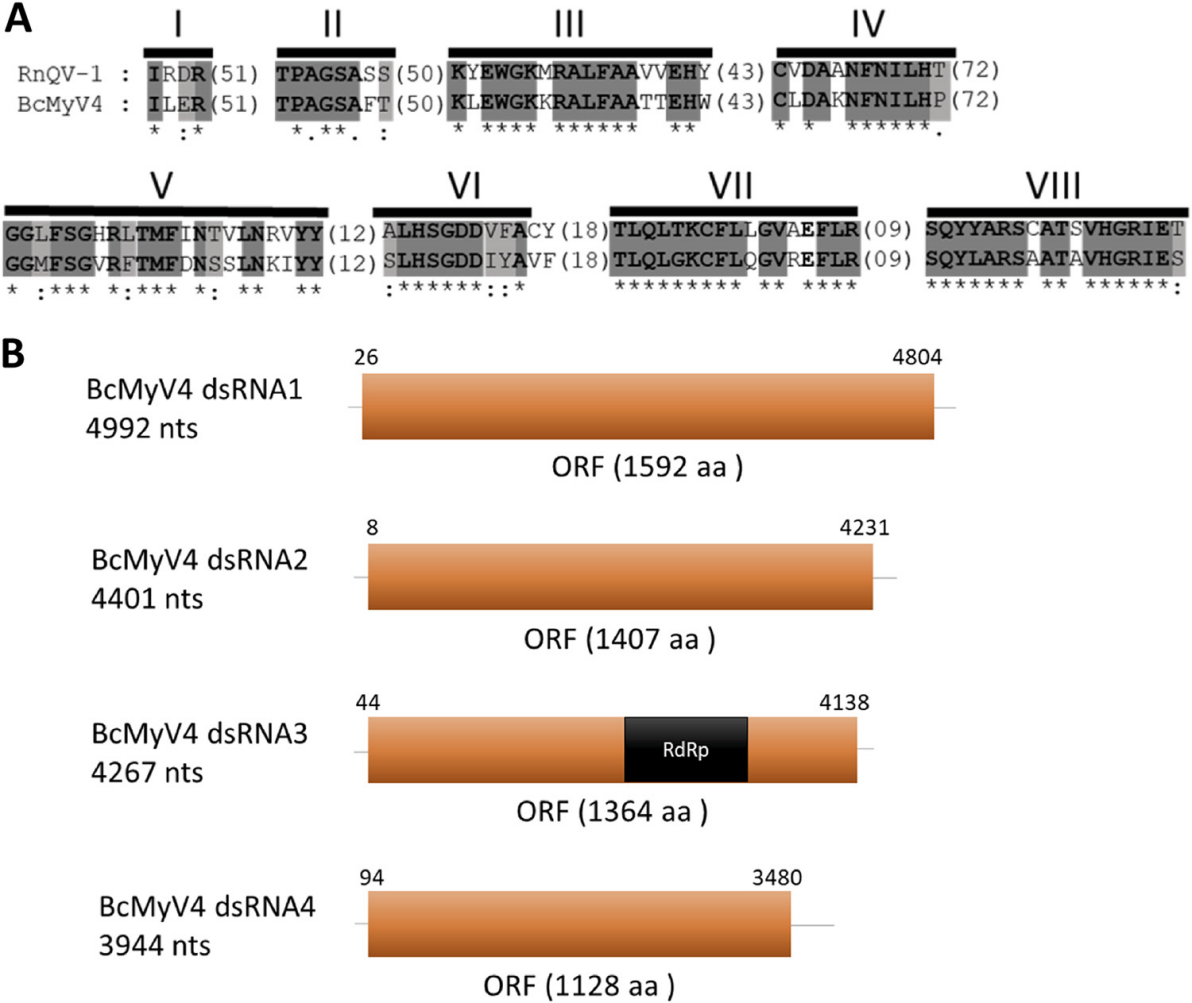
Sequence properties of Botrytis cinerea mycovirus 4. (A) Amino acid sequence alignment of RdRp of Botrytis cinerea mycovirus 4 (BcMyV4) and Rosellinia necatrix quadrivirus 1 (RnQV1; AB620061). (B) Schematic representation of the four RNA segments of BcMyV4 showing the locations of ORFs.

*Quadriviridae* family includes the genus *Quadrivirus* (76). Members of this genus have four dsRNA genomic segments ranging from 3.5 to 5 kb. Botrytis cinerea mycovirus 4 (BcMyV4) has a tetrasegmented dsRNA genome (Fig. 10A and B) with dsRNA1 and dsRNA4 encoded proteins (1,592 and 1,128 aa) showing 91 and 97% identities to two hypothetical proteins of the trisegmented Botrytis cinerea RNA virus 2; the dsRNA2 encoded protein (1,407 aa) had 24% identity to a structural protein of Rosellinia necatrix quadrivirus 1 (RnQV1) (77), and the dsRNA3 encoded protein (1,364 aa) had 95% identity to the RdRp of Botrytis cinerea RNA virus 2. Alignment of the putative BcMyV4 RdRp with the sequence of RnQVV1 RdRp showed a high conservation of all motifs (Fig. 10A). This is the first putative quadrivirus associated with *B. cinerea*. BcMy4 was only detected in Spanish pools, and mainly in the La Rioja region with dsRNA3 coding for the putative RdRp in higher abundance (Fig. 2B; see also Table S1 and Fig. S2).

The family *Partitiviridae* includes dsRNA viruses with two genome segments encapsidated independently (78). A partitivirus named Botrytis cinerea partitivirus 3 (BcPV3) was identified in only one Spanish pool (Fig. 2B; see also Table S1). BcPV3 dsRNA1 (1.8 kb) contains a single ORF coding for a putative RdRp (539 aa), with conservation of the motifs A to G of other partitiviruses; and BcPV3 dsRNA2 (1.5 kb) contains a single ORF coding for a putative coat protein (CP) (433 aa) (see Fig. S7G). BcPV3 RdRp showed an amino acid identity of 99.64% with the partial RdRp of Sclerotinia sclerotiorum partitivirus 3, and BcPV3 CP showed an amino acid identity of 92.84% with the CP of Sclerotinia sclerotiorum partitivirus 2 (58). BcPV3 dsRNA1 and dsRNA2 showed identities of around 45% at the nucleotide level and 12% at the amino acid level with other partitiviruses associated with *B. cinerea* (42, 44). The first 30 nt of both dsRNA segments were almost identical; however, there was no such conservation at the 3′ end, suggesting that the sequence of both or one of the dsRNA segments may be incomplete (data not shown). Botryotinia fuckeliana partitivirus 1 was highly abundant in one Spanish pool and was annotated as a mycoviral variant of *B. cinerea* (Table 1 and Fig. 2B; see also Table S1).

The bipartite virus Botrytis cinerea mycovirus 3 (BcMyV3) has a genome composed of two dsRNAs segments (see Fig. S7G). dsRNA1 (2,024 nt) has a single ORF coding for a protein of 607 aa with conserved motifs A to F of the RdRps; dsRNA 2 (1,780 nt) contains an ORF that encodes a hypothetical protein of 307 aa. Both segments showed similarity with Cryphonectria parasitica bipartite mycovirus 1 (KC549809) and were present only in four Spanish pools (Fig. 2B). Botrytis cinerea mycovirus 5 (BcMyV5) has also a bipartite genome with the longest RNA (2,184 nt) with an ORF coding for a putative RdRp of 675 aa, and the shortest RNA (1,522 nt) with an ORF coding for a hypothetical protein of 316 aa (see Fig. S7G). Both segments showed similarity with Fusarium graminearum dsRNA mycovirus 5 (79) and were poorly represented in one and four pools from Spain and Italy, respectively (Fig. 2B and Table 1; see also Table S1). A variant of dsRNA2 of 1.4 kb was also found (Table 1). BcMyV3 and -5 and BcPV3 shared conserved motifs (A to G), including triplets GDD in motif C and YPE in motif E (see Fig. S7F). The identity between BcMyV3 and BcMyV5 was close to 40%; however, the identity with BcPV3 was around 20%. Both mycoviruses showed conservation at the 5′ end of both dsRNA segments, as in the case of BcPV3, supporting the hypothesis that they are bisegmented mycoviruses (data not shown).

Sclerotinia sclerotiorum dsRNA mycovirus L was previously found associated with *B. cinerea* samples (23), and here it was also found in three pools of two different regions of Spain with a representative number of reads (Fig. 2B; see also Table S1).

**FIG 11:**
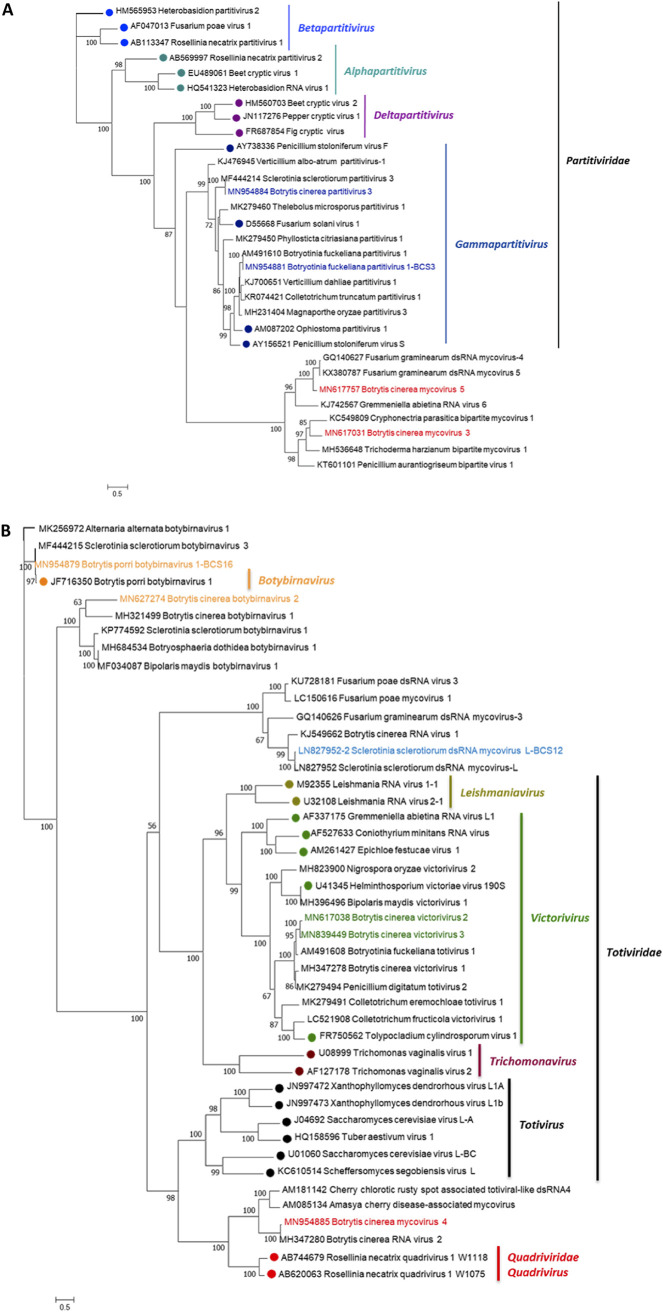
dsRNA virus phylogenetic tree. (A) Partitiviruses and other bisegmented dsRNA viruses. A phylogenetic tree was computed by using the IQ-TREE stochastic algorithm to infer phylogenetic trees by maximum likelihood (model of substitution: VT+F+I+G4). A consensus tree was constructed from 1,000 bootstrap trees (log likelihood of consensus tree, −29124.745182). All bootstrap values (%) of >65 are represented at each node of the tree. Branch lengths are proportional to the number of amino acid substitutions and are measured by a scale bar. (B) Botybirnavirus, totivirus, and quadrivirus phylogenetic tree. The phylogenetic tree was computed by using the IQ-TREE stochastic algorithm to infer phylogenetic trees by maximum likelihood (model of substitution: LG+F+I+G4). A consensus tree was constructed from 1,000 bootstrap trees (log likelihood of consensus tree, −83393.114516).

The results of an analysis of the phylogenetic relationships between all dsRNA mycoviruses associated with the *Partitiviridae* family are shown in Fig. 11A. BcPV3 is within the *Gammapartitivirus* group, inside the family *Partitiviridae*, and should be considered a new member of this genus. The other two bisegmented dsRNA mycoviruses, BcMyV3 and BcMyV5, are included in two different strongly supported groups of the same clade, which is closely related to the family *Partitiviridae*. This clade should be classified a new family that probably comprises two new genera, one including BcMyV3 and a second including BcMyV5. The relationships of the other dsRNA viruses are shown in Fig. 11B. BcMyV4 grouped in the same clade of the family *Quadraviridae*, genus *Quadrivirus*, but in a different well-supported group, indicating that it can probably be included in a different genus inside this family. BcVV2 and -3 are grouped with all members of the *Victorivirus* genus, suggesting that they should be recognized as new victoriviruses. BcBV2 is also in a separate group related to other botybirna-like viruses, but it is not in the same group as Botrytis porri botybirnavirus 1.

### Single-stranded DNA mycoviruses.

**FIG 12:**
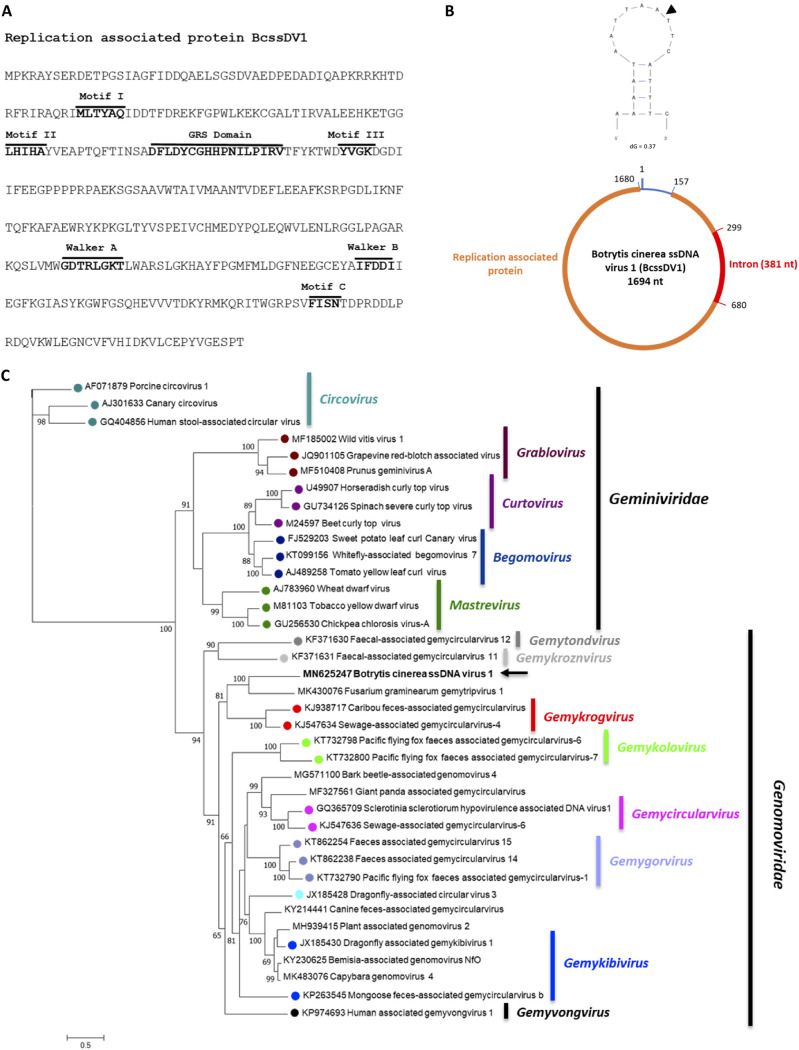
Sequence properties of Botrytis cinerea ssDNA virus 1 (BcssDV1). (A) Conserved motifs of genomovirus replication-associated proteins (motifs I to III, GRS domain, Walker A and B, and motif C) in BcssDV1. (B) Diagram showing the genome structure of BcssDV1 and stem-loop structure around the conserved nonanucleotide motif. (C) Phylogenetic tree computed by using the IQ-TREE stochastic algorithm to infer phylogenetic trees by maximum likelihood (model of substitution: VT+I+G4). A consensus tree was constructed from 1,000 bootstrap trees (log likelihood of consensus tree, −21005.690132). All bootstrap values (%) of >65 are represented at each node of the tree. Branch lengths are proportional to the number of amino acid substitutions and are measured by a scale bar.

Nowadays, no ssDNA virus has been described infecting *B. cinerea*. Here, we found several mycoviral sequences corresponding to a putative circular ssDNA virus, that we named Botrytis cinerea ssDNA virus 1 (BcssDV1). This mycovirus was found in one Italian pool from Lombardia and in eight Spanish pools from all regions except from the south of Spain and was very abundant, based on the read numbers, in seven of the nine pools (see Table S1). The mycoviral genome of 1,426 nt has a single ORF encoding a protein of 380 aa with identity to the spliced replication-associated protein of Bemisia-associated genomovirus NfO (KY230625). The putative replication-associated protein of 380 aa contains the domains of Gemini_AL1 (Geminivirus Rep catalytic domain; The AL1 proteins encodes the replication initiator protein [Rep] of geminiviruses) and the Gemini_AL1_M (Geminivirus rep protein central domain) and conserves all genomovirus characteristic amino acid motifs (Fig. 12A). Another sequence of 1,694 nt was found in our samples (Table 1); this mycoviral sequence has a single ORF coding for a protein of 321 aa that overlaps the last 321 aa of the protein of 380 aa (data not shown). An alignment of the nucleotide sequences showed a complete overlap except for a gap of 381 nt in the shorter sequence (see Fig. S8; BcssDV1l [ssDV1l] and BcssDV1s [ssDV1s]). We observed that BcssDV1s has a sequence duplication at its 5′ and 3′ ends (see Fig. S8, labeled in green); however, there was no such duplication of sequences between both ends in BcssDV1l, and we underlined the repeated sequence of BcssDV1s in the single sequence of BcssDV1l (see Fig. S8, underlined sequences). These observations suggest that the sequence of 1,694 nt (MN625247) is the probable correct sequence of the ssDNA mycovirus. The nucleotide sequence of the ssDNA mycovirus (1,694 nt) was further confirmed by Sanger sequencing of a product amplified with specific primers of the rep protein region used as the template viral DNA (see Table S1). In addition, the canonical donor GU and acceptor AG splicing sites were identified in the gap borders of the sequence, suggesting that this 381-nt sequence is probably an intron (see Fig. S8, labeled in pink in the 5′–3′ nucleotide sequence). In fact, deletion of the putative intron of BcssDV1l generates a new ORF coding for the protein of 380 aa. The putative nonanucleotide motif in the putative replication origin of BcssDV1 (1,694 nt) is highlighted in blue in Fig. S8, indicating with a blue arrow the position where the endonuclease activity of viral Rep introduces a nick in the virion-sense strand (AA_TT) (80). We have found several other sequences in different pools, related to BcssDV1, suggesting that its genome could be composed by more than one segment. Further analyses are in progress to determine the full sequence of all the putative segments of BcssDV1 genome. The possible structure of the viral Rep encoding ssDNA genome is shown in Fig. 12B, with a stem-loop structure around the conserved nonanucleotide. The phylogenetic relationships of BcssDV1 with other ssDNA viruses are shown in Fig. 12C. Members of the families *Geminiviridae* and *Genomoviridae* are clearly separated in the phylogenetic tree, with BcssDV1 inside the clade of the *Genomoviridae* family, closely related to gemykrogviruses, but in a different strongly supported group (100% bootstrap value) with the recently discovered Fusarium graminearum gemytripvirus (14), suggesting that both viruses should probably be considered members of a new genus inside the family *Genomoviridae*. The mycovirus Sclerotinia sclerotiorum hypovirulence-associated DNA virus 1 (13) and Bemisia-associated genomovirus NfO were in the same clade as BcssDV1 but in two different groups.

10.1128/mBio.03705-20.8 FIG S8 Nucleotide sequences of Botrytis cinerea ssDNA virus 1 short (BcssDV1s len = 1,426) and long (BcssDV1l len = 1,694). Labeled in green are the repeated sequences inside the BcssDV1s contig. Underlined is the conserved region in this repeated sequence between BcssDV1s and BcssDV1l. Labeled in blue in BcssDV1l is the putative nonanucleotide in the replication origin, with an arrow indicating the nick point. In boldface letters are shown the start and stop codons of the replication-associated protein. In red letters are shown the putative intron in BcssDV1l. In the alignment of both sequences, BcssDV1s and BcssDV1l, gray indicates the ORF, and pink indicates the donor and the acceptor site for splicing. Download FIG S8, DOCX file, 0.02 MB. Copyright © 2021 Ruiz-Padilla et al. 2021 Ruiz-Padilla et al. https://creativecommons.org/licenses/by/4.0/ This content is distributed under the terms of the Creative Commons Attribution 4.0 International license.

### Detection of mycoviruses infecting *B. cinerea in vivo*.

*B. cinerea* mycoviruses were detected by RT-qPCR (RNA viruses) or by qPCR (DNA virus) using specific primers (see Table S2) designed based on the sequence identified by high-throughput sequencing. The results are shown in Table 2. Forty-seven mycoviral sequences (belonging to 36 mycoviruses) were detected by high-throughput sequencing and qPCR in the same pools, using DNA or cDNA as the template, with the exception of two mycoviral sequences that were not detected in one of the pools. These 36 mycoviruses were selected to have a representation of mycoviruses with different genome classes (dsRNA, ssRNA, or ssDNA), including the most relevant ones found in our analyses. We included in the detection assay mycoviruses with multisegmented genomes to ensure that all putative associated genomic segments were inside the same pools, therefore supporting the hypothesis that they belong to the same mycoviral genome. The three segments of BcBV1 genome were detected in pool BCS15 by RT-qPCR and by conventional RT-PCR with specific primers and in 9 of the 10 individual samples included in the pool (Table 3). The two segments of BcBNV2 were also detected by RT-qPCR in all samples included in pool BCS14 (Table 3).

**TABLE 2:** Individual mycoviruses validated *in vivo* in *B. cinerea* samples

Mycovirus^*a*^	No. of pools
Botrytis cinerea mycovirus 3 RNA1	4/4
Botrytis cinerea mycovirus 3 RNA2	4/4
Botrytis cinerea botybirnarvirus 2 RNA1	1/1
Botrytis cinerea botybirnarvirus 2 RNA2	1/1
Botrytis porri botybirnavirus 1 RNA1	1/1
Botrytis porri botybirnavirus 1 RNA2	1/1
Botrytis cinerea mycovirus 5 RNA1	5/5
Botrytis cinerea mycovirus 5 RNA2	5/5
Botryotinia fuckeliana partitivirus 1 RNA1	4/4
Botryotinia fuckeliana partitivirus 1 RNA2	4/4
Botrytis cinerea mycovirus 4 RNA1	6/6
Botrytis cinerea mycovirus 4 RNA 2	6/6
Botrytis cinerea mycovirus 4 RNA 3	6/6
Botrytis cinerea mycovirus 4 RNA 4	6/6
Botrytis cinerea victorivirus 2_BCS9	2/2
Botrytis cinerea victorivirus 2_BCS14	4/4
Botrytis cinerea victorivirus 3	2/2
Botrytis cinerea alpha-like virus 1	20/20
Botrytis cinerea flexivirus 1	3/3
Botrytis cinerea deltaflexivirus 1	1/1
Botrytis cinerea umbra-like virus 1	3/3
Botrytis cinerea endornavirus 2	4/4
Botrytis cinerea endornavirus 3	4/4
Botrytis cinerea fusarivirus 3	1/1
Botrytis cinerea fusarivirus 5	5/5
Botrytis cinerea fusarivirus 7	8/8
Botrytis cinerea hypovirus 2	2/2
Botrytis cinerea hypovirus 3	11/11
Botrytis cinerea hypovirus 4	14/21
Botrytis cinerea binarnavirus 1	2/2
Botrytis cinerea binarnavirus 2 RdRp	1/1
Botrytis cinerea binarnavirus 2 HP	1/1
Botrytis cinerea narnavirus 4	3/4
Botrytis cinerea binarnavirus 5	11/11
Botrytis cinerea ourmia-like virus 9	7/7
Botrytis cinerea ourmia-like virus 11	11/12
Botrytis cinerea ourmia-like virus 16	23/23
Sclerotinia sclerotiorum mitovirus 4	20/20
Botrytis cinerea mitovirus 6	8/8
Botrytis cinerea mitovirus 5	22/22
Botrytis cinerea mitovirus 9	29/29
Botrytis cinerea bocivirus-RNA1	1/1
Botrytis cinerea bocivirus-RNA2	1/1
Botrytis cinerea bocivirus-RNA3	1/1
Botrytis cinerea orthobunya-like virus 1	13/13
Botrytis cinerea negative-stranded RNA virus 3	4/4
Botrytis cinerea negative-stranded RNA virus 6	1/1
Botrytis cinerea ssDNA virus 1	9/9

a Data are presented as the number of samples that tested positive/the total number of samples tested.

**TABLE 3:** Mycovirus pools and samples validated *in vivo* in *B. cinerea* samples

Pool	Validated mycovirus/samples	No. of samples validated	Total no. of samples/pool
BcssDV1			
BCI1	BCI1, BCI2, BCI3, BCI4, BCI5, BCI7	6	10
BCS15	BC108, BC110, BC119	3	10
BcBV1			
BCS15	BC107, BC109, BC110, BC112, BC113, BC114, BC116, BC117, BC119	9	10
BcBNV2 segments 1 and 2			
BCS14	BC89, BC90, BC91, BC93, BC95, BC96, BC97, BC98, BC101, BC103, BC104, BC106	12	12

10.1128/mBio.03705-20.10 TABLE S2 Primers used to detect mycoviral sequences. Download Table S2, DOCX file, 0.02 MB. Copyright © 2021 Ruiz-Padilla et al. 2021 Ruiz-Padilla et al. https://creativecommons.org/licenses/by/4.0/ This content is distributed under the terms of the Creative Commons Attribution 4.0 International license.

The multisegmented nature of BcBNV2 and BcBV1 was confirmed by detection of all genomic segments of both mycoviruses in single spore isolates BC93M1 and BC118M2, respectively, after vertical transmission of BcBNV2 and BcBV1 via spores (data not shown). The presence of BcssDV1 was also validated by conventional PCR or by qPCR in all pools found by high-throughput sequencing and in several independent samples of BCI and BCS15 pool (Tables 2 and 3). Also, the presence of BcssDV1 was detected by amplifying the fungal DNA, in a rolling-circle amplification assay, and by digestion with a specific enzyme present inside the mycovirus. Specific detection of the mycovirus was confirmed by cloning and sequencing of the PCR product.

## DISCUSSION

In the present study, viral metagenomics was applied on mixed fungal isolates using pools from different regions of two countries to search for novel mycoviruses. The advantage of this method is that it allows the identification of a great variety of new mycoviruses with different classes of genomes, including dsRNA, ssRNA+, ssRNA–, and ssDNA genomes. Moreover, the use of pools with several isolates let us to increase the number of samples analyzed increasing the probabilities to find a larger variety of mycoviruses. In parallel with this study, the virome associated with downy mildew (67, 81) and powdery mildew (J. Rodríguez-Romero et al., unpublished data) of grapevine plants collected in the spring and early summer of 2018 from the same regions has been characterized. The results of the three studies will show the great abundance and variety of ssRNA+ viruses, and the lower abundance, but not diversity, of dsRNA and ssRNA– viruses.

### Diversity of *B. cinerea* mycoviruses and geographical distribution.

A total of 248 *B. cinerea* isolates were collected from grape berries of different Italian and Spanish regions, cultured *in vitro*, and used to search for novel mycoviruses. Different mono- and multisegmented mycoviruses were identified, the majority of them with the ssRNA+ genome, several with dsRNA and ssRNA– genomes, and one with an ssDNA genome. The presence of mycoviruses with different classes of genomes in both countries and in most or all regions inside each country indicates that the geographical location has no or little influence on the distribution of mycoviruses based in the genome class. Mycoviruses with ssRNA and ssDNA genomes, with some exceptions, were present in both countries in several regions; however, other than the lower variability of dsRNA mycoviruses, most of them were found in both countries but only in one or a few pools, suggesting a restriction of the transfer of dsRNA mycoviruses between *B. cinerea* isolates. Alternatively, the presence of some mycoviruses in only one country, or in a single region inside a country, could also indicate a recent introduction of *B. cinerea* isolates infected with these mycoviruses that have limited its dispersion. There are studies on the distribution of mycoviruses infecting *B. cinerea* isolates from different regions of the world. Some of these have shown *B. cinerea* mycoviruses with a wide dispersion and prevalence, independent of the sampling time, host, or country. For instance, Botrytis cinerea mitovirus 1 has been found recurrently infecting *B. cinerea* isolates from different hosts (oilseed rape, pepper, and grapevine) in Spain, Italy, and China (23, 38, 82). Similarly, Botrytis virus F has been identified in surveys of *B. cinerea* isolated from different hosts (strawberry, grapevine, tomato, lettuce, and cucumber) in Spain, Italy, Israel, France, New Zealand, and Germany (23, 83–86). Nevertheless, there are some mycoviruses with limited distribution, such as, for instance, Botrytis cinerea mymonavirus 1, which has been found with low incidence in three distant regions of China (12) but has not been identified in our work. Interestingly, some dsRNA and ssRNA+ mycoviruses, previously identified as infecting other *Botrytis* species or other fungal genera, such as *Botrytis porri*, *Botryotinia fuckeliana* (a teleomorph of *B. cinerea*), or Sclerotinia sclerotiorum, were found in this work to infect *B. cinerea*, suggesting a horizontal transfer of these mycoviruses between coinfecting fungi, since all of them can be found in coinfections in some common plant hosts. In fact, *S. sclerotiorum* and *B. cinerea* are both necrotrophic fungi with very wide hosts ranges, whose genomes show high sequence identity and a similar arrangement of genes (87). This horizontal virus transfer has been reported previously by other authors between different genera of fungi, including *B. cinerea* (12, 58, 72), and has been considered, from a broader point of view, as a central aspect of RNA virus evolution (7).

### Unique bisegmented narna-like viruses.

ssRNA+ viruses include the largest and most diverse group of viruses infecting eukaryote (88). As expected, ssRNA+ viruses were the most abundant in our study; these included mitoviruses, narnaviruses, and botourmiaviruses. Mitoviruses and botourmiaviruses have been identified previously infecting *B. cinerea* (3, 23, 82), and here we increased the collection with the identification of several classical mitoviruses, and a great number of new botourmiaviruses, some of them constituting a new genus, that we propose to name *Penoulivirus*, inside the family *Botourmiaviridae* (47). However, until the present work, no narnaviruses were associated with *B. cinerea*. Narnaviruses are monosegmented ssRNA+ capsidless viruses that code for an RdRp in a sense orientation, and some of them also code for a hypothetical protein of similar size in an antisense orientation and replicate in the cytosol of host cells (89–91). Five novel narna-like viruses were identified for the first time infecting *B. cinerea* in our study, all of them coding for a single protein in a positive-sense orientation. BcNV4 was a classical narnavirus; however, the other four mycoviruses identified, named binarnaviruses, coded for a protein with identity to the RdRps of other narnaviruses included in the databases. However, none of the putative RdRps of these new mycoviruses contained the Gly-Asp-Asp (GDD) sequence found in motif VI of the RdRp palm domain, but they were associated with another narna-like viral sequence that coded for a hypothetical protein that contained this GDD sequence. Recently, Lin et al. (92) identified a narnavirus infecting *Magnaporthe oryzae* that was also missing the GDD triplet, and this indicated that this motif was also absent in the RdRp of other previously reported narnaviruses (21, 22, 93). We claim that these four novel binarnaviruses have a bisegmented genome. The presence of bisegmented narna-like virus (Matryoshka RNA virus 1 and Leptomonas seymouri Narna-like virus 1) has been previously reported (52, 53), and in these viruses one of the segments encoded a classical RdRp polymerase, and the second one encoded two small proteins. However, BcBNV HP did not show any identity with the small proteins of these two viruses. An interesting feature of these BcBNV HP is the conservation of the sequence “EVGDDR” containing the conserved triplet GDD of the RdRp. This finding raises the question of whether this GDD motif could somehow function in *trans*, through protein-protein interaction, as part of the catalytic site of the binarnavirus RdRp. Another hypothesis could be that the narnavirus RdRp lacking the GDD is completely functional but less efficient, as is the case of the dsRNA birnavirus RdRp, which is also lacking the GDD triplet or has repositioned this motif to different structural regions (94, 95). Further investigation of the function of these binarnaviral hypothetical proteins will be conducted to elucidate their role in the viral biological cycle, and the origin and evolution of these binarnaviruses will be explored. An encapsidated levivirus-like virus was likely the ancestor of naked mitoviruses and narnaviruses, and it has been hypothesized that a narnavirus was the ancestor of the segment coding for the RdRp of plant ourmiaviruses (16, 96). One hypothesis about the origin of these *B. cinerea* binarnaviruses is that they originated by the coinfection of two independently replicating narnaviruses in the same host, each of them coding for their own RdRp. These narnaviruses could have been evolved by accumulating mutations in their genomes but also deletions to the point that they had to combine and complement in *trans* their defective RdRps to have a correct functional one. However, the position of the RdRp motifs in both segments, at the end of segment 1 and the beginning of segment 2, suggests that more probably was an evolutionary transition from a nonsegmented virus to a bisegmented viral form. This genomic segmentation could have also been mediated by replication errors and recombination events, which implies the formation of defective RNA forms derived from the monosegmented genome. During the final review process of the present study, other groups reported similar multisegmented narna-like viruses with divided RdRps that infect the fungi *Oidiodendron maius* and Aspergillus fumigatus (97, 98), an observation which further supports our finding of *B. cinerea* binarnaviruses.

### Other novel positive-sense single-stranded RNA mycoviruses.

New endornaviruses, fusariviruses, and hypoviruses were identified infecting *B. cinerea* and that have similarities to previously described mycoviruses of the same genera associated with this fungus (41, 55). In addition, novel ssRNA+ as an alpha-like virus, an umbra-like virus, and as three novel mycoviruses related to members of the order *Tymovirales* were found for the first time associated with *B. cinerea*. The alpha-like mycovirus BcALV1 had a unique feature different from the already-described alpha-like viruses infecting fungi, which only encode a putative RdRp (8, 25, 27). BcALV1, in addition to encoding a protein with the conserved RdRp motifs, codes for other two small proteins of unknown function. BcFlV1 is the longest mycovirus in this group, with almost 14 kb encoding a single long protein, but it is in the same size range as other discovered flexiviruses infecting other genera of fungi (21, 99).

### Novel negative-sense single-stranded RNA mycoviruses.

The first viral sequence of ssRNA– coding for an RdRp associated with *B. cinerea* was Botrytis cinerea negative-stranded RNA virus 1 (BcNSRV1) (4). Since then, only one more ssRNA– mycovirus has been found to infect this fungus (12), indicating the low incidence of this class of mycoviruses in *B. cinerea*. Surprisingly, in the present work, 15 new ssRNA– mycoviruses were found, among them, six (BcNSRV3, -4, -5, and -7) were monosegmented and contained four ORFs, with the longest one coding for a putative mononegaviral RdRp. The phylogenetic analysis indicated that these six mycoviruses should be included in the family *Mymonaviridae* (62). Other seven mycoviruses have a single segment that encoded a protein with Bunya-RdRp motifs. However, there was no association with any other viral segment that can code for proteins involved in the viral life cycle, as either a nucleocapsid or a nonstructural protein, as was reported, for instance, for Penicillium roseopurpureum negative-sense RNA virus 1 (9). Similar mycoviruses with a single identified segment have been reported associated with other fungi (5). Five of these new mycoviruses (BcNSRV8, -9, -10 and -11 and BcOBV1) were allocated in a clade inside the group IV of negative-sense ssRNA viruses, order *Bunyavirales*, after the phylogenetic analysis of the RdRp sequences. Other two monosegmented ssRNA– mycoviruses, BcNSRV2 and BcNSRV6, also represent two novel mycoviruses infecting *B. cinerea*, phylogenetically related to phlebo-like viruses or other ssRNA– viruses (21, 27).

### A remarkable trisegmented ssRNA– mycovirus.

The most interesting ssRNA– mycovirus found in our study was BcBV1, a trisegmented mycovirus, each segment with a single ORF coding for putative Bunya-RdRp, a putative nucleocapsid and a hypothetical protein with identity to the putative movement proteins of ssRNA– cogu-like plant viruses (67). Phylogenetic analysis using the nucleocapsid protein placed BcBV1 with GaCLV1 in the same clade as the plant coguviruses. Alignment of the core domain of the 30K viral movement protein with the BcBV1 hypothetical protein showed high conservation in this region, suggesting that this hypothetical protein could be an ancient movement protein, most probably, not functional in BcBV1. Mycoviruses do not have movement proteins since they do not need them to survive inside their fungal hosts. However, two mycoviruses, Entoleuca phenui-like virus 1 and Lentinula edodes negative-strand virus 2, have been reported to encode hypothetical proteins with identity to plant coguvirus movement proteins and to the hypothetical protein of the tick-infecting LLV (96, 100). Interestingly, phylogenetic analysis showed that the RdRp of BcBV1 was grouped with GaCLV1, forming the proposed new genus *Bocivirus*, inside the family *Pheuniviridae*. Based on the phylogeny of the three proteins, the genomic segments of BcBV1 are apparently a mix of the three segments of GaCLV1, -2, and -3, with the RNA1 and RNA3 being more similar to the corresponding segments of GaCLV1 and the RNA2 having high identity to the GaCLV2 and -3 RNA2. Andika et al. (6) reported the discovery of a natural infection of the phytopathogenic fungus Rhizoctonia solani by a plant virus, cucumber mosaic virus, that was almost 100% identical to the one reported in the database, indicating that the fungus was recently infected by the plant virus. The identity between BcBV1 and GaCLV, other plant coguviruses, and LLV suggests a possible cross-kingdom event. Since BcBV1 still conserves a hypothetical protein similar to a putative movement protein, the most probable scenario could be that an ancient plant virus, coinfecting grapevine with *B. cinerea*, was transferred from the grapevine plant to the fungus, and the resulting mycovirus BcBV1 is the product of the evolution of the ancient plant virus inside the fungus.

### Novel dsRNA mycoviruses.

A few dsRNA mycoviruses have been reported to infect *B. cinerea* (43, 44, 71). The identification of new partitiviruses, botybirnaviruses, and victoriviruses here increased this collection of *B. cinerea* dsRNA mycoviruses. In addition, two novel bisegmented mycoviruses were found, BcMyV3 and -5, that do not have an ORF coding for a coat protein and therefore could be naked dsRNA mycoviruses. Both are phylogenetically close to members of the family *Partitiviridae*, but in different groups, probably representing two different genera inside a novel family of dsRNA mycoviruses. Interestingly, we have also found a novel class of tetrasegmented dsRNA mycovirus infecting *B. cinerea*, with high identity to Botrytis cinerea RNA virus 2 in three segments and to RnQV1 in the remaining segment. Botrytis cinerea RNA virus 2, with a trisegmented genome, was probably missing the fourth genomic segment that was found in this study to obtain the complete genome of BcMyV4. This new *B. cinerea* mycovirus, BcMyV4, represents a novel member of the family *Quadriviridae*.

### A novel single-stranded DNA mycovirus.

To date, only two ssDNA mycoviruses have been associated with fungi (13, 14), and we have reported here the characterization of the first ssDNA mycovirus infecting *B. cinerea*, BcssDV1. This new virus showed identity to Bemisia-associated genomovirus Nfo and to the trisegmented ssDNA mycovirus, Fusarium graminearum gemytripvirus (14). Phylogenetically, BcssDV1 was associated with Fusarium graminearum gemytripvirus, and other members of the genus *Gemykrogvirus* inside the family *Genomoviridae* (101), but they are clearly separated from the genus *Gemykibivirus*, which includes Bemisia-associated genomovirus Nfo, and from the genus *Gemycircularvirus*, which includes the other ssDNA mycovirus infecting *S. sclerotiorum* (13). Sclerotinia sclerotiorum hypovirulence-associated DNA virus is a monopartite mycovirus coding for two proteins, and Fusarium graminearum gemytripvirus is a tripartite mycovirus. In the case of BcssDV1, a single segment was characterized encoding the replication associated protein. However, segments coding for other proteins of variable sizes with no identity to any other protein in the database were also found. Additional analyses are already being conducted to fully characterize this new putative multisegmented ssDNA virus infecting *B. cinerea*.

### Conclusions.

This study reveals the mycovirome composition of one of the most economically important plant-pathogenic fungi, *B. cinerea*, through viral metagenomics analysis. The results obtained here have expanded our knowledge of mycoviral diversity, horizontal transfers, and putative cross-kingdom events. These findings helped us to explore the evolutionary history of mycoviruses and to increase the collection of viruses infecting *B. cinerea* that could be used in biocontrol strategies.

## MATERIALS AND METHODS

### Sample collection of gray mold in grapevine.

The 248 samples used in this study were collected during the summer of 2018 in Italy and Spain from several varieties of grapevine plants infected with *B. cinerea*. A total of 150 Spanish samples were collected in four of the main grapevine-producing areas of Spain: Jerez in the south, Ribera de Duero in the center/northwest, La Rioja in the north, and Penedés in the northeast (see Fig. S1A in the supplemental material). Samples were isolated from different grapevine cultivars as for instance Tempranillo, Palomino, or Macabeu. A total of 98 Italian samples were collected mainly in the north of Italy, covering different areas, from Piedmont to Veneto. This also reflected a wide range of grapevine varieties as Dolcetto, Barbera, Groppello, Cabernet, Merlot, Chardonnay, and many others. A smaller proportion of the samples was also collected from center and south Italy, in order to cover the country latitudes (see Fig. S1B). The number of sequenced pools determined by high-throughput sequencing, the regions, the locations, the total numbers of samples, and the samples per pool are represented in Fig. S1. After collection, the infected grapes were immediately stored at 4°C in a moist bag until fungal isolation in potato dextrose agar (PDA). The mycelium of each fungal sample was isolated from the infected grapes with a sterile tip and plated in a PDA plate that was incubated at 23°C in darkness until the mycelia were completely developed. Some samples were reisolated several times to avoid other fungal or bacterial contamination associated with field samples. Each isolate was cultured in a plate with potato dextrose broth (PDB) for 3 days. The mycelia were collected and dried using Miracloth paper, frozen in liquid nitrogen, and maintained at −80°C until total RNA extraction. For determination of the fungal species, specific primers (Bc3F, GCTGTAATTTCAATGTGCAGAATCC; Bc3R, GGAGCAACAATTAATCGCATTTC) for the species “*cinerea*” were used in a qPCR with as the template total fungal DNA. The total fungal DNA was obtained by scratching the surfaces of mycelium plates with a toothpick or a 10-μl tip and boiling it in 20 μl of sterile water.

### Total RNA extraction of *Botrytis cinerea* isolates.

For total RNA extraction, 100 mg of mycelia were resuspended in lysis buffer (Spectrum Plant Total RNA kit; Sigma-Aldrich); in the same tube, 0.5 ml of glass beads (0.1 mm) was added, and the samples were mixed in a beat beater (Qiagen TissueLyser II) at maximum speed for 20 to 30 s and immediately placed in ice. After total RNA extraction according to the Spectrum Plant Total RNA kit instructions (Sigma-Aldrich), the samples were measured using a UV spectrophotometer to determine the concentration, and electrophoresis in agarose gels indicated the quality. Only samples with a minimum of 40 ng/ml were included in pools for high-throughput sequencing (i.e., the 248 samples described above). The high-quality samples were combined in each pool, resulting in 17 pools from Spain and 12 pools from Italy.

### RNA next-generation sequencing and bioinformatics pipelines.

RNA samples pools were sent to Macrogen (Seoul, Republic of Korea) for library preparation (Illumina TrueSeq) and sequence analysis with an Illumina NovaSeq 6000. For each library, >100 million pair-ended reads, 150 bases long, were retrieved. To assemble and identify the viruses *in silico*, the pipeline was divided into four steps: (i) cleaning, (ii) *de novo* assembly, (iii) viral sequence identification, and (iv) mapping. Read cleaning was performed using Bbtools (102) by removing adapters, artifacts, short reads, and ribosomal sequences. The cleaning step output was used as input for the *de novo* assembly performed with Trinity software (v2.3.2) (103). The third step used a Blast approach to identify viral sequences. First, a custom viral database was queried with the assembled contigs by using an NCBI Blast toolkit (v2.8). The results were manually inspected, and reliable viral sequences, based on the identity percentage, alignment length, and query length, were selected for the following analysis steps. The candidate contigs were Blast evaluated against NCBInr (release October 2019) using DIAMOND software (104). These second Blast results were used to discriminate between real viruses and integrated viruses or host sequences. Selected virus sequences were mapped with clean reads using bwa (105) transformed with SAMtools (106) and then visualized with Tablet software (112). For ORF prediction, Expasy Translator and Open Reading Frame Finder were used with default parameters. BLASTP was used to confirm the identity of the translated proteins by searching again in the database. All viral sequences with a length of >1,000 nt, close to the size of the reference genome, and with a complete coding sequence or missing some amino acids from the amino- or carboxy-terminal end in the coded proteins were submitted to GenBank. All assembled contigs of each pool were Blast evaluated against the NCBI database using DIAMOND software. The resulting “.daa” files were analyzed with MEGAN6 to verify the taxonomic variety of the samples and the abundance of contigs pertaining to *B. cinerea*.

### Detection of mycoviruses infecting *B. cinerea in vivo*.

Total RNA from each of the 29 pools was used as the template for cDNA synthesis with a high-capacity cDNA reverse transcription kit (Applied Biosystems). A dilution of the synthetized cDNA was used as the template in a qPCR (FastStart Universal SYBR green Master Rox; Roche) with specific primers designed for the detection of 47 mycoviral segments of 36 *B. cinerea* mycoviruses (see Table S2). Total RNA extracted from individual isolates of several pools (BCI1, BCS14, and BCS15), and the cDNA obtained was also used as the template for the detection of three mycoviruses. qPCR primers were used for the detection of Botrytis cinerea binarnavirus 2 segments 1 and 2 by qPCR in *B. cinerea* isolates of the pool BCS14. qPCR primers or new primers, specifically designed for conventional PCR, were used for the detection of Botrytis cinerea bocivirus 1 RNA1, -2, and -3 in *B. cinerea* samples of pool BCS15 (see Table S2). To detect BcBV1 segments by conventional RT-PCR, SuperScript IV (Thermo Fisher Scientific) was used for retrotranscription, and the PCR was performed using *Taq* CloneAmp HiFi PCR (TaKaRa) for maximum efficiency. To detect Botrytis cinerea ssDNA virus 1, extracted DNA (DNeasy plant minikit; Qiagen) from *B. cinerea* isolates of pools BCI1 and BCS15 was used as the template in a rolling-circle amplification assay (TempliPhi amplification kit) to enrich the circular viral DNA. Dilutions of resulting product were used as the template in a qPCR and in a conventional PCR with specific primers (see Table S2). The resulting amplicons of Botrytis cinerea ssDNA virus 1 and Botrytis cinerea bocivirus 1 were subjected to Sanger sequencing to verify their detection. In addition, the BcssDV1 DNA extracted from several samples was used as the template to amplify part of the genome by conventional PCR using virssDNArep forward and reverse primers (see Table S2). The amplified PCR product was sequenced.

To explore the possibility of an ambisense segment in BcBV1 (RNA2/RNA3) instead two separated segments, the cDNA was also used as the template for two PCR amplification of 499- and 534-nt fragments by combining BcBV1_RNA2_RACE_3′out Forward/BcBV1_RNA3_RACE_5′out Reverse and BcBV1_RNA3_RACE_3′out Forward/BcBV1_RNA2_RACE_5′out Reverse, respectively (see Table S2). As a positive control, the cDNA was used as the template for the amplification of specific fragments of 303 nt of the RNA2 (BcBV1_RNA2_Fw and Rev) and of 286 nt of the RNA3 (BcBV1_RNA3_Fw and Rev) (see Table S2).

To explore the possibility of a monosegmented genome of BcBNV2 instead a bisegmented genome, the cDNA was used as the template for two PCR amplification by combining BcBNV2_pro_Fw_rep/BcBNV2_HP_RACE5_gs_in (1.4 kb) and BcBNV2_pro_Fw_HP/BcBNV2_rep_RACE5_gs_in (1.5 kb) (see Table S2). As positive controls, the cDNA was used as the template for the amplification of specific fragments of 1 kb for segments 1 (BcBNV2_rep_det_Fw and BcBNV2_rep_det_Rev) and 2 (BcBNV2_rep_det_Fw and BcBNV2_rep_det_Rev) (see Table S2).

To ensure that BcBNV2 has a bisegmented genome and BcBV1 has a trisegmented genome and that they are transmitted vertically via spores, the independent segments of both mycoviruses were detected in single spore isolates. Field isolates BC93 (pool BCS14) and BC118 (pool BCS15) were cultured for 1 day in PDA plates. Spores were collected in PDB, and 100 μl of dilutions of ∼50 spores/ml were grown in water agar (2%). After 2 days, single-spore colonies were isolated and cultured in PDA plates to obtain BC93M and BC118M isolates. The total RNA was obtained from 1 g of mycelia of single-spore isolates using TRIzol (NZYTech) according to the manufacturer’s instructions. The cDNA was synthesized from 1 g of total RNA with an NZY first-strand cDNA synthesis kit, according to the manufacturer’s instructions, using specific primers. The cDNA was used as the template for PCR amplification with Supreme NZYTaq II 2× Green Master Mix. Amplification of specific fragments (∼0.5 kb) of BcBNV2 segments was carried out with the primers BcBNV2_pro_Fw_rep and BcBNV2_pro_Rev_rep to amplify segment 1 and the primers BcBNV2_pro_Fw_HP and BcBNV2_pro_Rev_HP for segment 2 (see Table S2). Amplification of specific fragments (∼0.3 kb) of BcBV1 segments was performed with the primers BcBV1_RNA1_Fw and BcBV1_RNA1_Rev for segment 1, the primers BcBV1_RNA2_Fw and BcBV1_RNA2_Rev for segment 2, and the primers BcBV1_RNA3_Fw and BcBV1_RNA3_Rev for segment 3 (see Table S2).

### Determination of the 5′ and 3′ of Botrytis cinerea binarnavirus 2 and Botrytis cinerea bocivirus 1 genomes.

BcBNV2 segments 1 and 2 and BcBV1 RNA1, -2, and -3 ends were determined using a SMARTerRACE 5′/3′ kit (TaKaRa) for 5′ ends and rapid amplification of cDNA ends (RLM-RACE kit; Thermo Fisher) for 3′ ends, according to the manufacturer’s instructions. Briefly, for the 5′ end cDNA was synthesized with SMARTer II A oligonucleotide (5′-AAGCAGTGGTATCAACGCAGAGTACGCGGG-3′) using SMARTScribe reverse transcriptase (TaKaRa). The cDNA was used as the template for an outer PCR with universal primer (5′-CTAATACGACTCACTATAGGGCAAGCAGTGGTATCAACGCAGAGT-3′) and specific viral primers (primers “out”; see Table S2). Outer amplicons were used as the templates for an inner PCR using UPM short (5′-CTAATACGACTCACTATAGGGC-3′) and specific viral primers (primers “in”; see Table S2). For the 3′ end, RNA was polyadenylated with PolyA polymerase (TaKaRa) and cDNA was synthesized with 3′ RACE adapter (5′-GCGAGCACAGAATTAATACGACTCACTATAGGT12VN-3′). Finally, cDNA was used as the template for an outer PCR using amplified 3′ RACE outer primer (5′-GCGAGCACAGAATTAATACGACT-3′) and specific viral primers (primers “out”; see Table S2). Outer amplicons were used as the templates for an inner PCR using 3′ RACE inner primer (5′-CGCGGATCCGAATTAATACGACTCACTATAGG-3′) and specific viral primers (primers “in”; see Table S2). PCR products were cloned and sequenced.

### Phylogenetic analyses.

RNA-dependent RNA polymerase proteins from all identified viruses and closest homologues from the National Center for Biotechnology Information (NCBI) were aligned using the online version of Clustal Omega software with default parameters (107) or MUSCLE implemented in MEGAX (108). On the basis of the aligned amino acid sequences, the trees were computed by MEGAX (108) or submitted to IQ-TREE software (109) to produce accurate phylogenetic trees under a maximum-likelihood model (default parameters). The percentage of replicate trees in which the associated taxa clustered together in a bootstrap test (1,000 replicates) were shown next to the branches (110). The tree was drawn to scale, with branch lengths in the same units as those of the evolutionary distances used to infer the phylogenetic tree. The evolutionary distances were computed using the Dayhoff matrix-based method (111) and are in the units of the number of amino acid substitutions per site. All positions with <50% site coverage were eliminated, i.e., fewer than 50% alignment gaps, missing data, and ambiguous bases were allowed at any position (partial deletion option). The accession numbers of the proteins and the corresponding virus names are displayed on the trees.

### Data availability.

All the raw sequencing reads have been stored in SRA database: BioProject accession no. PRJNA632510, BioSample accession numbers SAMN14911182 to SAMN14911210, and SRA accession numbers SRX8335942 to SRX8335970.
