# Supplementary material for: Novel Mycoviruses Discovered in the Mycovirome of a Necrotrophic Fungus
Source: mBio. 2021 May 11;12(3):e03705-20. doi: 10.1128/mBio.03705-20 (PMC8262958; doi:10.1128/mBio.03705-20)
Supplement: FIG S1 [file mbio.03705-20-sf001.pptx]

## Slide 1
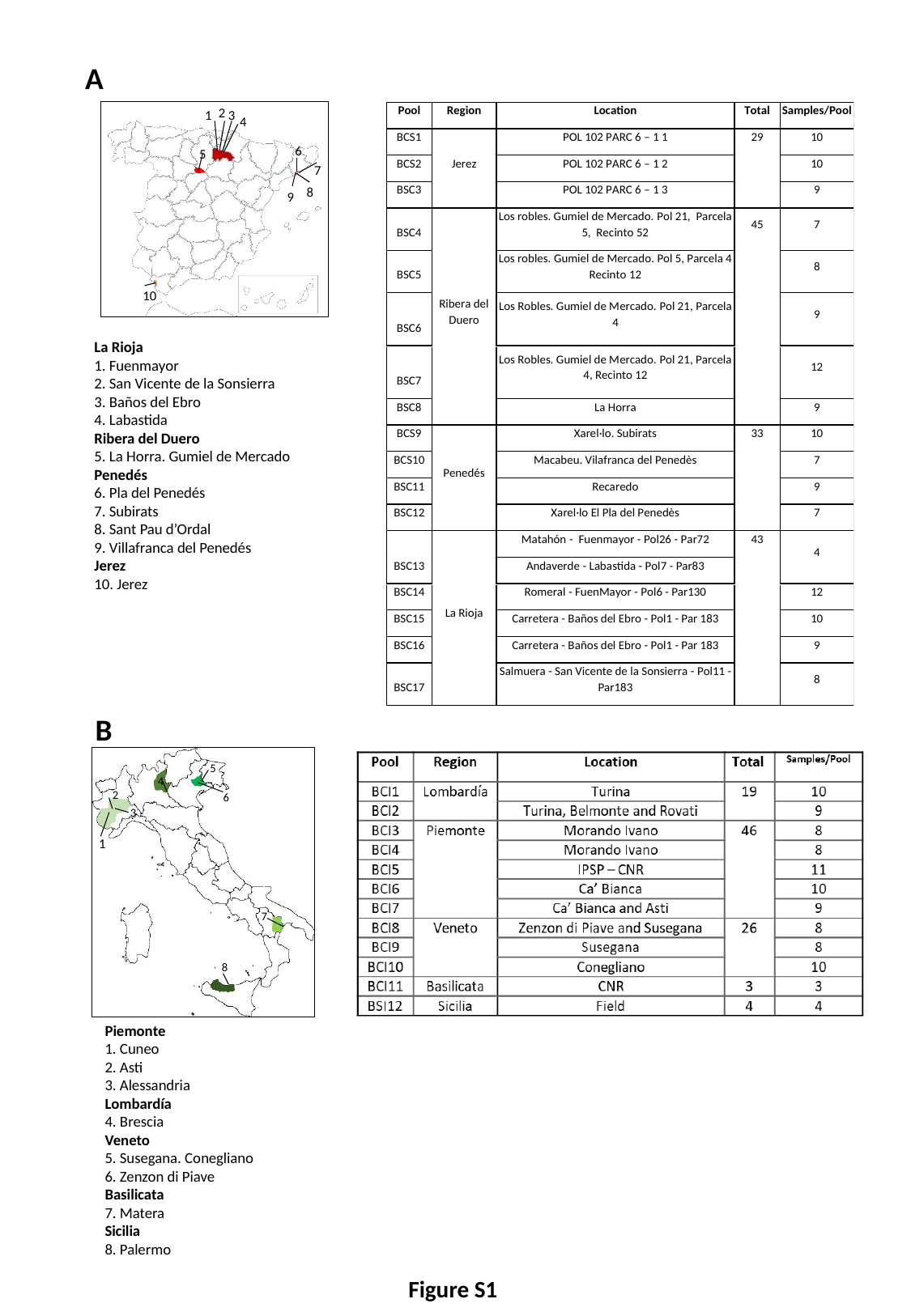

A
2
1
3
4
6
5
7
8
9
10
La Rioja
1. Fuenmayor
2. San Vicente de la Sonsierra
3. Baños del Ebro
4. Labastida
Ribera del Duero
5. La Horra. Gumiel de Mercado
Penedés
6. Pla del Penedés
7. Subirats
8. Sant Pau d’Ordal
9. Villafranca del Penedés
Jerez
10. Jerez
B
5
4
2
6
3
1
7
8
Piemonte
1. Cuneo
2. Asti
3. Alessandria
Lombardía
4. Brescia
Veneto
5. Susegana. Conegliano
6. Zenzon di Piave
Basilicata
7. Matera
Sicilia
8. Palermo
Figure S1

## Slide 2
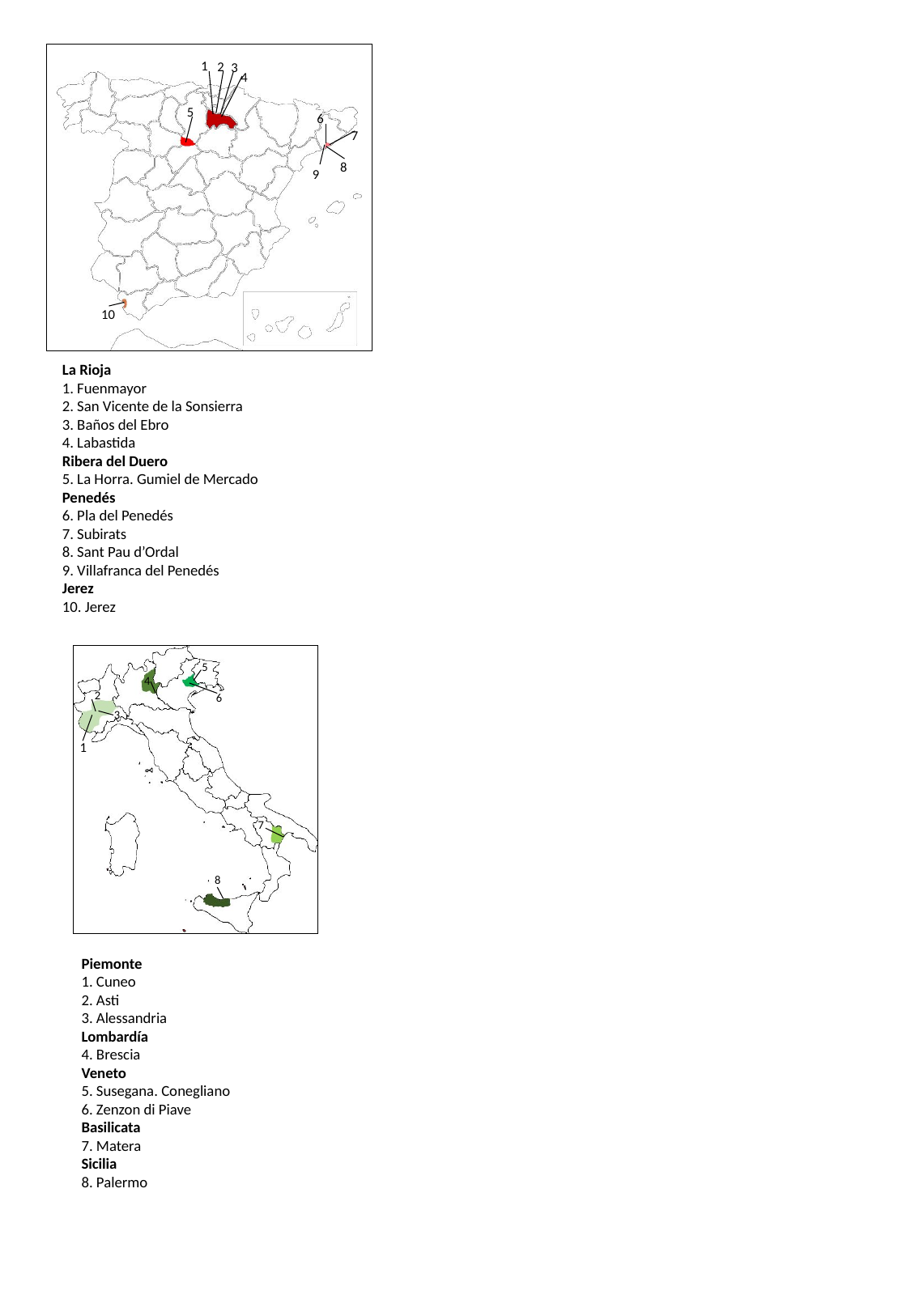

1
2
3
4
5
6
7
8
9
10
La Rioja
1. Fuenmayor
2. San Vicente de la Sonsierra
3. Baños del Ebro
4. Labastida
Ribera del Duero
5. La Horra. Gumiel de Mercado
Penedés
6. Pla del Penedés
7. Subirats
8. Sant Pau d’Ordal
9. Villafranca del Penedés
Jerez
10. Jerez
5
4
2
6
3
1
7
8
Piemonte
1. Cuneo
2. Asti
3. Alessandria
Lombardía
4. Brescia
Veneto
5. Susegana. Conegliano
6. Zenzon di Piave
Basilicata
7. Matera
Sicilia
8. Palermo
