# Supplementary figures and images for: Novel Mycoviruses Discovered in the Mycovirome of a Necrotrophic Fungus
Source: mBio. 2021 May 11;12(3):e03705-20. doi: 10.1128/mBio.03705-20 (PMC8262958; doi:10.1128/mBio.03705-20)

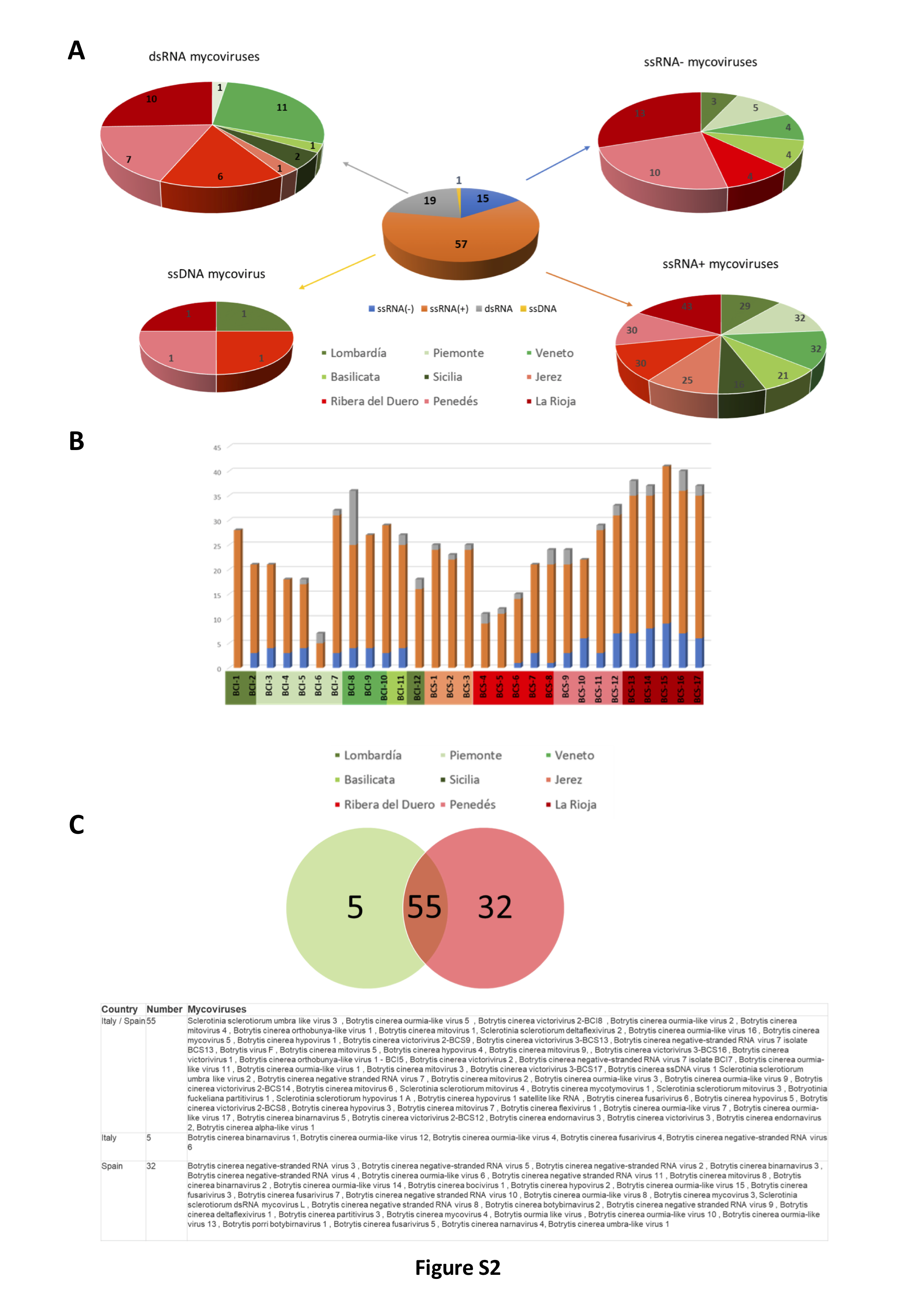

Supplement: FIG S2 [file mbio.03705-20-sf002.tif]

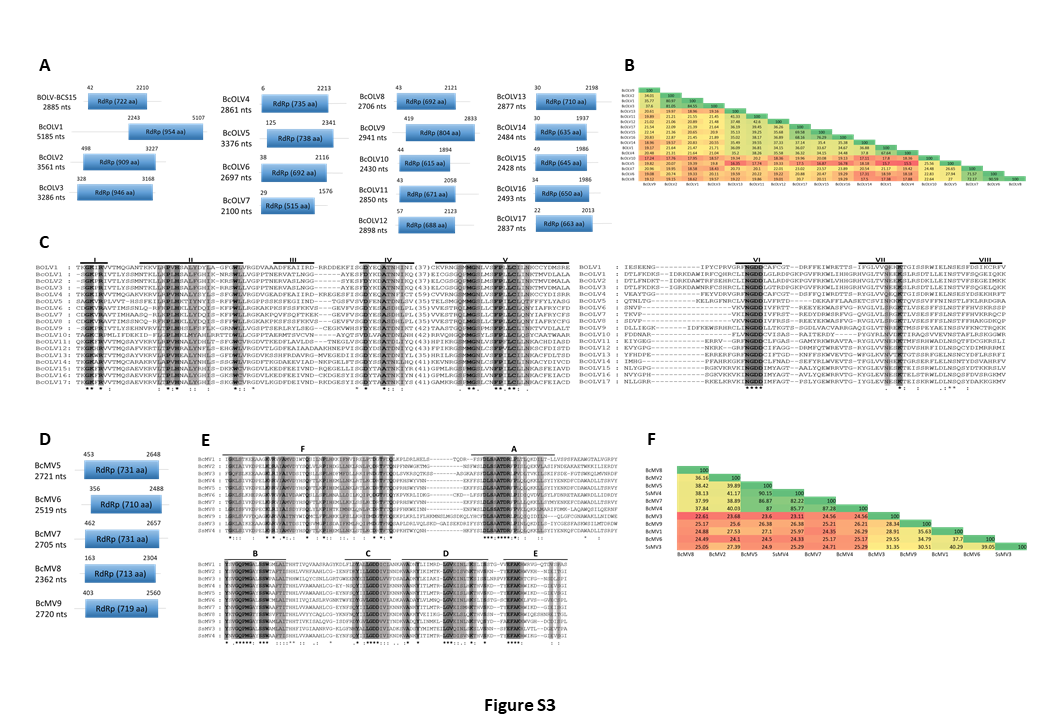

Supplement: FIG S3 [file mbio.03705-20-sf003.tif]

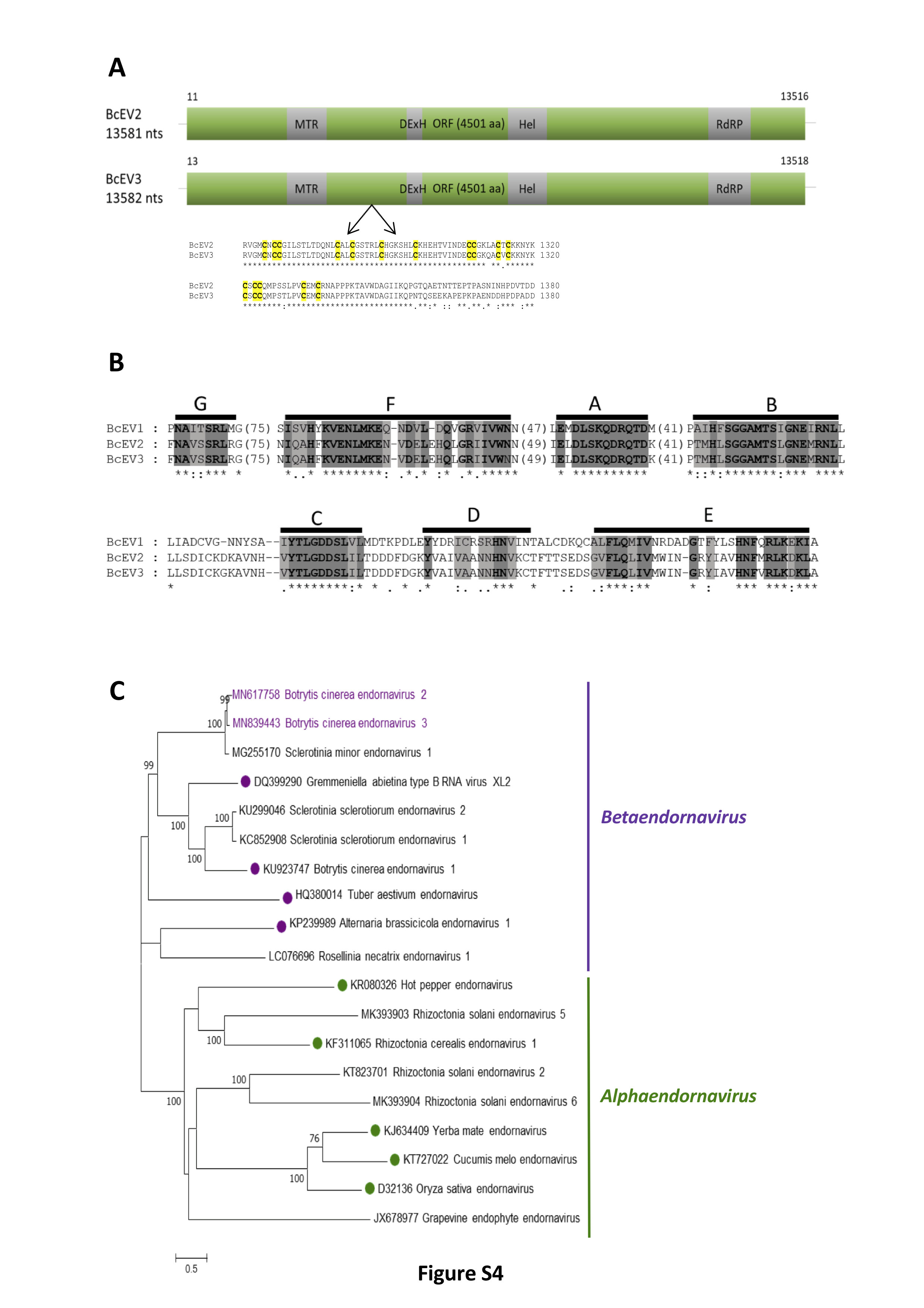

Supplement: FIG S4 [file mbio.03705-20-sf004.tif]

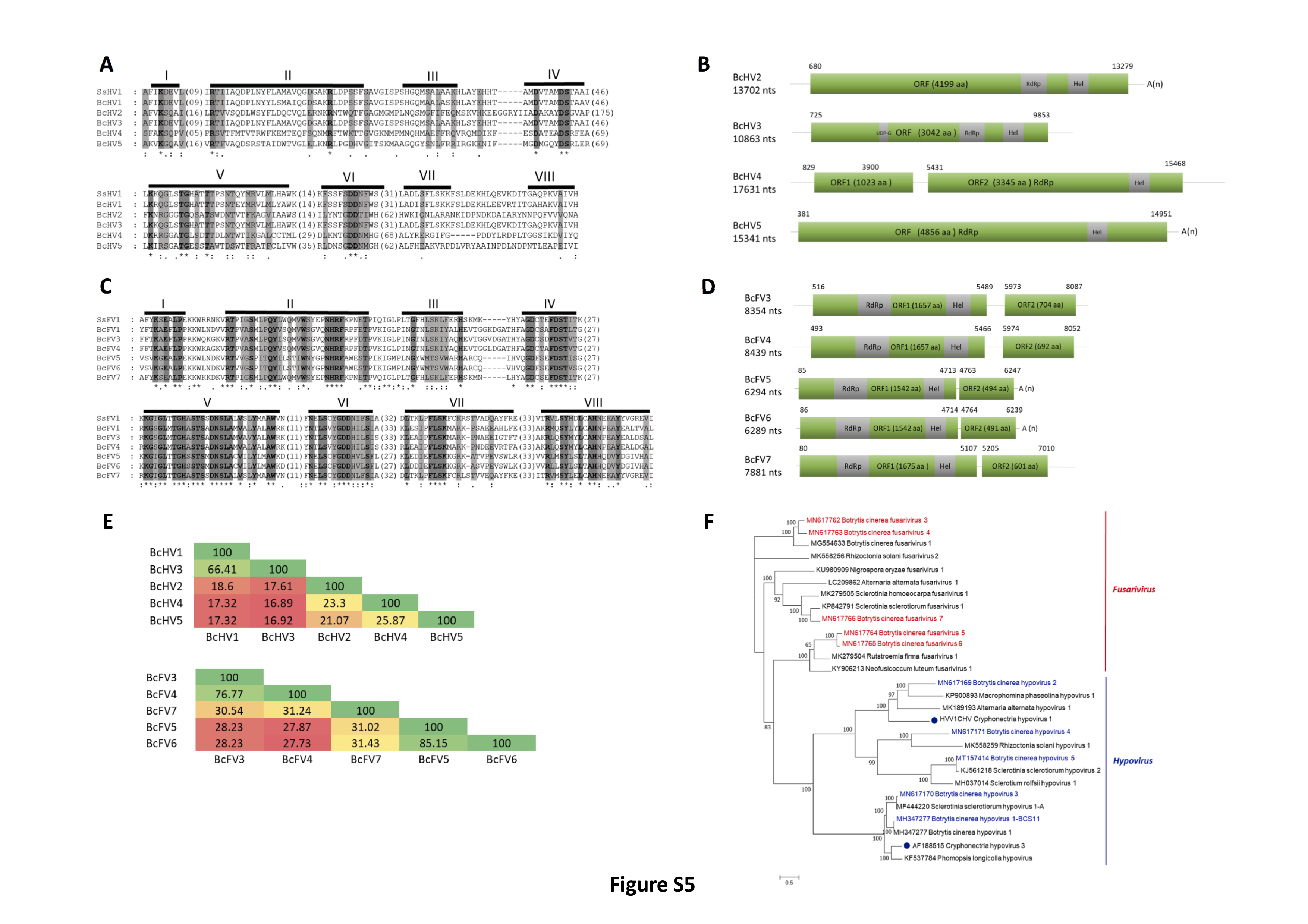

Supplement: FIG S5 [file mbio.03705-20-sf005.tif]

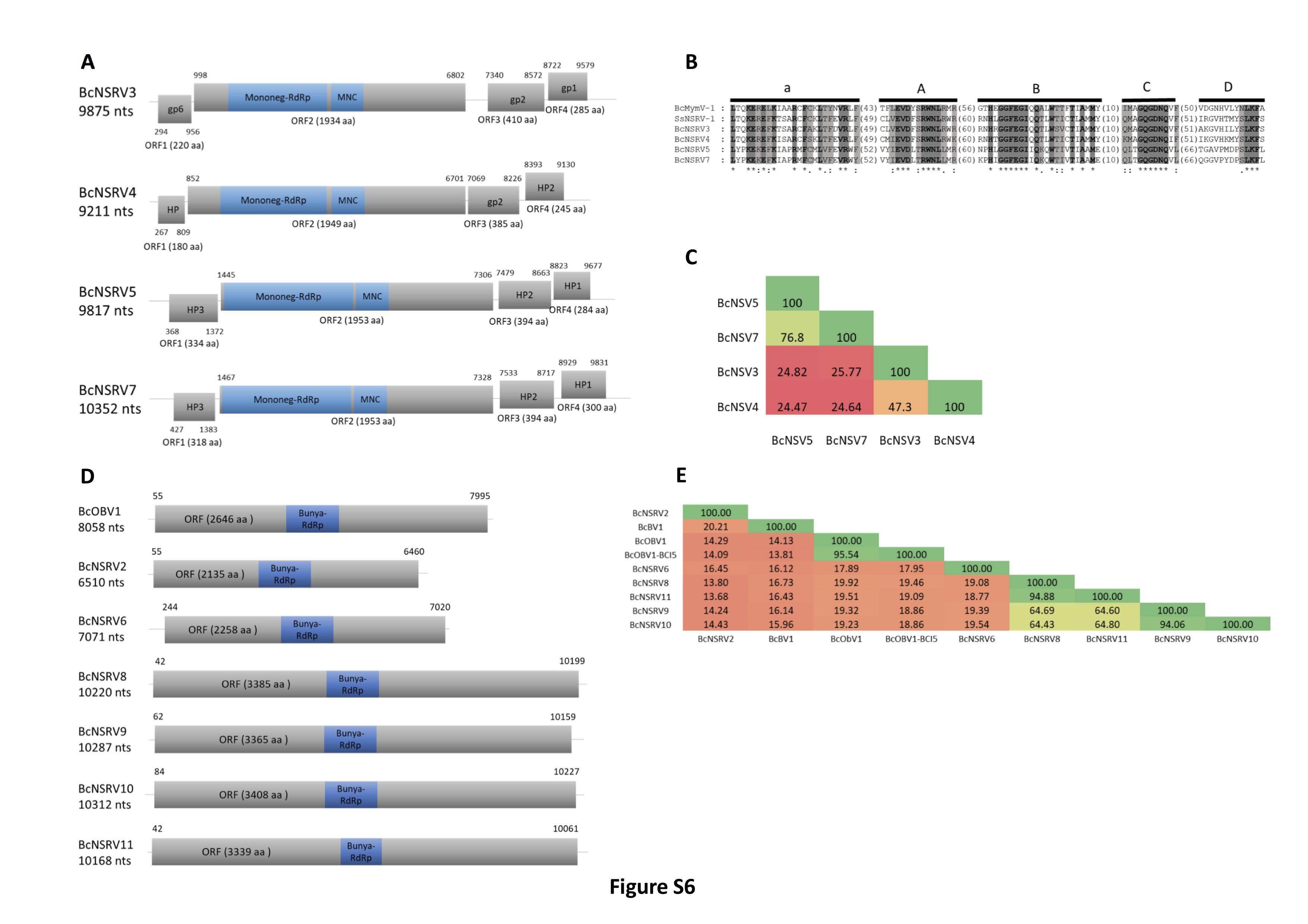

Supplement: FIG S6 [file mbio.03705-20-sf006.tif]

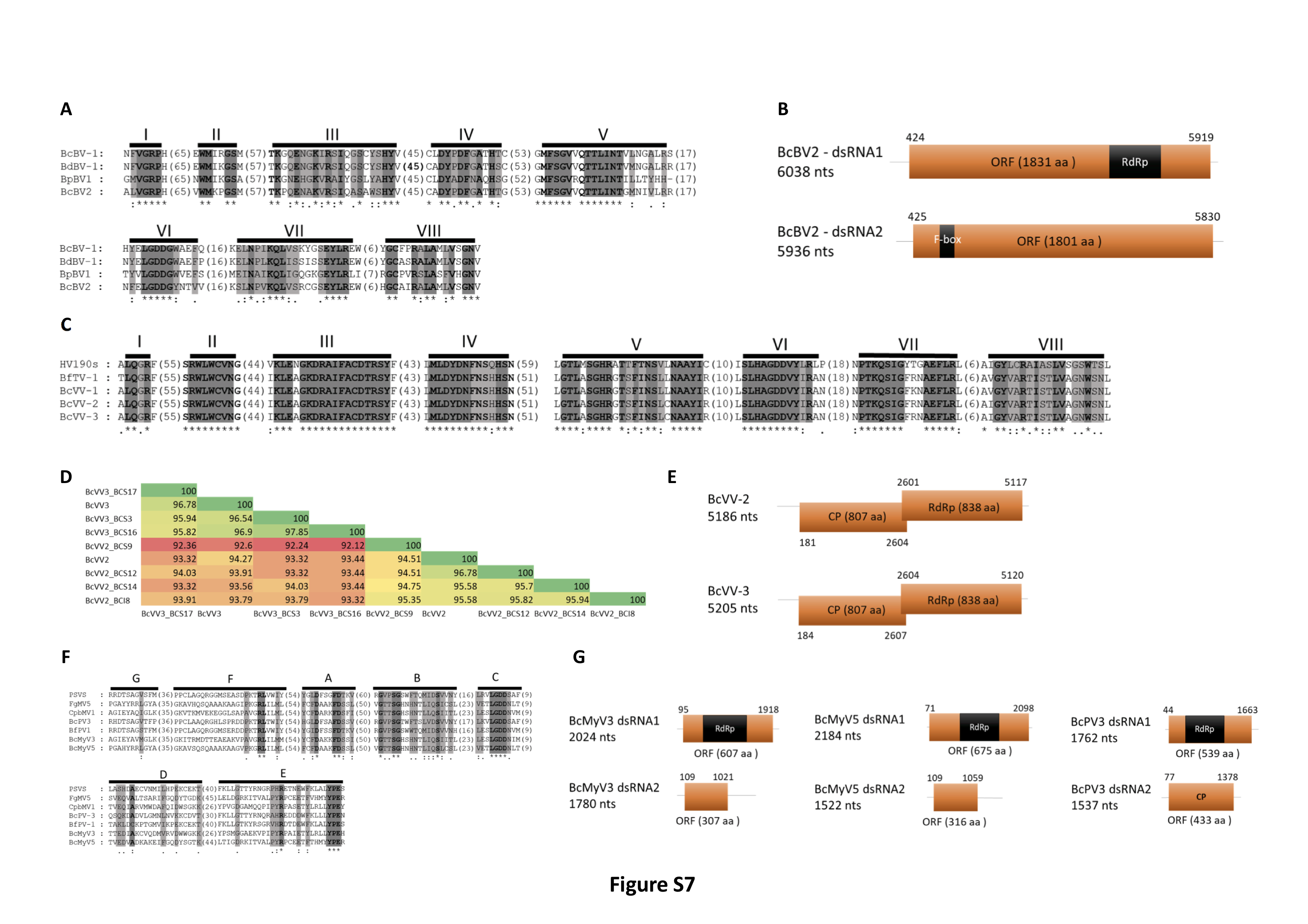

Supplement: FIG S7 [file mbio.03705-20-sf007.tif]
