# Supplementary material for: Novel Mycoviruses Discovered in the Mycovirome of a Necrotrophic Fungus
Source: mBio. 2021 May 11;12(3):e03705-20. doi: 10.1128/mBio.03705-20 (PMC8262958; doi:10.1128/mBio.03705-20)
Supplement: FIG S8 [file mbio.03705-20-sf008.docx]

**Figure S8**

**>BcssDV1s len=1426**

AAAAAATAATTAATTCATTTCATGTCATGTGACTTCGTCCCTCTCTGTCAGAATATTTGCTATAAAGTGTGGTCATGTCCAAACGCAAATGAAGAGTAAAATTTTAAGACAACAAGTTGTCCTACTCTTCATTTGCTGCTGCCTTAACGTTTTTTGTGGAAT**ATG**CCTAAACGAGCCTATAGCGAAAGGGATGAAACCCCTGGAAGCATCGCCGGTTTTATCGATGATCAGGCGGAGCTTTCAGGCTCCGATGTGGCTGAAGACCCGGAGGACGCCGATATCCAAGCCCCAAAGAGGCGCAAACATACCGACAGGTTTCGGATTAGGGCACAACGCATAATGTTGACCTATGCCCAGATAGATGACACGTTTGATAGAGAGAAGTTCGGACCATGGCTGAAGGAAAAGTGTGGAGCTCTGACTATAAGAGTAGCTCTCGAGGAGCACAAAGAGACCGGTGGTCTACACATCCACGCCTACGTCGAAGCACCAACACAATTCACGATCAACTCTGCCGACTTTCTCGATTACTGCGGTCACCACCCCAACATACTGCCAATCAGAGTCACGTTTTACAAAACTTGGGATTACGTAGGCAAAGACGGAGACATCATCTTTGAAGAAGGGCCGCCACCACCACGACCTGCAGAAAAATCAGGATCAGCTGCCGTGTGGACTGCCATCGTAATGGCAGCCAATACCGTCGACGAATTTTTGGAGGAGGCCTTCAAATCAAGGCCTGGGGACTTGATCAAAAACTTCACCCAATTCAAAGCCTTCGCCGAGTGGAGATACAAGCCAAAGGGGTTGACATACGTGTCACCAGAGATCGTGTGTCACATGGAGGACTACCCACAACTTGAGCAGTGGGTATTGGAGAACTTACGAGGAGGACTGCCAGCGGGTGCCCGCAAGCAGTCATTGGTCATGTGGGGTGACACCAGACTGGGCAAAACACTTTGGGCTAGGTCGTTGGGCAAGCATGCATACTTCCCAGGCATGTTTATGTTGGACGGTTTCAACGAGGAGGGCTGCGAGTATGCCATCTTCGATGACATAATCGAAGGATTCAAAGGCATCGCCAGCTACAAGGGGTGGTTTGGGTCTCAACATGAGGTCGTCGTCACCGACAAGTACCGTATGAAGCAGAGAATCACGTGGGGAAGACCTTCGGTCTTCATCAGCAACACTGATCCACGTGACGATTTGCCTAGAGATCAAGTCAAATGGTTGGAGGGAAATTGTGTTTTTGTGCATATTGATAAAGTACTATGCGAGCCGTATGTAGGTGAATCACCAACA**TAG**GCGCCCAAAAAAATAATTAATTCATTTCATGTCATGTGACTTCGTCCCTCTCTGTCAGAATATTTGCTATAAAGTGTGGTCATGTCCAAACGCAAATGAAGAGTAAAATTTTAAGACAACA

**>BcssDV1l len=1694**

ACAACAAGTTGTCCTACTCTTCATTTGCTGCTGCCTTAACGTTTTTTGTGGAAT**ATG**CCTAAACGAGCCTATAGCGAAAGGGATGAAACCCCTGGAAGCATCGCTGGTTTTATCGATGATCAGGCGGAGCTTTCAGGCTCCGATGTGGCTGAAGACCCGGAAGACGCCGATATCCAAGCCCCAAAGAGGCGCAAACAGTGAGTCACTGCCTATTTTTTTTAGACGTGCAACAAAACAATTACAGCACGTGTACACCCCGTGATACGCTATAAGAGCCTGATCATTCAGGCTGTGTCCGCTCGATCCCTCCCTCGTGCCTCGGGGGGAGCCCATCTCGCTCAAGGACCTATCTTTGGCTAGGGTAGGCTATTACAGGGCCTCGTCCTCCGAGCTCCCCTCCATCGTCGTTCCTCCTCAGGGGGTCCCCCAGGCCGCTCGAGGATTCTCACGGCCCACATCACTATAACATCACTATATTTACAGCGCCTTACAGGCTATCACTATAACACCCTGAAGTTTCAGGGTTACACCGCTATATTTACAGGCTATTACAGGCTTTAAATCGCTAACACTAACAGTACCGACAGGTTTCGGATTAGGGCACAACGCATA**ATG**TTGACCTATGCCCAGATAGATGACACGTTTGATAGAAAGAAGTTCGGACCATGGCTGAAGGAAAAGTGTGGAGCTCTGACTATAAGAGTAGCTCTCGAGGAGCACAAAGAGACCGGTGGTCTACACATCCACGCCTACGTCGAAGCACCAACACAATTCACGATCAACTCTGCCGACTTTCTCGATTACTGCGGTCACCACCCCAACATACTGCCAATCAGAGTCACGTTTTACAAAACTTGGGATTACGTAGGCAAAGACGGAGACATCATCTTTGAAGAAGGGCCGCCACCACCACGACCTGCAGAAAAATCAGGATCAGCTGCCGTGTGGACTGCCATCGTAATGGCAGCCAATACCGTCGACGAATTTTTGGAGGAGGCCTTGAAATCAAGGCCTGGGAACTTGATCAAAAACTTCACCCAATTCAAAGCCTTCGCCGAGTGGAGATACAAGCCAAAGGGGTTGACATACGTGTCACCAGAGATCGTATGTCACATGGAGGACTACCCACAACTTGAGCAGTGGGTATTGGAGAACTTACGTGGAGGACTGCCAGCGGGTGCCCGCAAGCAGTCATTGGTCATGTGGGGTGACACCAGACTGGGAAAAACACTTTGGGCTAGGTCGTTGGGCAAGCATGCATACTTCCCAGGCATGTTTATGTTGGACGGTTTCAACGAGGAGGGCTGCGAGTATGCCATCTTCGATGACATAATCGAAGGATTCAAAGGCATCGCCAGCTACAAGGGGTGGTTTGGGTCTCAACATGAGGTCGTCGTCACCGACAAGTACCGTATGAAGCAGAGAATCACGTGGGGAAGACCTTCGGTCTTCATCAGCAACACTGATCCACGTGACGATTTGCCTAGAGATCAAGTCAAATGGTTGGAGGGAAATTGTGTTTTTGTGCATGTTGATAAAGTACTATGCGAGCCGTATGTAGGTGAATCACCAACA**TAG**GCGCCCAAAAAAAATAATTAATTCATTTCATGTCATGTGACTTCGTCCCTCTCTGTCAGAATATTTGCTATAAAGTGTGGTCATGTCCAAACGCAAATGAAGAGTAAAATTTTAAG

**Alignment BcssDV1s (1426 nt) vs BcssDV1l (1694 nt)**

10 20 30 40 50 60

BcssDV ACAACAAGTTGTCCTACTCTTCATTTGCTGCTGCCTTAACGTTTTTTGTGGAATATGCCT

::::::::::::::::::::::::::::::::::::::::::::::::::::::::::::

BcssDV ACAACAAGTTGTCCTACTCTTCATTTGCTGCTGCCTTAACGTTTTTTGTGGAATATGCCT

10 20 30 40 50 60

70 80 90 100 110 120

BcssDV AAACGAGCCTATAGCGAAAGGGATGAAACCCCTGGAAGCATCGCCGGTTTTATCGATGAT

:::::::::::::::::::::::::::::::::::::::::::: :::::::::::::::

BcssDV AAACGAGCCTATAGCGAAAGGGATGAAACCCCTGGAAGCATCGCTGGTTTTATCGATGAT

70 80 90 100 110 120

130 140 150 160 170 180

BcssDV CAGGCGGAGCTTTCAGGCTCCGATGTGGCTGAAGACCCGGAGGACGCCGATATCCAAGCC

::::::::::::::::::::::::::::::::::::::::: ::::::::::::::::::

BcssDV CAGGCGGAGCTTTCAGGCTCCGATGTGGCTGAAGACCCGGAAGACGCCGATATCCAAGCC

130 140 150 160 170 180

190

BcssDV CCAAAGAGGCGCAAACA-------------------------------------------

:::::::::::::::::

BcssDV CCAAAGAGGCGCAAACAGTGAGTCACTGCCTATTTTTTTTAGACGTGCAACAAAACAATT

190 200 210 220 230 240

BcssDV ------------------------------------------------------------

BcssDV ACAGCACGTGTACACCCCGTGATACGCTATAAGAGCCTGATCATTCAGGCTGTGTCCGCT

250 260 270 280 290 300

BcssDV ------------------------------------------------------------

BcssDV CGATCCCTCCCTCGTGCCTCGGGGGGAGCCCATCTCGCTCAAGGACCTATCTTTGGCTAG

310 320 330 340 350 360

BcssDV ------------------------------------------------------------

BcssDV GGTAGGCTATTACAGGGCCTCGTCCTCCGAGCTCCCCTCCATCGTCGTTCCTCCTCAGGG

370 380 390 400 410 420

BcssDV ------------------------------------------------------------

BcssDV GGTCCCCCAGGCCGCTCGAGGATTCTCACGGCCCACATCACTATAACATCACTATATTTA

430 440 450 460 470 480

BcssDV ------------------------------------------------------------

BcssDV CAGCGCCTTACAGGCTATCACTATAACACCCTGAAGTTTCAGGGTTACACCGCTATATTT

490 500 510 520 530 540

200 210

BcssDV --------------------------------------TACCGACAGGTTTCGGATTAGG

::::::::::::::::::::::

BcssDV ACAGGCTATTACAGGCTTTAAATCGCTAACACTAACAGTACCGACAGGTTTCGGATTAGG

550 560 570 580 590 600

220 230 240 250 260 270

BcssDV GCACAACGCATAATGTTGACCTATGCCCAGATAGATGACACGTTTGATAGAGAGAAGTTC

::::::::::::::::::::::::::::::::::::::::::::::::::: ::::::::

BcssDV GCACAACGCATAATGTTGACCTATGCCCAGATAGATGACACGTTTGATAGAAAGAAGTTC

610 620 630 640 650 660

280 290 300 310 320 330

BcssDV GGACCATGGCTGAAGGAAAAGTGTGGAGCTCTGACTATAAGAGTAGCTCTCGAGGAGCAC

::::::::::::::::::::::::::::::::::::::::::::::::::::::::::::

BcssDV GGACCATGGCTGAAGGAAAAGTGTGGAGCTCTGACTATAAGAGTAGCTCTCGAGGAGCAC

670 680 690 700 710 720

340 350 360 370 380 390

BcssDV AAAGAGACCGGTGGTCTACACATCCACGCCTACGTCGAAGCACCAACACAATTCACGATC

::::::::::::::::::::::::::::::::::::::::::::::::::::::::::::

BcssDV AAAGAGACCGGTGGTCTACACATCCACGCCTACGTCGAAGCACCAACACAATTCACGATC

730 740 750 760 770 780

400 410 420 430 440 450

BcssDV AACTCTGCCGACTTTCTCGATTACTGCGGTCACCACCCCAACATACTGCCAATCAGAGTC

::::::::::::::::::::::::::::::::::::::::::::::::::::::::::::

BcssDV AACTCTGCCGACTTTCTCGATTACTGCGGTCACCACCCCAACATACTGCCAATCAGAGTC

790 800 810 820 830 840

460 470 480 490 500 510

BcssDV ACGTTTTACAAAACTTGGGATTACGTAGGCAAAGACGGAGACATCATCTTTGAAGAAGGG

::::::::::::::::::::::::::::::::::::::::::::::::::::::::::::

BcssDV ACGTTTTACAAAACTTGGGATTACGTAGGCAAAGACGGAGACATCATCTTTGAAGAAGGG

850 860 870 880 890 900

520 530 540 550 560 570

BcssDV CCGCCACCACCACGACCTGCAGAAAAATCAGGATCAGCTGCCGTGTGGACTGCCATCGTA

::::::::::::::::::::::::::::::::::::::::::::::::::::::::::::

BcssDV CCGCCACCACCACGACCTGCAGAAAAATCAGGATCAGCTGCCGTGTGGACTGCCATCGTA

910 920 930 940 950 960

580 590 600 610 620 630

BcssDV ATGGCAGCCAATACCGTCGACGAATTTTTGGAGGAGGCCTTCAAATCAAGGCCTGGGGAC

::::::::::::::::::::::::::::::::::::::::: ::::::::::::::: ::

BcssDV ATGGCAGCCAATACCGTCGACGAATTTTTGGAGGAGGCCTTGAAATCAAGGCCTGGGAAC

970 980 990 1000 1010 1020

640 650 660 670 680 690

BcssDV TTGATCAAAAACTTCACCCAATTCAAAGCCTTCGCCGAGTGGAGATACAAGCCAAAGGGG

::::::::::::::::::::::::::::::::::::::::::::::::::::::::::::

BcssDV TTGATCAAAAACTTCACCCAATTCAAAGCCTTCGCCGAGTGGAGATACAAGCCAAAGGGG

1030 1040 1050 1060 1070 1080

700 710 720 730 740 750

BcssDV TTGACATACGTGTCACCAGAGATCGTGTGTCACATGGAGGACTACCCACAACTTGAGCAG

:::::::::::::::::::::::::: :::::::::::::::::::::::::::::::::

BcssDV TTGACATACGTGTCACCAGAGATCGTATGTCACATGGAGGACTACCCACAACTTGAGCAG

1090 1100 1110 1120 1130 1140

760 770 780 790 800 810

BcssDV TGGGTATTGGAGAACTTACGAGGAGGACTGCCAGCGGGTGCCCGCAAGCAGTCATTGGTC

:::::::::::::::::::: :::::::::::::::::::::::::::::::::::::::

BcssDV TGGGTATTGGAGAACTTACGTGGAGGACTGCCAGCGGGTGCCCGCAAGCAGTCATTGGTC

1150 1160 1170 1180 1190 1200

820 830 840 850 860 870

BcssDV ATGTGGGGTGACACCAGACTGGGCAAAACACTTTGGGCTAGGTCGTTGGGCAAGCATGCA

::::::::::::::::::::::: ::::::::::::::::::::::::::::::::::::

BcssDV ATGTGGGGTGACACCAGACTGGGAAAAACACTTTGGGCTAGGTCGTTGGGCAAGCATGCA

1210 1220 1230 1240 1250 1260

880 890 900 910 920 930

BcssDV TACTTCCCAGGCATGTTTATGTTGGACGGTTTCAACGAGGAGGGCTGCGAGTATGCCATC

::::::::::::::::::::::::::::::::::::::::::::::::::::::::::::

BcssDV TACTTCCCAGGCATGTTTATGTTGGACGGTTTCAACGAGGAGGGCTGCGAGTATGCCATC

1270 1280 1290 1300 1310 1320

940 950 960 970 980 990

BcssDV TTCGATGACATAATCGAAGGATTCAAAGGCATCGCCAGCTACAAGGGGTGGTTTGGGTCT

::::::::::::::::::::::::::::::::::::::::::::::::::::::::::::

BcssDV TTCGATGACATAATCGAAGGATTCAAAGGCATCGCCAGCTACAAGGGGTGGTTTGGGTCT

1330 1340 1350 1360 1370 1380

1000 1010 1020 1030 1040 1050

BcssDV CAACATGAGGTCGTCGTCACCGACAAGTACCGTATGAAGCAGAGAATCACGTGGGGAAGA

::::::::::::::::::::::::::::::::::::::::::::::::::::::::::::

BcssDV CAACATGAGGTCGTCGTCACCGACAAGTACCGTATGAAGCAGAGAATCACGTGGGGAAGA

1390 1400 1410 1420 1430 1440

1060 1070 1080 1090 1100 1110

BcssDV CCTTCGGTCTTCATCAGCAACACTGATCCACGTGACGATTTGCCTAGAGATCAAGTCAAA

::::::::::::::::::::::::::::::::::::::::::::::::::::::::::::

BcssDV CCTTCGGTCTTCATCAGCAACACTGATCCACGTGACGATTTGCCTAGAGATCAAGTCAAA

1450 1460 1470 1480 1490 1500

1120 1130 1140 1150 1160 1170

BcssDV TGGTTGGAGGGAAATTGTGTTTTTGTGCATATTGATAAAGTACTATGCGAGCCGTATGTA

:::::::::::::::::::::::::::::: :::::::::::::::::::::::::::::

BcssDV TGGTTGGAGGGAAATTGTGTTTTTGTGCATGTTGATAAAGTACTATGCGAGCCGTATGTA

1510 1520 1530 1540 1550 1560

1180 1190 1200 1210 1220 1230

BcssDV GGTGAATCACCAACATAGGCGCCCAAAAAAA-TAATTAATTCATTTCATGTCATGTGACT

::::::::::::::::::::::::::::::: ::::::::::::::::::::::::::::

BcssDV GGTGAATCACCAACATAGGCGCCCAAAAAAAATAATTAATTCATTTCATGTCATGTGACT

1570 1580 1590 1600 1610 1620

1240 1250 1260 1270 1280 1290

BcssDV TCGTCCCTCTCTGTCAGAATATTTGCTATAAAGTGTGGTCATGTCCAAACGCAAATGAAG

::::::::::::::::::::::::::::::::::::::::::::::::::::::::::::

BcssDV TCGTCCCTCTCTGTCAGAATATTTGCTATAAAGTGTGGTCATGTCCAAACGCAAATGAAG

1630 1640 1650 1660 1670 1680

1300 1310

BcssDV AGTAAAATTTTAAG

::::::::::::::

BcssDV AGTAAAATTTTAAG

1690
