# Supplementary material for: Novel Mycoviruses Discovered in the Mycovirome of a Necrotrophic Fungus
Source: mBio. 2021 May 11;12(3):e03705-20. doi: 10.1128/mBio.03705-20 (PMC8262958; doi:10.1128/mBio.03705-20)
Supplement: TABLE S2 [file mbio.03705-20-st002.docx]

| **MYCOVIRUSES** | **FORWARD qPCR** | **REVERSE qPCR** |
| --- | --- | --- |
| **Botrytis cinerea mycovirus 3 RNA1** | GAGGACTTGGCTACGGTCAA | GCGTCTTGAGTCCCATCACT |
| **Botrytis cinerea mycovirus 3 RNA2** | CCATCTGGAAGACGGAGCTA | GACTGGCAGCATGTAAGTGG |
| **Botrytis cinerea hypovirus 2** | GCATGATGAAGAGCCAGTGA | GGGCTATCACCAAATTCCAA |
| **Botrytis cinerea hypovirus 3** | CGACGCCCTTTAAAATACCA | TGCGTCATACAAGCAGGAAG |
| **Botrytis cinerea hypovirus 4** | GGACGAGGACACACTTTGGT | GAACGCAGGTGTTGCAGTTA |
| **Botrytis cinerea umbra-like virus 1** | CAGCGAGTCATTTGTCAGGA | ACGGTCAAAGCTACGTCCTC |
| **Botrytis cinerea endornavirus 2** | TGTCGAGTACCCCATGTTCA | GGGTCCTATCCAAACCAACA |
| **Botrytis cinerea endornavirus 3** | GGCAGGTGTGACAAAAGGTT | GAAAGGCAGTGCAAATGGTT |
| **Botrytis cinerea fusarivirus 3** | CTCGTGACAGACGCACAAGT | GGGGACCGTGCCTCTATATT |
| **Botrytis cinerea fusarivirus 5** | CACGTGCAGTCGATCATCTT | CCGATGTCTTGCTCATTGAA |
| **Botrytis cinerea fusarivirus 7** | GCAGAATCACACCCATCAGA | TTTGCAGAAGGAGCAAAAGG |
| **Botrytis cinerea botybirnavirus 2 RNA1** | CGAAGTCGAGTAAGGGCAAG | ACACATCGCCCTCTTCAAAT |
| **Botrytis cinerea botybirnavirus 2 RNA2** | CAACAACACTGGCAAACACC | TTGCCATCCATTCACGACTA |
| **Botrytis cinerea bocivirus 1 RNA1** | CAGCAGATGCAGCTAAGTGG | GGATGGGAGAAATGAAGCAA |
| **Botrytis cinerea bocivirus 1 RNA2** | TCCCTATGGAAAAGCTGTGG | CATGAAGACTTCCCTGCAAGA |
| **Botrytis cinerea bocivirus 1 RNA3** | GCATGTGCACCATTGCTATC | GACCTCTTTCCAGCGTCTGA |
| **Botrytis cinerea orthobunya-like virus 1** | CAGCACTTTCGTGTCCAAAA | TTTGCAAGCCTTAGGAGACA |
| **Botrytis cinerea mitovirus 6** | CAATGCCTCAGTGATCGAAA | TACCTTTCCTGCTGGCTCAT |
| **Botrytis cinerea ourmia-like virus 9** | CTGGCTTCTTGTTGGTAGCC | GAGTGCCACACCTTTCCTTC |
| **Botrytis cinerea alpha-like virus 1** | CTGTGTCCGGCACTCAATTA | TCCATAACGCCAACAATTCA |
| **Botrytis porri botybirnavirus 1 RNA1** | GCCTGTGGAAGCAGAAGAAG | CCTTCTACCAGGCTGTCTGC |
| **Botrytis porri botybirnavirus 1 RNA2** | CTGTCTACTCGGGCTCAAGG | GCAAATAGCAATGCAAACGA |
| **Botrytis cinerea mycovirus 5 RNA1** | CTCAACGCCAAGTACGACAA | AGCTTCTCATCGAGCCTCAC |
| **Botrytis cinerea mycovirus 5 RNA2** | CGGCTTCAGGACTTCGATAA | ATCCAATGTCGGCATAGAGC |
| **Botrytis cinerea victorivirus 2_BCS9** | TGGCTCACTGTTGGTTGAAG | TGACCGTATCTCGTTGGTCA |
| **Botrytis cinerea victorivirus 2_BCS14** | ACACCCTTAGTGACGCTGCT | TTGTTTTGTGGGGTTCATCC |
| **Botryotinia fuckeliana partitivirus 1 RNA1** | AGGTCGTTTCAATCCAACCA | TCGTCACGTTCTGACATTCC |
| **Botryotinia fuckeliana partitivirus 1 RNA2** | ACGGGTAAGACAGGATGTGC | CACCTTTTGTGTGTCCGTCA |
| **Botrytis cinerea mitovirus 9** | TGGGATTTAACCGCAAATCA | ACTATTCGGACCAGCAGCTT |
| **Botrytis cinerea mitovirus 5** | TGGACAACCTATGGGAGCTT | TGCGGCTCATGCAACTACTA |
| **Sclerotinia sclerotiorum mitovirus 4** | GGCTCATGCAACGACAAGAT | GGTCAACCAATGGGAGCTTA |
| **Botrytis cinerea victorivirus 3** | GAGCGATATTCGCCTGTGAT | CCACCTCTTCTGGACGTCAT |
| **Botrytis cinerea ssDNA virus 1** | GTCGACGAATTTTTGGAGGA | GAAGGCTTTGAATTGGGTGA |
| **Botrytis cinerea binarnavirus 1** | ATGGAAGATCCATGCTGCTC | GCCAGACCACTAGCTCAGGA |
| **Botrytis cinerea ourmia-like virus 11** | CCTTTCTCTGCCTGCTCAAT | CCTACACGCCTTTCACCTTC |
| **Botrytis cinerea ourmia-like virus 16** | GCTTACGGATTTGGAACGAG | GGCCAGAGTCCTGCAAGTTA |
| **Botrytis cinerea negative-stranded RNA virus 3** | CGGCATTCCAATCGCTATAC | CGCAGATCGAGAAAGAATCC |
| **Botrytis cinerea negative-stranded RNA virus 6** | GATGTTCCACAAGCTCAGCA | CACGTCCTTGATCACCTCCT |
| **Botrytis cinerea flexivirus 1** | ACTCCTCGCCTGCTGATAGA | ATCGGTATACAGGCCACTCG |
| **Botrytis cinerea deltaflexivirus 1** | CAAGGCAACGTTTTTCTCGT | TGACTGTTGCCATACGTGGT |
| **Botrytis cinerea binarnavirus 2 RdRp** | ACGGAAGTGGTTCAAGATGC | CGATAGGTATGTCGCCGTCT |
| **Botrytis cinerea binarnavirus 2 HP** | AGAGTCCCCACTCTCCGTCT | CCGTATTCCATGTCCTCGTC |
| **Botrytis cinerea narnavirus 4** | GAGCCCTTCTCCAGAAATCC | AGCTTCACGAATGGCATT |
| **Botrytis cinerea binarnavirus 5** | CCTTCAACGGTTCCTCTCAT | GAAATGCCCTGAAGGACAAA |
| **Botrytis cinerea mycovirus 4 RNA1** | GCAGGCACGTTACACAAATG | GCACCTCGTGTGATTGCTTA |
| **Botrytis cinerea mycovirus 4 RNA2** | ATGCGCAAGGTAGTTTCACC | CTTTGGTTTGGGGCATAAGA |
| **Botrytis cinerea mycovirus 4 RNA3** | GATCCCGTAACAACGCAAGT | TGCCCAAGATGTTCCACATA |
| **Botrytis cinerea mycovirus 4 RNA4** | GCATGCACCGAGAAGACATA | GGCATCGAATCTGGTCACTT |
|  |  |  |
| **PRIMER NAME** | **SEQUENCE** |  |
| **virssDNArep Forward** | CTCTTATAGTCAGAGCTCCACAC |  |
| **virssDNArep Reverse** | CACGTGACGATTTGCCTAGAGATC |  |
| **Gem_Bc_det_F** | GTAACCCTGAAACTTCAGGG |  |
| **Gem_Bc_det_R** | ATTGCCCGCTATTAGGCGTG |  |
| **BcBNV2_HP_det_Fw** | CGTACCCCCTTTGAGAATCA |  |
| **BcBNV2_HP_det_Rev** | AGACGGATTCCTCCAACCTT |  |
| **BcBNV2_rep_det_Fw** | GACCCAAGTGGGAAGGAAAC |  |
| **BcBNV2_rep_det_Rev** | GGAGGCTCTTCCAAGACTGAATC |  |
| **BcBNV2_pro_Fw_rep** | CTCACTGCTGACATACACCGTAA |  |
| **BcBNV2_pro_Rev_rep** | TGTAGTCCAGACAGGACCAGAAT |  |
| **BcBNV2_pro_Fw_HP** | CCATCGGCTAAGAGACAAGC |  |
| **BcBNV2_pro_Rev_HP** | ACCGTGGAATAGCCACTGAG |  |
| **BcBV1_RNA1_Fw** | GTCACCAGAATCTAAGGAGTTGG |  |
| **BcBV1_RNA1_Rev** | GTGTCCTATGCTTTTCATCATCAG |  |
| **BcBV1_RNA2_Fw** | GGGGTACAGTCTCACTTGCA |  |
| **BcBV1_RNA2_Rev** | TCTCCAAGATGTTCTGGTCCT |  |
| **BcBV1_RNA3_Fw** | AGCTTTCCCTGGTTCCATGA |  |
| **BcBV1_RNA3_Rev** | ATGGCGAGGCATGAGTAGAA |  |
| **BcBNV2_rep_RACE5_gs_out** | GACCCTCAGGTTCCGATTATAC |  |
| **BcBNV2_rep_RACE5_gs_in** | GACACCTCCTCGCTTTGTGAT |  |
| **BcBNV2_rep_RACE3_gs_out** | GAAGCTTTTATCCGTGGGATG |  |
| **BcBNV2_rep_RACE3_gs_in** | GGTCGGACGAGGAGATACAC |  |
| **BcBNV2_HP_RACE5_gs_out** | CTCCCGATCTGTTCAATTTCGTC |  |
| **BcBNV2_HP_RACE5_gs_in** | CCGTATTCCATGTCCTCGTCAA |  |
| **BcBNV2_HP_RACE3_gs_out** | TCTGCACGAGCCGATATCAAGAC |  |
| **BcBNV2_HP_RACE3_gs_in** | GGCGGAGGAACCAGACCATC |  |
| **BcBV1_RNA1_RACE_3’out** | CCACCATCTAGCAGATCAGCAAC |  |
| **BcBV1_RNA1_RACE_3’in** | GGTTTGCCATCAGTCCCTACATT |  |
| **BcBV1_RNA1_RACE_5’out** | CATGTTGAGGTGATGTGCAA |  |
| **BcBV1_RNA1_RACE_5’in** | GGGAAGCTATTGTAAAACCACA |  |
| **BcBV1_RNA2_RACE_3’out** | CCACTTGAGCATGTTCGTGATTT |  |
| **BcBV1_RNA2_RACE_3’in** | GATTGACCATTGCGCTTAAAGGA |  |
| **BcBV1_RNA2_RACE_5’out** | CTAGCAGCAAGAAACCCGATCTT |  |
| **BcBV1_RNA2_RACE_5’in** | GTGTTGAATCCTTTAGACCAGCGT |  |
| **BcBV1_RNA3_RACE_3’out** | GTCCCTCTGTTGCTCTCTCAA |  |
| **BcBV1_RNA3_RACE_3’in** | CAGCATCTGCTTTGATGAACTCA |  |
| **BcBV1_RNA3_RACE_5’out** | CTACTCATGCCTCGCCATTC |  |
| **BcBV1_RNA3_RACE_5’in** | GGTCCTTATCAGCTGACTACGA |  |
